# Multi‐Stimuli Responsive Angstrom‐Scale Two‐Dimensional Channels of Natural Layered Materials

**DOI:** 10.1002/adma.74804

**Published:** 2026-08-26

**Authors:** Raj Kumar Gogoi, Arghyadeep Paul, Ankit Bhardwaj, Hiran Jyothilal, Narayana R. Aluru, Boya Radha

**Affiliations:** ^1^ Department of Physics & Astronomy School of Natural Sciences University of Manchester Manchester UK; ^2^ National Graphene Institute The University of Manchester Manchester UK; ^3^ Department of Mechanical Science and Engineering Beckman Institute for Advanced Science and Technology University of Illinois at Urbana−Champaign Urbana Illinois USA; ^4^ Walker Department of Mechanical Engineering The University of Texas at Austin Austin Texas USA; ^5^ Photon Science Institute The University of Manchester Manchester UK

**Keywords:** Å‐scale channel, electro‐mechanical ion transport, multi‐responsiveness, nanofluidics, smart ion channels, streaming current, vermiculite

## Abstract

Biological ion channels are nature's wonders that regulate complex and multifunctional molecular mechanisms in living things. Emulating such multifunctionality in artificial systems remains a formidable challenge due to the limitations in achieving concurrent multi‐stimuli responsiveness. Here, we report biomimetic and multi‐responsive two‐dimensional (2D) ion channels with Angstrom (Å)‐scale channel heights, naturally formed within the interlayer galleries of vermiculite (VM) clay, which inherently possesses negatively charged surfaces. The VM ion channels exhibit electrohydrodynamic ion transport that can be modulated by mechanical (pressure), chemical (pH), and electrical (voltage) stimuli, thereby replicating key gating functions of the biological channels. Under coupled electric field and pressure gradients, the channels display stimulus‐dependent polarity inversion of streaming current, governed by surface charge modulation and the redistribution of mobile ions within the confined spaces. To describe this coupled effect, we developed a modified surface charge regulation (*mSCR*) model that incorporates voltage‐, pressure‐, and concentration‐dependent charge modulation. This study lays the foundation for clay minerals to be utilized as versatile platforms for realizing multi‐stimuli responsive nanofluidic systems, opening new avenues for adaptive ionic gating, biosensing, energy conversion, and therapeutic nanofluidics.

## Graphical abstract

Å‐scale ion channels naturally formed within the interlayers of vermiculite clay enable ionic current modulation in response to mechanical, chemical, and electrical stimuli. Coupled pressure and electric fields induce reversible streaming current (*I*_stream_) polarity inversion, which is explained by a modified surface charge regulation model. This work demonstrates natural clay minerals as a promising platform for adaptive, multi‐stimuli responsive nanofluidic systems.

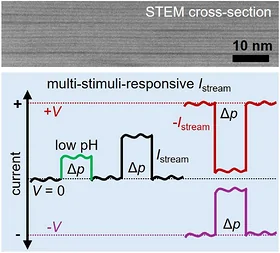

## 1 Introduction

Biomimetic smart ion channels, responding to external stimuli, offer precise routes of mass transport analogous to those of biological ion channels. Their ability to detect minuscule changes in their environment makes them invaluable for analytical sensing, diagnostics, robotics, and biosensing to extends beyond human reach [1, 2, 3, 4, 5]. Both physical and chemical stimuli have been used to induce responses in such channels, with each stimulus offering a distinct control mechanism, selectively targeting specific applications [6, 7]. Physical stimuli such as light, temperature, pressure, magnetic field, and electric field are non‐contact in nature. While their responses are highly reversible, they typically provide moderate selectivity. Chemical stimuli like pH, ligands, and specific ions and molecules, on the other hand, offer high selectivity but necessitate direct contact with the channels. While smart ion channels responsive to single stimuli have been extensively studied in recent decades, the development of multi‐responsive channels capable of reacting to combinations of stimuli remains limited. Only a handful of dual‐responsive smart nanochannels, responsive to combinations of physical and chemical stimuli [8, 9, 10, 11, 12, 13, 14, 15, 16, 17, 18], and dual physical stimuli [19, 20, 21, 22], can be found in the literature. Nevertheless, the search for more such multi‐stimuli responsive nanoscale conduits, which can potentially display astonishing multi‐dimensional properties that closely mimic those of their biological counterparts, has gained significant attention in the field of nano‐ and angstrom (Å)‐scale fluidics.

Recent advances in nano‐ and Angstrom (Å)‐scale fluidic channels have revealed exotic flow behaviors; however, most of these systems are limited to dual responsiveness [8, 9, 10, 11, 12, 13, 14, 15, 16, 17, 18, 19, 20, 21, 22], lacking the multi‐responsive capabilities of biological ion channels. Bridging this gap is critical to advancing the functionality of artificial ion channels and replicating the complexity of their natural counterparts. This study seeks to address this challenge by harnessing naturally occurring channels with multi‐responsive behavior. Specifically, we have developed an innovative approach to exploit the confined spaces between individual layers in vermiculite (VM) clay, utilizing these Å‐scale two‐dimensional (2D) ion channels and their intrinsic surface charges to achieve multi‐stimuli responsiveness.

VM clay, used as the model system, is a smectite group of clay, characterized by a highly ordered and negatively charged layered structure. The free space between individual clay layers can serve as Å‐scale fluidic channels. We inspect the electrohydrodynamic ion transport through these VM channels, with VM offering the advantages of natural abundance, chemical and thermal stability, favorable dielectric properties, and, most importantly, the presence of natural ion channels between its layers. Although there have been substantial efforts to produce nanochannels and artificially intelligent systems of clay [23, 24, 25, 26, 27, 28], their potential as smart ion channels remain unexplored. Remarkably, our experiments reveal a novel voltage‐driven regulation of the hydrodynamic streaming of ions in the VM channels, where the polarity of the streaming current can be modulated with the electric field and the ionic strength of the electrolyte.

## 2 Result and Discussion

Here, we design Å‐scale ion channels by utilizing the naturally occurring confined free‐spaces between the adjacent layers in VM crystals (Figure 1A, detailed in section S2 of supporting information (SI)). This VM channel device, when exposed to multiple stimuli (Figure 1B), responds with varying streaming currents (*I*_stream_). The interlayer spacing (*d*_spacing_) of the VM layers at ambient atmosphere is ∼14.3 Å, estimated from the XRD analysis (Figure 1C) of the VM crystals. The step heights of the VM layers, estimated by atomic force microscopy (AFM) at the edge of a thin VM flake under ambient conditions, show a height difference of ∼14 to 17 Å, Figure 1D. The layered nature of the VM clay that enables the formation of 2D ion channels is visible from the cross‐sectional high‐angle annular dark‐field scanning transmission electron microscopy (HAADF–STEM) in Figure 1E. In contrast to the XRD and AFM, STEM analysis shows smaller value of interlayer distances ∼8–11 Å; this is likely because the STEM measurements were performed under high‐vacuum conditions, resulting in the removal of interlayer water molecules that are present in VM under ambient conditions [29, 30, 31]. The VM channel height (*H*) in our devices is estimated as ∼6.3 Å considering the *d*_spacing_ ∼14.3 Å and thickness of a VM layer as ∼8 Å (detailed in Section S1.4 in Supporting Information).

**FIGURE 1:**
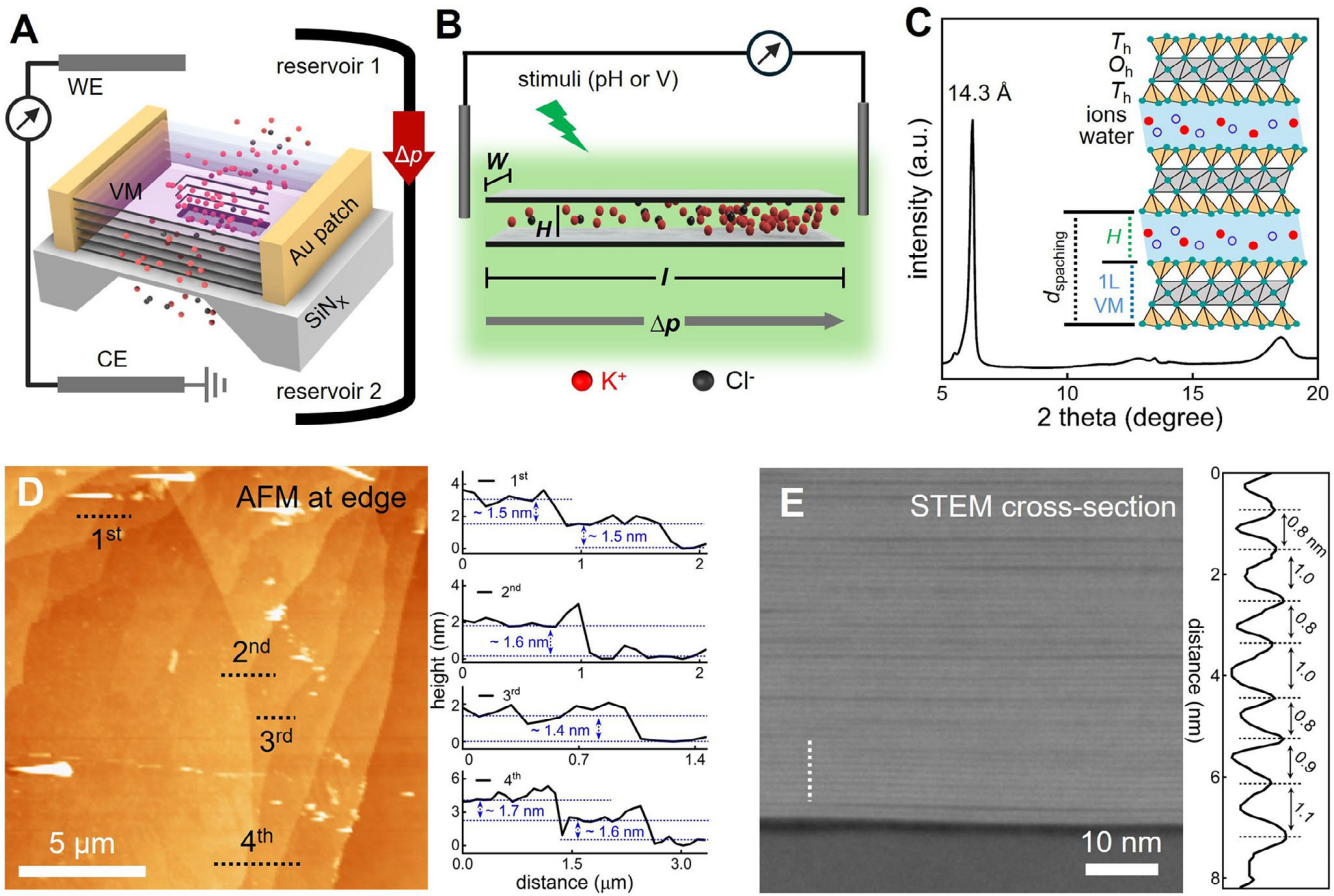
Molecular streaming through VM fluidic devices. (A) Schematic of the experimental setup used to perform electrochemical experiments through the VM channels. Δ*p* denotes the applied pressure. *WE* and *CE* refer to the working and counter electrodes, respectively, which are Ag/AgCl electrodes. (B) Schematic representation of the stimuli‐responsive ion transport inside the VM channels. (C) pXRD of VM crystals with inset showing the clay structure. (D) Atomic force microscopy image of the edge of a VM flake with the corresponding height profiles measured at different positions (black dotted lines). (E) Cross‐sectional high‐angle annular dark‐field scanning transmission electron microscopy (HAADF–STEM) image of a VM flake with the corresponding intensity profile extracted along the dotted line.

These channels show surface‐charge‐governed ion transport, with the conductance (*G*) remaining nearly constant with varying reservoir salt (KCl) concentration (*C*_0_) in the low concentration range (Section S5, Figures S3D and S4 in Supporting Information). The ion flux is primarily dominated by cations (section S6 and Figure S5). The stimuli‐responsiveness of the VM channels is then investigated by measuring the variation of the ionic current (*I*) flowing through them with a pressure difference (*∆p*) applied across the device (schematic in Figure 1A). As *∆p* increases, |*I*| also increases (Figure 2A) for 1 mM KCl, at pressure increments of 100 mbar up to 500 mbar. The streaming current is calculated as: *I*_stream_ = *I*_Δ*p*_  − *I*_0_, where *I*_0_ and *I*_∆_ *_p_* are the ionic currents before and after applying pressure. Variation of *I*_stream_ with *∆p* for 0.1, 1, 10, and 100 mM KCl are in Figure 2B. The streaming mobility (*µ*_stream_) is calculated from the slope of these curves, $\mu_{\mathrm{stream}}=\frac{I_{\mathrm{stream}}.l}{\Delta p.A}$, where *A* (= *N*.*w*.*H*) is total channel cross‐sectional area (*l*, w, *H* and *N* are the length, width, height, and number of the channels, respectively) [19]. Figure 2C shows that *µ*_stream_ is the highest for 1 mM KCl. *µ*_stream_ reduces when *C*_0_ increases from 1 to 100 mM, which can be attributed to the decreasing ion selectivity ($t_{K^{+}}$) with higher *C*_0_ (Figure S5B) due to elevated surface charge screening. However, *µ*_stream_ (*C*_0_ = 0.1 mM) < *µ*_stream_ (*C*_0_ = 1 mM) due to the plummeting number of charge carriers with extreme reduction in *C*_0_. The apparent zeta potential (Figure 1F, right *y*‐axis), estimated as: $\zeta =-\frac{\mu_{\mathrm{stream}}.\eta }{\in .\in_{o}}$, at viscosity, η = 1 mPa.s, dielectric permittivity, ∈ = 80 and vacuum permittivity [19], $\in_{o}$ = 8.85 × 10^−12^ F.m^−1^, turns out to be in the range of −22.5 to −31.3 mV, suggesting the negative surface charge of the channels’ walls. Here, we use ∈ of bulk water; however, literature [32], shows that confinement can significantly suppress ∈, which would further increase the estimate of ζ.

**FIGURE 2:**
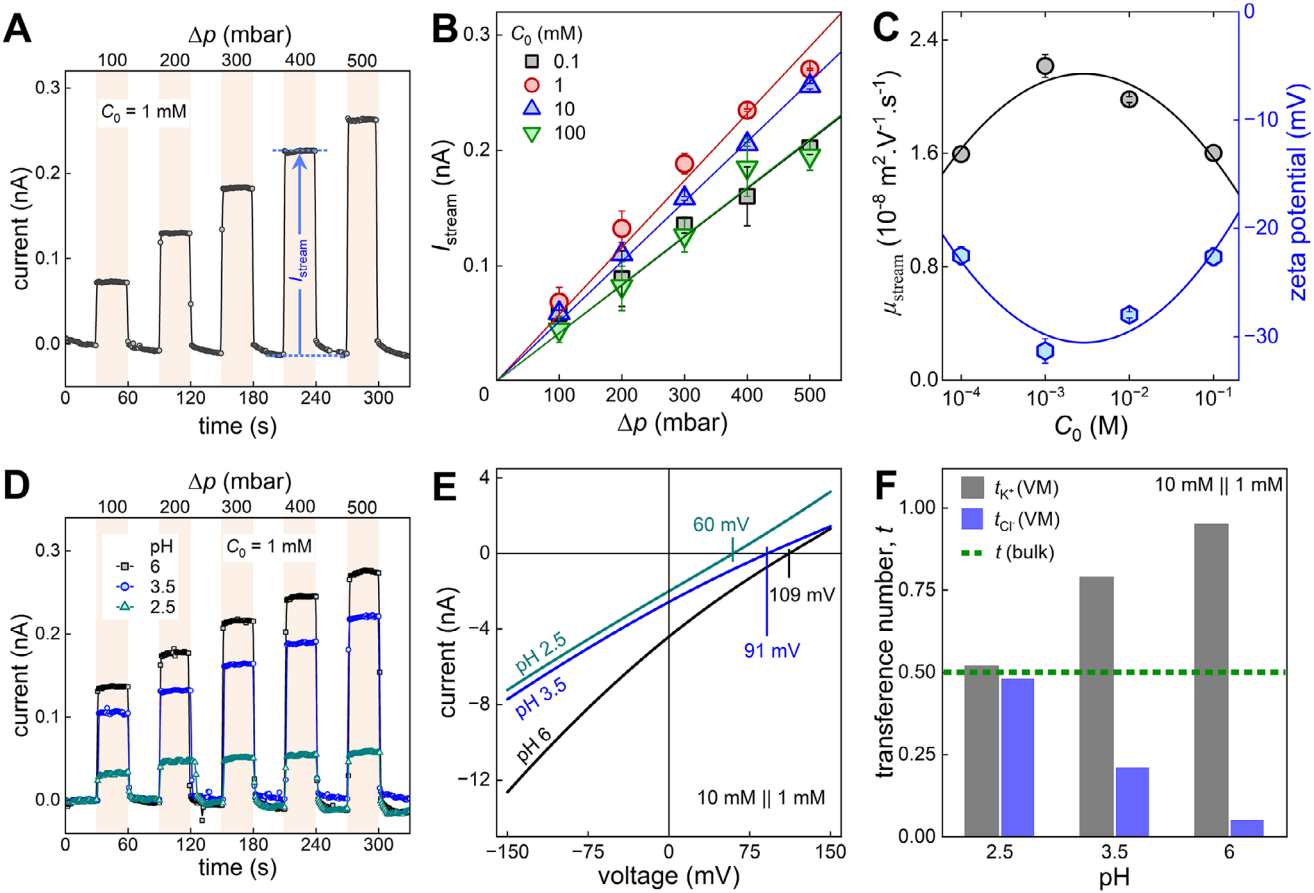
Molecular streaming in VM channels devices and the effects of pH (A) Ionic current versus time at different Δ*p* for *C*_0_ = 1 mM KCl. (B) *I*_stream_ *vs* Δ*p* at different *C*_0_ (0.1, 1, 10, and 100 mM KCl). Error bars represent the uncertainty in the *I*_stream_ for that particular Δ*p*. (C) The variations of streaming mobility (black plot) and zeta potential (blue plot) with *C*_0_. Error bars represent the uncertainty of fitted values (slopes) in (B). (D) pH‐dependent ionic current *vs* time at different Δ*p*. (E) *IV* curves and (F) the corresponding transference numbers of the ions flowing through the VM channels at different pH with *C*_0_ of 1 mM KCl and 10 mM KCl in the permeate and feed reservoirs (2 mL each), respectively. Reservoirs 1 and 2 (Figure 1A) are used as the permeate and feed reservoirs, respectively. *Device #2* is used in (A), (B), and (C), and *Device #7* is used in (D), (E) and (F). The device dimensions are detailed in Table S1 in Supporting Information.

pH‐dependent ion channels are essential for adaptive cellular physiological responses to environmental pH changes. They play a critical role in detecting diseases such as cancer, chronic pain, and heart disease as these ailments affect the pH levels in the body. The potential of the VM ion channels for diagnostic applications is explored by examining their pH responsiveness in relation to *I*_stream_ using 1 mM KCl solutions at pH = 6, 3.5, and 2.5. As the pH decreases, *I*_stream_ also decreases (Figure 2D), which is attributed to a reduction in the ion selectivity of the VM channels that normally arises from the negative surface charges (−0.6 to −0.9 charges per unit cell) [33], in VM layers due to the substitution of Si^4+^ by lower valence cations like Al^3+^. We assess this selectivity at different pH in terms of $t_{K^{+}}$ by performing drift‐diffusion experiments under a KCl concentration gradient of 10 (*C*_feed_ = 10 mM and *C*_permeate_ = 1 mM, Figure 2E). Decreasing the pH from 6 to 2.5 reduces $t_{K^{+}}$ from ∼ 0.95 to ∼ 0.52, indicating that the cation selectivity of the VM channels reduces with decreasing pH (Figure 2F). This occurs because lowering the pH increases the concentration of H^+^ ions inside the channels, which tend to neutralize the negative surface charges of the VM channels, thereby reducing ion selectivity and the net pressure‐driven current.

Next, we focus on the dual‐(physical‐)stimuli response under simultaneously applied electric field and pressure. When Δ*p* = 0, as expected, applying a positive voltage (Δ*V*) yields a positive *I*. However, the *I* decreases when Δ*p* is applied in the same direction as that of the electric field, generating a negative *I*_stream_ (Figure 3A,B). Conversely, a negative Δ*V* shifts *I* to a negative value. Now, when Δ*p* is applied in the same direction as before, *I* increases (stays negative but reduces in magnitude), yielding positive *I*_stream_ (Figure 3A,B). Hence, *I*_stream_ depends on both stimuli (Figure 3B,C). Mathematically, this dual‐stimuli dependency can be expressed as the quadratically decreasing function of *I*_stream_ with Δ*V*:

(1) $$I_{\mathrm{stream}}^{\Delta p}\left(\Delta V\right)=I_{\max}\;\left[1-\omega {\left(\frac{\Delta V+V_{\mathrm{eq}}}{V_{t}}\right)}^{2}\right]$$

where *I*_max_ is the maximum positive current the channel can generate, *ω* is a (dimensionless) scaling factor that determines the non‐linearity of the *I*_stream_–*V* curve, *V*_eq_ is the characteristic potential that adjusts the equilibrium of the system, and *V*_t_ = *k.T*/*e* = 25 mV is the thermal voltage. The parameters *I*_max_, *ω*, and *V*_eq_, which depend on *∆p*, are derived by fitting *I*_stream_ as a function of Δ*V*. The similarity of the derived *I*_max_ with the experimental *I*_max_ values for 1 mM KCl electrolyte under *∆p* = 100, 200, 300, 400, and 500 mbar validate the mathematical fitting (inset Figure 3C and the error in S8B).

**FIGURE 3:**
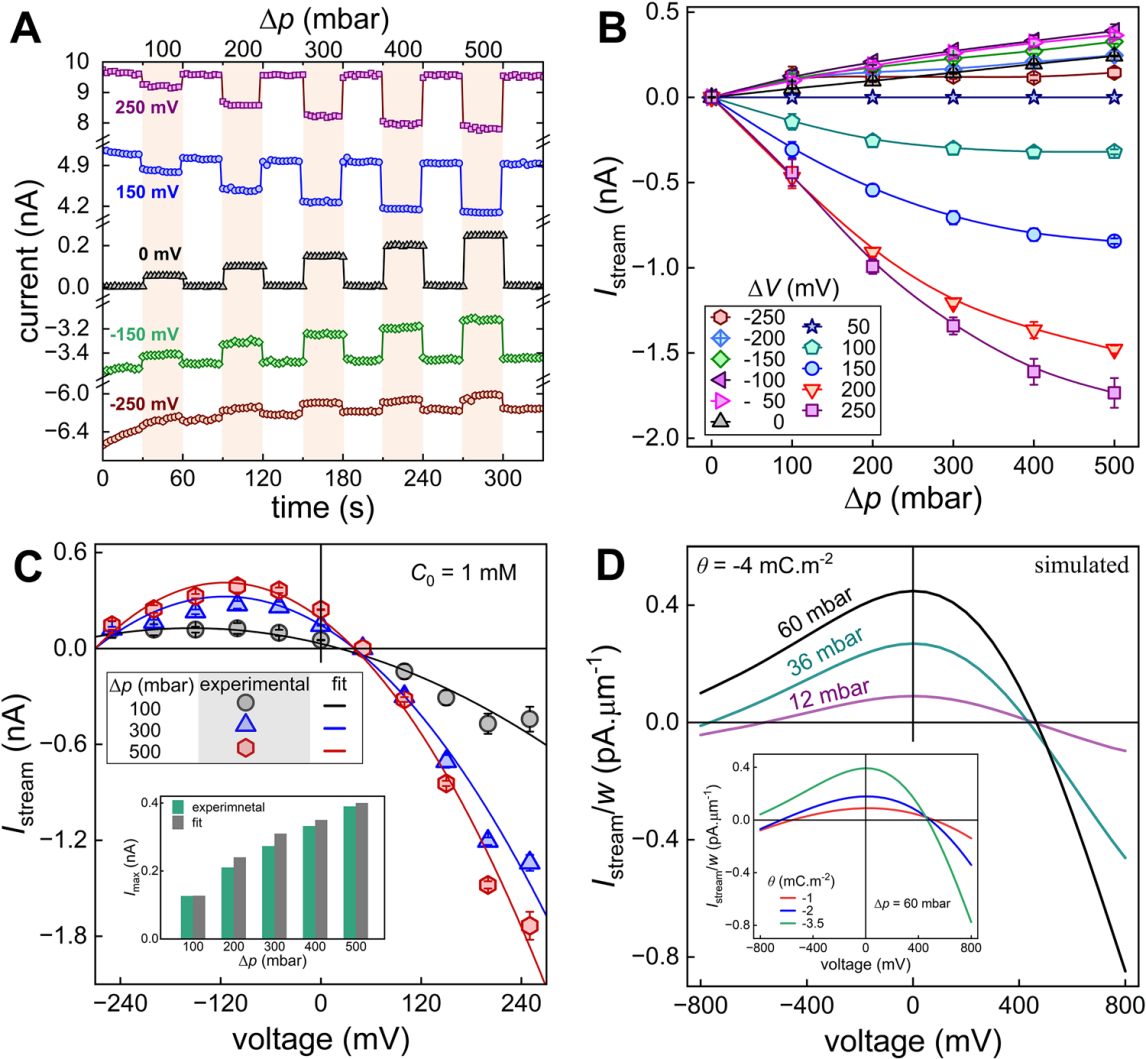
Effect of electric field on mechanical response: (A) Ionic current *vs* time at different Δ*p* and Δ*V* (Figure S8A has the full dataset). (B) *I*_stream_–Δ*p* curves at varying Δ*V*. (C) *I*_stream_–*V* curves at *∆p* = 100, 300 and 500 mbar. Inset in (C) compares the experimental and derived *I*_max_; the latter is obtained via the fitting curve in Equation (1). *Device #4* is used in (A), (B), and (C) with *C*_0_ = 1 mM KCl (2 mL electrolyte used in each reservoir) and the pH is measured to be ∼6. (D) The variation of numerically simulated streaming current per unit channel width, *I*_stream_/*w*, with *∆V*, in a system outlined in Figure S12 for different *∆p* at *θ* = −4 mC.m^−2^ and (in the inset) for varying *θ* at *∆p* = 60 mbar, with channel height (*H*)= 2 nm, channel lenght (*l*) = 0.26 µm, and *C*_0_ = 1 mM KCl. Error bars in (B) and (C) represent the uncertainty in the measured *I*_stream_ for a particular Δ*p*.

To elucidate the origins of the dual‐stimuli dependent *I*_stream_, we perform finite‐element simulations of ionic flow across a nanochannel (Figure S12) using a continuum framework based on the Poisson, Nernst–Planck, and Navier–Stokes (PNP–NS) equations (Section S9). The reasons behind the selection of this computational method are elaborated in Section S9 of Supporting Information. The continuum PNP–NS model has previously been shown to accurately capture and analyze the ion transport behavior in nanopores with diameter as small as 2 nm [20, 34]. However, as the continuum assumptions break down under Å‐scale confinements, the experimental *H* of 6.3 Å cannot be reliably represented within the PNP–NS framework. As a result, a *H* of 2 nm is adopted in our numerical simulations, as it is the smallest feasible dimension at which these simulations can be carried out. Importantly, this *H* is sufficiently small to ensure significant overlap of the electrical double layers [35], which is inevitable in the Å‐scale channels and is known to greatly influence ionic flow. Therefore, we are able to preserve the key electrokinetic characteristics of the Å‐scale channels in our numerical simulations. Considering the surface charge density (*θ*) ∼ −1.4 mC.m^−2^ reported for VM channels in the literature [23], simulations were performed with *θ* = −0.2 to −4 mC.m^−2^. The resulting *I*_stream_‐*V* curves vary noticeably with *θ* (Figure S13 and Figure 3D). Weaker *θ* yield nearly symmetric parabolic curves, whereas stronger *θ* yield asymmetric curves with steeper slopes at Δ*V* > 0 than those at Δ*V* < 0. The simulated *I_stream_*–*V* curves (Figure 3D) closely mimic the experimental *I*_stream_–*V* curves (Figure 3C) near *θ* ∼ −4 mC.m^−2^. Moreover, *θ* estimated from the *I*_stream_–*V*​ curves at Δ*V* = 0 in Figure 3C using the Grahame equation confirms the consistency between the experimental and the numerical observations. Here, ionic current (*I*) across the channel can be expressed as:

(2) $$I\;\left(\Delta V,\;\Delta p\right)=G.\Delta V+\mu .\Delta p=\left(G_{0}.\Delta V+G_{p}.\Delta V\right)+\mu .\Delta p,$$

where *G*.Δ*V* is the voltage‐driven current and *μ*.Δ*p* is the current arising from the advection of net excess counterions by the Δ*p*‐driven fluid flow [36, 37, 38], *G*, here, is the ionic conductance. As Δ*p* perturbs the quasi‐equilibrium distribution of ions in the channel, thereby altering *G* [36, 37, 38], *G* can be expressed as the sum of a pressure‐dependent conductance (*G*_P_) and a pressure‐independent conductance (*G*_0_). So, *I*(Δ*V*, Δ*p* = 0) = *G*_0_.Δ*V*. Based on this formulation, *I*_stream_(Δ*V*, Δ*p*) can be expressed as:

(3) $$\begin{array}{cc} I_{\mathrm{stream}}\;\left(\Delta V,\;\Delta p\right) & =I\left(\Delta V,\;\Delta p\right)-\;I\;\left(\Delta V,\;\Delta p=0\right)\\ & =G_{p}.\Delta V+\mu .\Delta p \end{array}$$

The *μ*.Δ*p* term (*i.e*., *I*_stream_ at Δ*V* = 0) is positive (Figures 2B and 3B,C) due to the VM channel's intrinsic cation selectivity (Figure S5B), originating from the channels’ negative *θ* and facilitating the predominant advection of K^+^ ions by Δ*p*. As *μ*.Δ*p* is independent of Δ*V*, the change in *I*_stream_ with Δ*V* is generated by *G*_p_.Δ*V*, which is the change in the voltage‐driven current (*G*.Δ*V*) due to Δ*p*. Hence, *G*_p_ is essentially the change induced in *G* by the application of Δ*p*.

The ionic concentration profiles in Figure 4B,C and Figure S15 provide further insight on the variation of *I*_stream_ with Δ*V* in Figure 3D. At Δ*V* = 0.4 V, when Δ*p* = 0, the electric field shifts the quasi‐equilibrium position of K^+^ ions in the channel toward reservoir 2, with some Cl^−^ ions following them to partially restore electroneutrality, creating an ionic accumulation (depletion) in the channel near (away from) reservoir 2, as shown in Figure 4B. A subsequent Δ*p* applied along the direction of the electric field expels the ionic accumulation from the channel into reservoir 2, diminishing the ionic concentration inside the channel (Figure 4B; Figures S15C and S15D), and thus, reducing *G*. Hence, at Δ*V* > 0, *G*_p_ < 0 and *G*_p_.Δ*V* < 0, which causes *I*_stream_ to reduce with positive Δ*V* (Figure 3D and Figure S14). In contrast, at Δ*V* = −0.4 V, when Δ*p* = 0, the electric field pushes ions in the opposite direction, expelling them into reservoir 1. A subsequently applied Δ*p* toward reservoir 2 pushes those ions back into the channel (Figure 4C; Figures S15E and S15F). At the other end of the channel, the Δ*p* also pushes some ions out into reservoir 2. With the former effect dominating the latter (Figure 4C; Figures S15E and S15F), Δ*p* increases the ionic concentration inside the channel, which elevates G. Hence, at Δ*V* < 0, *G*_p_ > 0 or *G*_p_.Δ*V* < 0. This negative *G*_p_.Δ*V* leads to the decrement of *I*_stream_ with the exertion of negative Δ*V* (Figure 3D and Figure S14). Such analyses of the pressure sensitivity of ionic conductance explains the shape of the experimentally obtained *I*_stream_–*V* curves of Figure 3C to a great extent.

**FIGURE 4:**
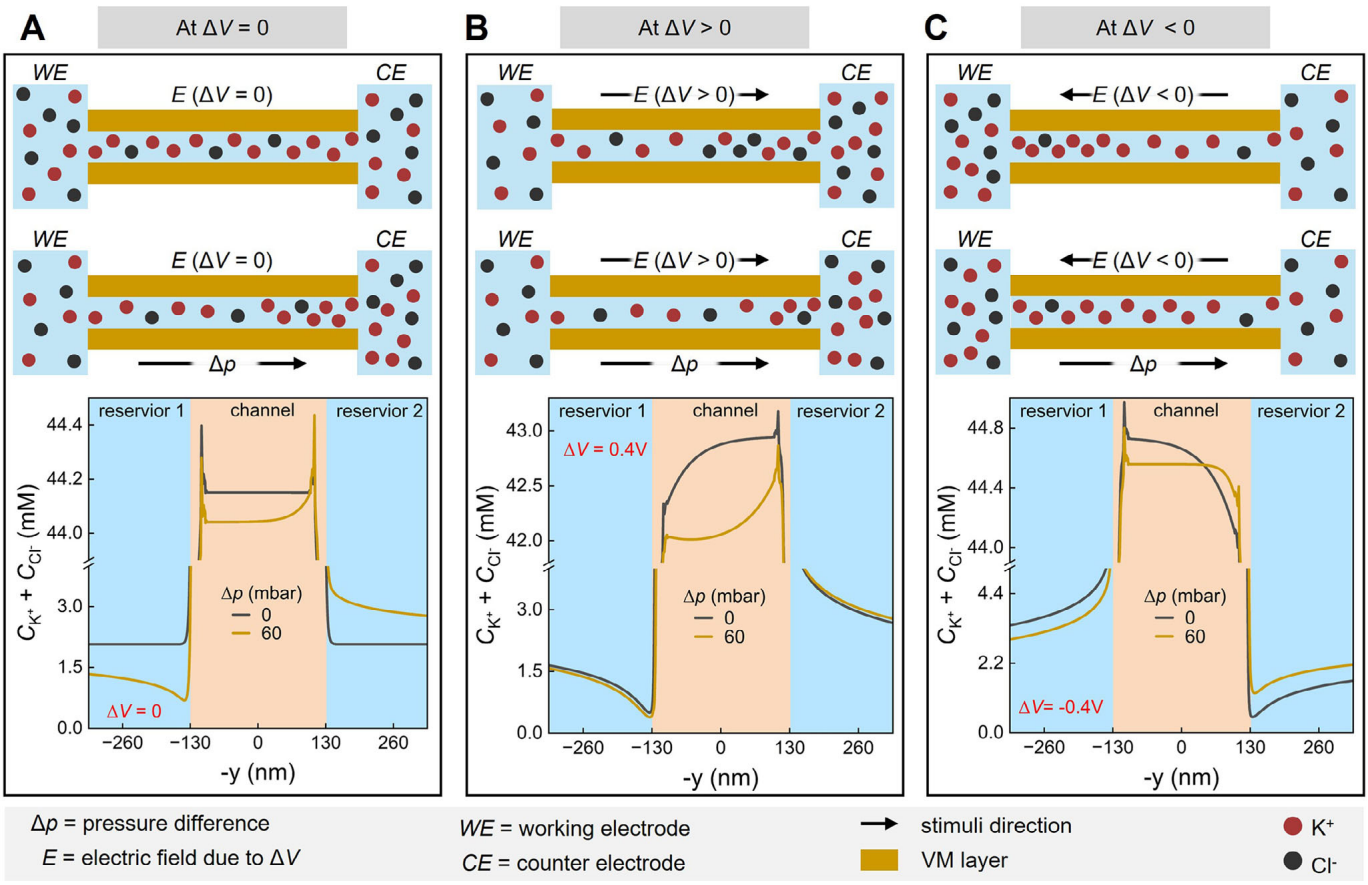
Simulation of factors governing *I_stream_* (Δ*V*, Δ*p*). Schematic illustration of the transport of ions through a VM channel under (A) Δ*V* = 0, (B) Δ*V* > 0 and (C) Δ*V* < 0 with different Δ*p*. The numerically simulated profiles of total ionic concentration $\left(C_{K^{+}}+C_{Cl^{-}}\right)$ on the channel centreline DK (Figure S12) at different *∆p* are shown in (A), (B), and (C) for Δ*V* = 0 mV Δ*V* = +400 mV and Δ*V* = −400 mV, respectively.

However, our simulations cannot explain the initial increase in *I*_stream_ when negative Δ*V* is aplied, as seen in Figure 3C. With the prospect that it arises from the modulation of *θ* with Δ*V*, we calculate *θ* of the VM channels from the experimental data in Figure 3C using the Grahame equation (Figure 5A). At Δ*V* = 0, *θ* is estimated to be −4 mC.m^−2^ from the corresponding *I*_stream._ Increasing Δ*V* beyond 50 mV, however, makes *θ* positive, indicating a reversal in the polarity of the surface charge density of the VM channels (Figure 5A). Conversely, under negative Δ*V*, raising |Δ*V*| initially makes *θ* more negative and then causes |*θ*| to reduce slightly. However, *θ* remains negative across the negative‐Δ*V* range. Numerical simulations also provide the variation of *θ* with Δ*V* that mimics the characteristics of the experimental *I*_stream_‐*V* curve in Figure 3C, which agrees with the *θ* calculated using the Grahame equation (Figure 5A). We further record the corresponding spatially averaged ionic concentrations in the channel (Figure 5B). At Δ*V* = 0, cations are predominately transported, consistent with the cation selectivity of VM channels as *θ* < 0 (Figure 5A). A negative Δ*V* promotes cation‐dominated transport further (Figure 5B) as *θ* becomes more negative (Figure 5A). However, at Δ*V* > 0, anionic flux becomes dominant (Figure 5B) as *θ* > 0 (Figure 5A). Thus, the exact variation of *I*_stream_ with Δ*V* obtained experimentally (in Figure 3C) can be attributed to this stimuli‐induced regulation of *θ*, which redistributes the ionic species across the VM channels, demonstrating the critical role of electrostatic interactions in governing ion selectivity and interfacial transport behaviors.

**FIGURE 5:**
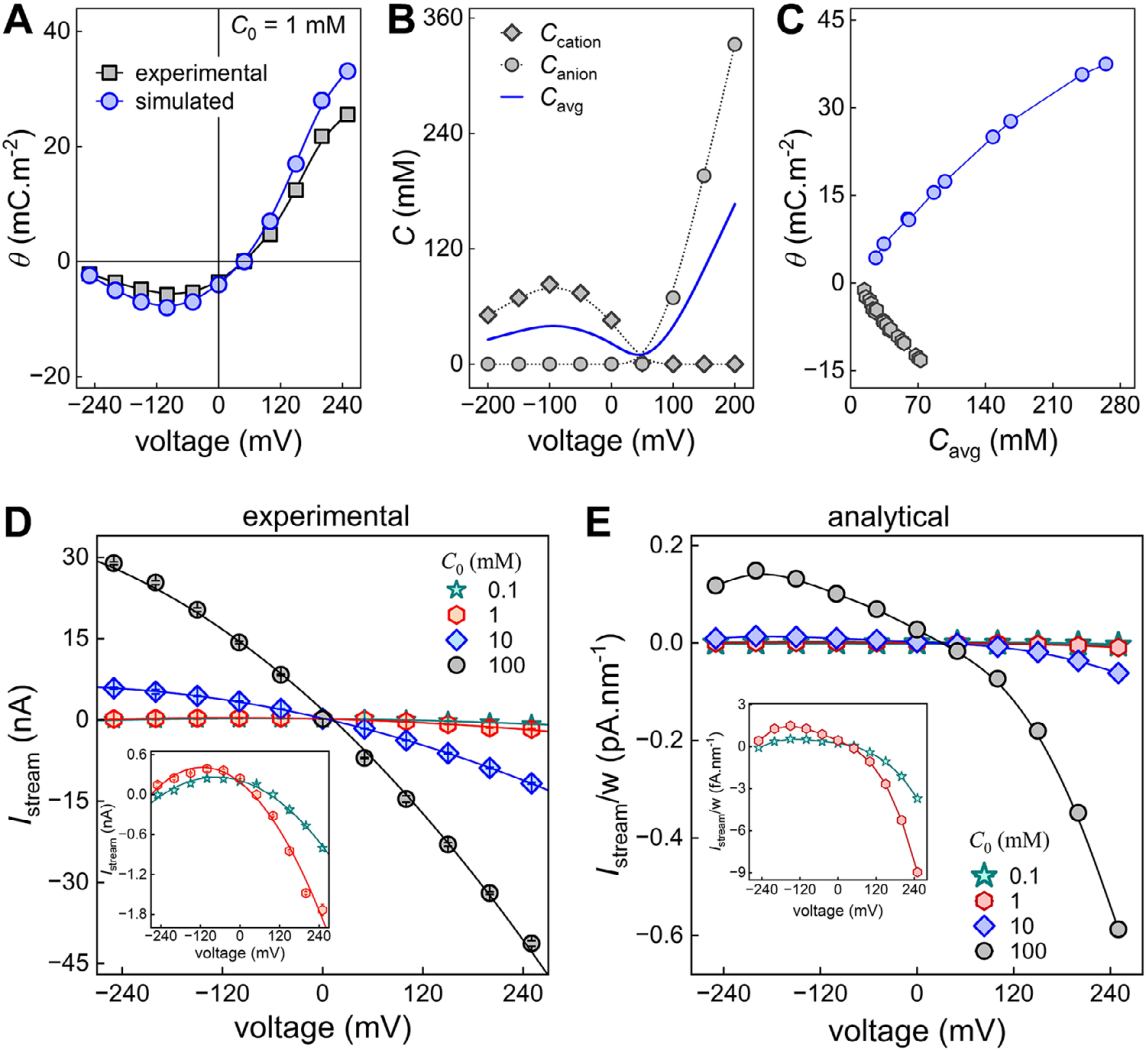
Modified surface charge regulation (*mSCR*) model. (A) Variation of surface charge density (*θ*) with Δ*V* for *C*_0_ = 1 mM KCl: (1) blue circles – obtained by estimating the values of *θ* that replicate the variation of *I_stream_* in the experimental *I_stream_*–*V* curve of Figure 3C for Δ*p =* 500 mbar, through numerical simulations using the system in Figure S12 with *H* = 2 nm and *l* = 0.26 µm, and (2) black squares – derived again from the experimentally obtained *I_stream_*–*V* curve in Figure 3C for Δ*p =* 500 mbar, but using the Grahame equation. (B) Spatially averaged concentration of both ions, {$C_{\mathrm{avg}}=0.5\left(C_{K^{+}}+C_{Cl^{-}}\right)$8, and that of the individual ions across the channel for the simulations in (A). (C) Correlation between the *θ* and *C*_avg_ obtained in (A), (B), and Figure S18 at different Δ*V*, Δ*p* and *C*_0_. (D) *I*_stream_‐*V* curves at *∆p* = 0.5 bar for *C*_0_ = 0.1, 1, 10, and 100 mM KCl. (E) (*I*_stream_/w)–*V* curves obtained analytically using the *mSCR* model in conjunction with our analytically formulated expression of *I*_stream_ (see Section S11, SI) at *∆p* = 60 mbar, *H* = 2 nm, and *l* = 0.26 µm, for different values of *C*_0_. The insets in (D) and (E) focus on the curves for *C*_0_ = 0.1 and 1 mM KCl and in both cases the insets are the same as those in the main plots. Error bars in (D) represent the uncertainty in the measured *I*_stream_ for a particular Δ*p*.

We also performed the numerical simulations for different Δ*p* and reservoir salt concentration (*C*_0_) in Figure S18, which illustrate that *θ* varies with these factors too. So, the quasi‐equilibrium distribution of ions across the channel, and thus, *θ* (which depends on the chemical equilibrium between the channel surface and the ions in its vicinity), vary with Δ*V*, Δ*p*, and *C*_0_. To take this into account, we build a modified surface charge regulation (*mSCR*) model *θ* = *f*(Δ*V*, Δ*p*, *C*_0_), in contrast to the conventional surface charge regulation models [39, 40, 41, 42], that consider *θ* to be a function of *C*_0_​ alone. The *mSCR* model is developed by numerically deriving the variation of *θ* with Δ*V* at different Δ*p* and *C*_0_ that mimic the characteristics of the experimentally obtained *I_stream_*–*V* curves of Figures 3C and 5D, as shown in Figure S18A and Figure 5A. We simultaneously calculate the average ionic concentrations across the channel (*C*_avg_), i.e., 0.5$\left(C_{K^{+}}+C_{Cl^{-}}\right)$, in those conditions, as displayed in Figure S18B and 5B, and correlate them with the corresponding *θ* to extract the relation *θ* = *g*(*C*_avg_), as displayed in Figure S18 and Figure 5C. This *θ*–*C*_avg_ curve, which can be estimated as a linear relation *θ* = (A.*C*_avg_ + B), has two branches (as *θ* can be positive or negative depending on Δ*V*). The curve suggests that |*θ*| rises with *C*_avg_ for the VM channels. Subsequently, we derive the relation *C*_avg_ = *h*(Δ*V*, Δ*p*, *C*_0_). We model *C*_avg_ as |*a*.(Δ*V*)^2^ + *b*.(Δ*V*) + *c*| via the curve‐fitting operations outlined in Figures S18 and S19, and express the coefficients *a*, *b*, and *c* as functions of Δ*p* and *C*_0_​ (Section S10) to obtain an expression of *C*_avg_ in the form of:

(4) $$C_{avg}=\left|\;\left[\alpha .C_{0}+\beta \right]\;.\;\left[{\left(\gamma .\Delta p+\delta \right)}^{2}+\chi \right]\;.\;\left[{\left(\lambda .\Delta V+\nu \right)}^{2}+\tau \right]+\kappa \;\right|$$

where *α*, *β*, *γ*, *δ*, *χ*, *λ*, *ν*, *τ*, and *κ* are constants. Substituting this expression into *θ* = *g*(*C*_avg_) yields the complete *mSCR* relation *θ* = *f* (Δ*V*, Δ*p*, *C*_0_). The derivation of the *mSCR* model is outlined thoroughly in Section S10. For a specific combination of Δ*V*, Δ*p*, and *C*_0_, we obtain a corresponding *C*_avg_, using Equation (4), which yields two different values of *θ via θ* = *g*(*C*_avg_) (Figure 5C). If {*a*.(Δ*V*)^2^ + *b*.(Δ*V*) + *c*} > 0, which occurs toward the negative‐Δ*V* range (compare Figure 5B with Figure S20) where *θ* < 0 (Figure 5A), we select *θ* from the negative branch of *θ* in Figure 5C. Conversely, if {*a*.(Δ*V*)^2^ + *b*.(Δ*V*) + *c*} < 0, we choose *θ* from the positive branch in Figure 5C. Ultimately, for a given set of Δ*V*, Δ*p*, and *C*_0_, we get a specific *θ* from the *mSCR* equation *θ* = *f*(Δ*V*, Δ*p*, *C*_0_). So, the *mSCR* model has a simple physical interpretation: The two physical stimuli of Δ*V* and Δ*p*, alongside the reservoir salt concentration *C*_0_, govern the quasi‐static distribution and concentration of ions within the channel, dictating the chemical equilibrium between the channel surface and the ions, which subsequently determines the surface charge density *θ*. Furthermore, such manifestations of positive and negative surface charge densities in different Δ*V* regimes can be used as a novel route of regulating *θ*, and hence, the ion transport characteristics across Å‐scale channels with a gate voltage. Moreover, the relation *θ* = g(*C*_avg_) is sensitive to variations in the channel height (*H*) considered in the numerical simulations used to build the *mSCR* model. As illustrated in Figure S22, performing these simulations with a higher value of *H* (4 nm) alters the *θ* = g(*C*_avg_) relation slightly; it results in a higher slope of the *θ*‐*C*_avg_ curve and leads to higher |*θ*| at a specific *C*_avg_. However, it does not change the fundamental characteristics of the *θ*‐*C*_avg_ relation. Hence, the consideration of *H* = 2 nm (not 6.3 Å) in the numerical simulations used to build the *mSCR* model for the 6.3‐Å‐thick VM channels potentially overestimates the slope of the *θ*‐*C*_avg_ curve and the magnitude of *θ* at a given *C*_avg_, but should still be able to capture the essential trends in the variation of *θ* with *C*_avg_ within the VM channels. Besides the physical stimuli of Δ*V* and Δ*p*, the chemical stimulus of pH also influences *θ*, as revealed via the results of Figure 2D; in short, a rise in pH makes *θ* more negative and vice‐versa. We integrate this effect of pH into the *mSCR* model using a method outlined in Section S11, via the parameter *c* of the relation *C*_avg_ = *h*(Δ*V*, Δ*p*, *C*_0_) = |*a*(Δ*p*, *C*_0_).Δ*V* ^2^ + *b*(Δ*p*, *C*_0_).Δ*V* + *c*(Δ*p*, *C*_0_)| that was formulated in Section S10 at a specific *pH* (= 6). Substituting this *c* with a “pH‐adjusted *c*” or *c*_adj_ in the *mSCR* equation via the correctional term Δ*c*, where:

(5) $$c_{adj}\left(\Delta p,\;C_{0},\;pH\right)=c\left(\Delta p,\;C_{0}\right)+\Delta c\left(\Delta p,\;C_{0},\;pH\right)$$

helps us take into account the influence of pH into the *mSCR* model by enabling the estimation of *θ* at any pH, but only for a specific combination of Δ*p* and *C*_0_ at a time.

In addition, we test the mathematical relationship in Equation (1) for *C*_0_ = 0.1 mM, which also shows similarities between the derived and the experimental *I*_max_ values across different *∆p* (Figure S9D). *V*_eq_ and *ω* are found to be independent of *∆p* for a particular electrolyte concentration. Additionally, we investigate the *I*_stream_ *‐V* curves for different *C*_0_ (and *∆p*) in Figures S8, S9, and S10, with those for *∆p* = 0.5 bar displayed in Figure 5D. All these curves follow Equation (1), but have different *I*_max_, *ω*, and *V*_eq_ (Figures S11A, S11B, and S11C, respectively). Both *I*_max_ and *V*_eq_ increase, while *ω* decreases, with increasing *C*_0_. Lastly, to validate the analytical capability of our *mSCR* model, we use it with our analytical formulation of streaming current (Section S12) to generate *I*_stream_–*V* curves at different *C*_0_ in Figure 5E. We observe that these curves mimic the characteristics of those obtained experimentally (in Figure 5D) at lower *C*_0_, *i.e*., 0.1 and 1 mM. This also holds true at higher *C*_0_, that is, 10 and 100 mM, except in the high negative‐Δ*V* range. This slight deviation at the higher *C*_0_ arises because we developed the *mSCR* model using the experimentally obtained data points at the lower *C*_0_; we could not use the data points at the higher *C*_0_ due to convergence issues in the numerical simulations (convergence becomes increasingly difficult at larger |*θ*| and we needed to consider extremely high |*θ*| while attempting to use the high‐*C*_0_ data points in developing the *mSCR* model). Thus, the model works better at lower *C*_0_ than at higher *C*_0_, which also signifies the need to use larger sets of data over a wider range of parameters while building the *mSCR* model in order to make it capable of predicting surface charge regulation, and thus, the ion transport characteristics, more accurately across a broader range of operating conditions, channel materials, and ionic (chemical) species. However, except for (only) the monotonous growth of *I*_stream_ with the application of higher magnitudes of negative Δ*V* at high *C*_0_, the *I*_stream_–*V* curves obtained analytically (using the *mSCR* model) in Figure 5E are able to replicate all the other characteristics of the experimentally obtained *I*_stream_–*V* curves in Figure 5D, indicating that the *mSCR* model is still effective in capturing the essential physics governing the multi‐stimuli‐regulated ion transport in the VM channels. The Δ*V* dependent modulation of *I*_stream_ was also observed with different salts as electrolyte. The ionic currents with NaCl, CaCl_2_ and MgCl_2_ also varied at different *∆p* under positive and negative polarity of Δ*V* like KCl, but at different magnitude, Figure S25 using 1 mN of the electrolyte solution.

## 3 Conclusion

In this study, we proposed the use of the naturally occurring confined spaces in layered materials, such as vermiculite (VM), as 2D ion channels with multi‐responsiveness toward pressure, pH, and electric field. The VM Å‐scale channels developed here exhibit cation‐dominated ion transport. Under a constant voltage, applying a pressure drop along the channels changes the magnitude of the (otherwise voltage‐driven) ionic current, thus giving rise to a streaming current (*I*_stream_). *I*_stream_ is observed to depend on both the applied voltage and pressure, along with the ionic strength and pH of the electrolyte, with the latter capable of regulating the ion selectivity of the channels. Subjecting the VM channels to the dual stimuli of voltage and pressure drop modulates *I*_stream_, demonstrating the electromechanical tunability of the ion transport through the channels. We derived a mathematical model of this dual‐stimuli dependency from our experimental data, which provides insight into how the interplay of these stimuli governs *I*_stream_. Finite‐element simulations later indicated that the electromechanical coupling of the ion transport here stems from (i) the deformation of the voltage‐governed quasi‐equilibrium distribution of ions across the channel by an applied pressure via hydrodynamic flow, which alters the ionic conductance, and (ii) the modulation of the channel's surface charge density by not only the reservoir salt concentration, but also the applied voltage and pressure. To formulate the latter, we built a “modified” surface charge regulation (*mSCR*) theoretical model to address the shortcomings of past *SCR* frameworks that considered surface charge density to be dependent only on the reservoir salt concentration, besides using it to elucidate the multi‐stimuli‐regulated ion transport demonstrated here. Our findings, therefore, indicate natural clay‐based channels as promising candidates for biomimetic ionic flow platforms, enabling the development of smart nanofluidic devices with tunable ionic selectivity and gating behavior for future applications in environmental sensing, energy harvesting, and bioelectronics.

## Author Contributions

B.R. supervised the project. B.R. and R.K.G. designed and directed the project. R.K.G. performed all the experiments and analysed the data contained in this paper. R.K.G. H.J. and A.B. prepared the VM flakes. A.B. and H.J. fabricated VM devices. H.J. provided STEM image. A.P. and N.A. carried out the numerical simulations and theoretical modelling and provided all theory data. R.K.G. and B.R. wrote the manuscript with inputs from A.P. and N.A. All the authors contributed to discussions.

## Conflicts of Interest

The authors declare no conflicts of interest.

## Supporting information




**Supporting File**: adma74804‐sup‐0001‐SuppMat.pdf.

## Data Availability

The data that support the findings of this study are available from https://figshare.com/s/dce33c70e09e85c95bad.
