# Supplementary material for: Multi‐Stimuli Responsive Angstrom‐Scale Two‐Dimensional Channels of Natural Layered Materials
Source: Adv Mater. 2026 Aug 26;38(59):e74804. doi: 10.1002/adma.74804 (PMC13689888; doi:10.1002/adma.74804)
Supplement: Supplementary file 1 [file ADMA-38-e74804-s001.pdf]

Supporting information for

Multi-Stimuli-Responsive Angstrom-scale Two-dimensional Channels of Natural Layered Materials

Raj Kumar Gogoi[1,2], Arghyadeep Paul[3], Ankit Bhardwaj[1,2,#], Hiran Jyothilal[1,2,#], Narayana R. Aluru[4,*], Boya Radha[1,2,5,*]

[1] Department of Physics & Astronomy, School of Natural Sciences, University of Manchester, Manchester, UK
[2] National Graphene Institute, The University of Manchester, Manchester, UK
[3] Department of Mechanical Science and Engineering, Beckman Institute for Advanced Science and Technology, University of Illinois at Urbana−Champaign, Urbana, Illinois 61801, USA
[4] Walker Department of Mechanical Engineering, The University of Texas at Austin, Austin, Texas 78712, USA
[5] Photon Science Institute, The University of Manchester, Manchester, UK

[#]Equally contributed
*Correspondence to be addressed to: radha.boya@manchester.ac.uk and aluru@utexas.edu

S1. Preparation and characterization of vermiculite (VM) flakes

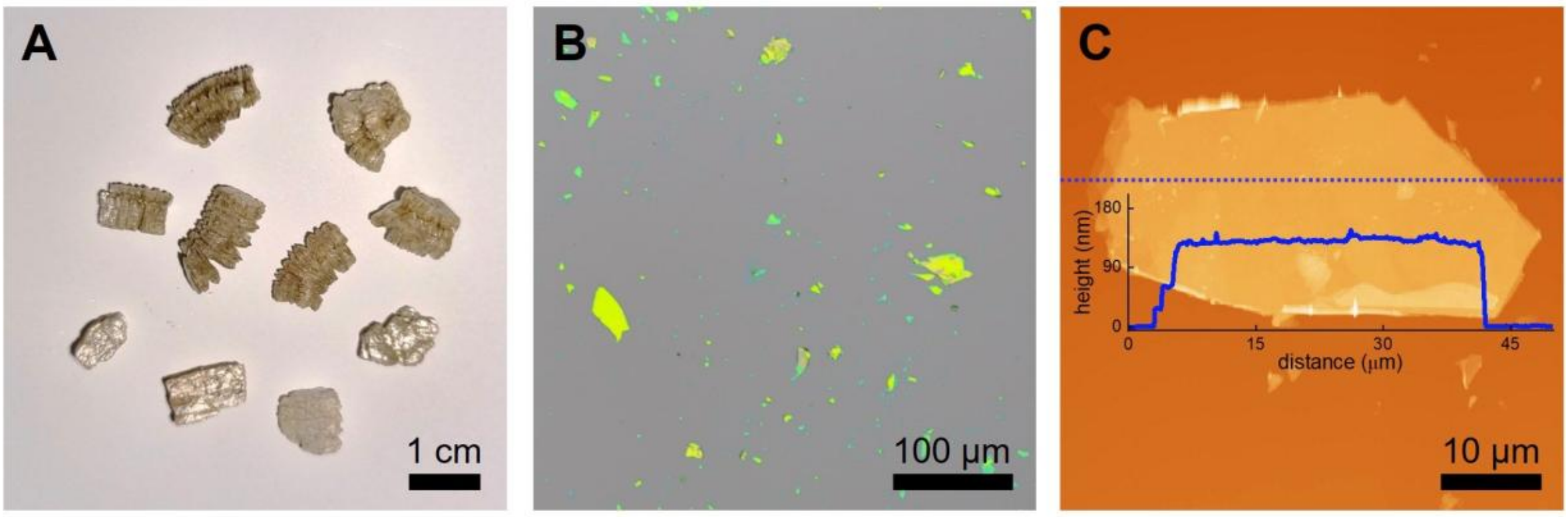

Figure S1. Vermiculite (VM) flakes. (**A**) Digital photos of VM crystals. (**B**) Microscopic image of as prepared VM flakes transferred on top of a SiO_2 surface. (**C**) Representative AFM image (with corresponding height profile) of the VM flake used to fabricate the VM channel device.

S1.1. Preparation of VM flakes

The VM flakes were prepared by mechanically peeling VM crystals (Figure S1A) using residue free tape. The exfoliated flakes were then transferred onto a Si/SiO_2 substrate (with a SiO_2 thickness of 290 nm) by mechanically pressing the tape containing the flakes onto the SiO_2 surface (Figure S1B). Uniform, flat flakes with sharp edges were selected for device fabrication. Figure S1C shows representative atomic force microscopy (AFM) of a VM flake with the corresponding height profile.

S1.2. Atomic force microscopy (AFM) of the edge of a VM flake

AFM was performed to further characterize the layered structure of exfoliated VM flakes. To estimate the interlayer spacing under ambient conditions, a thin VM flake was selected and the step heights at the flake edge were analysed. The corresponding AFM topography image is shown in Figure 1D. Height profiles extracted from different regions of the flake edge revealed step heights in the range of ~14 to 17 Å, close to the $d_{spacing}$ of ~14.3 Å obtained from XRD measurements in Figure 1C. A close correlation between the AFM and XRD results is expected, as both measurements were performed under ambient conditions, where the interlayer spaces of VM remain hydrated.

S1.3. Cross-section TEM imaging

The sample for the HAADF-STEM was prepared from VM flakes using a site-specific in situ lift-out method. This cross-section lamella fabrication was carried out in a Zeiss Crossbeam 540 dual-beam SEM/FIB instrument, with focused ion beam (FIB) milling performed perpendicular to the VM layers. To protect the region of interest from ion-induced damage, thin amorphous carbon and platinum coatings were first deposited by electron-beam-assisted deposition, followed by the deposition of a thicker platinum protective layer using ion-beam deposition. The selected region was progressively milled to form a thin lamella. The lamella was subsequently attached to an OmniProbe micromanipulator through ion-beam-induced platinum deposition and released from the bulk material by undercut milling. After lift-out, the specimen was mounted onto a TEM half-grid and further thinned to a final thickness below 50 nm. Final polishing was conducted using sequential low-energy ion milling steps at 5 kV/10 pA and 2 kV/10 pA to minimise surface amorphisation and produce electron-transparent areas suitable for STEM analysis. High-resolution HAADF-STEM imaging was performed under high vacuum condition (~10^{-5} to 10^{-6} Pa) using an aberration-corrected FEI Titan G2 80–200 ChemiSTEM microscope. Images were acquired using a convergence semi-angle of 21 mrad, a HAADF detector inner collection angle of 48 mrad, and a probe current of 60 pA. From the layered structure observed in the cross-sectional HAADF-STEM image, the interlayer spacing of the VM flake was

measured to be ~8 to 1.1 Å (Figure 1E), which is smaller than the value estimated from the XRD analysis shown in Figure 1C.

The discrepancy in the interlayer spacing arises from differences in the hydration state of the material under the respective experimental conditions.[1] The XRD measurements were performed under ambient conditions, where water molecules are retained within the interlayer galleries. The observed $d_{spacing}$ of ~14.3 Å supports the presence of almost two layers of intercalated water molecules.[2] In contrast, HAADF-STEM imaging was conducted under high-vacuum conditions, which promotes dehydration of the vermiculite interlayers. The removal of interlayer water molecules results in a reduced interlayer spacing, leading to the smaller values observed in the HAADF-STEM measurements.

S1.4. Estimation of channel height (*H*) of the VM channels

The channel height (*H*), in our VM devices is defined as the free space between two adjacent VM layers. It was estimated from the interlayer spacing of the VM layers after subtracting the thickness of a single VM layer. The interlayer spacing was determined to be ~14.3 Å from XRD analysis (Figure 1C), ~14 to 17 Å from AFM step-height analysis (Figure 1D) under ambient conditions, and ~8 to 11 Å from STEM analysis under dehydrated conditions (Figure 1E). Because XRD provides an average interlayer spacing and gives a value similar with the AFM measurements both under ambient conditions, we used the XRD-derived value of ~14.3 Å as the representative interlayer spacing. The thickness of a single VM layer is approximately 8 Å, as reported in the literature[1, 3], and this value is also similar with the layer-to-layer distance observed in the STEM image under dehydrated conditions (Figure 1E). Therefore, using these approximations, *H* was estimated to be ~6.3 Å.

S2. Fabrication of the two-dimensional (2D) channels devices of vermiculite

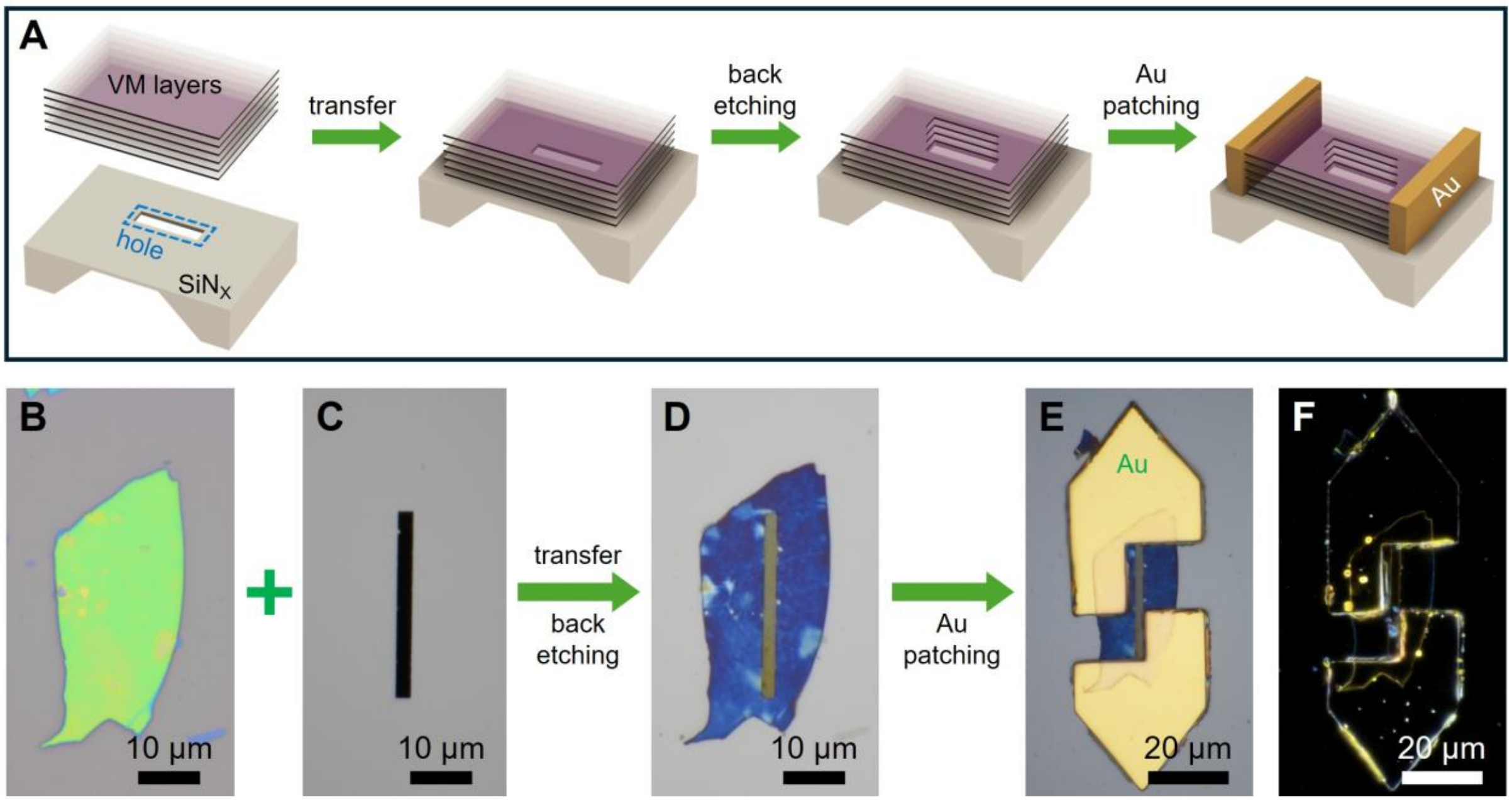

Figure S2. Fabrication of VM 2D channel device. (**A**) Schematic illustration of the fabrication process of the VM channels (not to scale). Microscopic images (*top-view*) showing (**B**) VM flakes, (**C**) blank micro-hole, (**D**) VM flake on top of the micro-hole, followed by back etching, (**E**) bright field and (**F**) dark field images of the VM device. Gold is deposited on the desired places of the VM flake in (**E**) for defining the channels' dimension and to improve the adhesion with the bottom substrate.

These 2D channel devices were fabricated from VM flakes, prepared by mechanically exfoliating the bulk crystal (Figure S1A), followed by transferring of the desired flakes (~80 nm to 250 nm thick) on top of micro holes, completely sealing the holes *via* a wet transfer method[4] (steps are shown in

FigureS2). Subsequently, the flakes were etched from the hole side (to a desired thickness) to take advantage of the natural free space between the layers, with the well-defined layered structure of the VM crystals (FigureS1A inset) providing 2D channels with a height (*H*) ~6.3 Å. To facilitate the confined spaces as 2D channels for mass transport, the VM flake from the micro hole side is plasma etched (using BCl_3 gas) to a desired thickness (T_{etch}), which determined the number of exposed channels (*N*) as: $N = 2\frac{T_{\text{etch}}}{d_{\text{spaching}}}$.

Etching of VM: An inductively coupled plasma (ICP) etching system (Plasmalab 100 ICP-65, Oxford Instruments) was used to the etching of the VM flakes. The etching process utilized a gas mixture of either argon (Ar) /trifluoromethane (CHF_3) /chlorine (Cl_2) or Ar/CHF_3/boron trichloride (BCl_3). The etch rate was determined by measuring the flake height before and after etching and divided by its exposure time. A plot of the etch thickness vs exposure time is shown in FigureS3. The average etch rate achieved was 1 nm/s. VM devices with different *N* were fabricated considering the etch rate of 1 nm/s.

Gold deposition: A 50 nm layer of gold with a 5 nm chromium layer (for adhesion) was deposited at the specific locations on the VM devices (Figure S2E) via thermal evaporation. The entire device was then baked on a hot plate at 250 °C in open atmospheric condition to improve the adhesion. This deposition process has *two distinct advantages*: *firm adhesion of the VM flake on top of the micro-hole* and *helps in defining the channels pathway*.

S3. Ion transport measurements

The ion transport measurements were performed using custom-made electrochemical set-up (shown in Figure 1A as schematic diagram). Each reservoir is made from PEEK (polyether ether ketone). The VM channels on top of the SiN_X chip were placed between the two reservoirs, using O-rings between each unit to ensure leak-free sealing. For all the ion transport measurements, 2 mL of electrolytes were used in all the experiments, concentration dependent on the intended experiment. Ag/AgCl electrodes were immersed in each of the reservoirs and the electrochemical measurements were recorded with *Keithley* 2636B SourceMeter using LabVIEW software. Prior to measurement with electrolytes, the VM channels in the electrochemical setup were washed with deionized (DI) water for several times to ensure cleanliness. The pH of the KCl solutions was ~6 unless otherwise specified.

S4. Theoretical ionic conductance of VM devices

The ionic conductivity (σ) relates to the conductance (*G*) of a 2D channel as:

$$\sigma = G\frac{l}{w \times H} \qquad \text{S1}$$

where *l*, *w* and *H* are the length, width and height of the channel, respectively.

The theoretical *G* (G_{theory}) of a VM device having *N* numbers of channels is calculated from the theoretical σ (σ_{theory}) as:

$$G_{\text{theory}} = \frac{N \times w \times H}{l} \times \sigma_{theory} \qquad \text{S2}$$

The experimental *G (= I/V)* of the VM channels devices for different electrolytes was estimated from the slope of the *IV* curves measured across the VM channels.

S5. Electrochemical characterization of VM 2D channels

We measure the ionic flows through the channels by mounting the VM device between two electrolyte reservoirs. A Keithley 2636B source meter was used to record the electrochemical characteristics, with custom-made Ag/AgCl electrodes immersed in the reservoirs (Figure 1A). The voltage-dependent-current (*IV*) of the VM channels, recorded with 1 M KCl as electrolyte in both the chambers, gave a

linear *IV* curve (Figure S3A, black curve) with a finite conductance ($G = I/V$). To validate if the ion transport only occurs through the VM channels, we performed control experiments: by measuring the *IV*s through a micro hole (Figure S3A, red curve) with a VM flake on top, without any etching to expose VM channels (Figure S3A, green curve). As expected, the $G_{hole} >> G_{VM} >> G_{flake}$ (~ 0 S) for 1 M KCl, confirming that the observed ion currents in VM devices are due to the flow of ions through the interlayer channels. Further, we measured ion conductance G (at 1M KCl) of several VM devices with etching depth T_{etch} of 0 nm (control), 40 nm (*device #3*), 60 nm (*device #1* and *#5*), 70 nm (*device #4*), 75 (*device #2*) and 150 nm (*device #6*), which showed that the G linearly increased with the increase in T_{etch}, (*i.e.*, N), Figure S3B. To verify the KCl concentration (C) dependent ion transport characteristics of the VM channels, we measured G with similar C in the two reservoirs, which yielded linear *IV* curves (10^{-1} M and 10^{-2} M curves in Figure S3C). The $G \alpha C$ for $C \geq 10^{-2}$ M KCl was observed, and G begins to deviate from the linear dependence for $C < 10^{-2}$ M and saturates after 10^{-3} M KCl, which is an order of magnitude higher than the water G (G_{water}) of the device (Figure S3D). Similar trends of the G *vs* C were observed with several VM devices (Figure S4). The plateau in conductance at low concentrations is typical for nanofluidic channels, as G at lower C is dominated by the surface charge density rather than the C of electrolytes in the reservoirs.

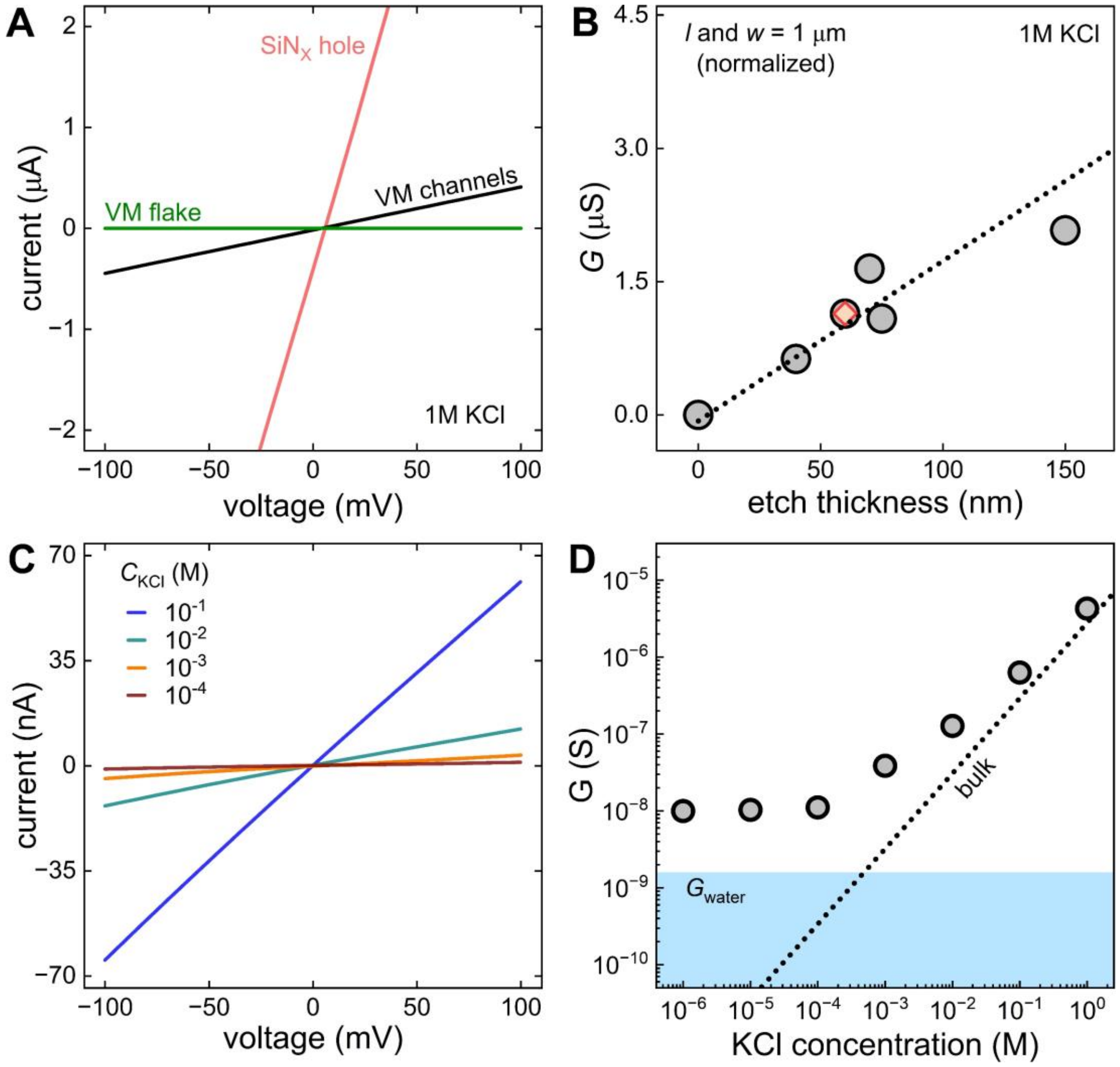

Figure S3. Ion transport through VM fluidic devices. (**A**) Comparison to the current versus voltage (*IV*) curves measured through a SiN_X micro hole (red curve), a VM flake on top of the SiN_X micro hole (green curve), (*i.e.*, no exposed VM channels, green curve) and VM fluidic device (black curve) using 1M KCl as electrolyte. (**B**) Conductance (G) of VM devices with different etch thickness (0 nm (control), 40 nm (*device #3*), 60 nm (*device #1* and *#5*), 70 nm (*device #4*), 75 (*device #2*) and 150 nm (*device #6*)). The l and w of the devices are normalized to 1 µm and the electrolyte is 1M KCl. Inset compares the *IV* curves through a VM device (device

#2) using different concentrations of KCl (**C**) Inset compares the *IV* curves through the VM device using different concentrations of KCl (**D**) *G* vs KCl concentration of a VM fluidic device (device #2). The bulk line is the estimated *G* and G_{water} is the measured water conductance of a device.

Table S1. Details of the devices used.

Device number	Length *l* (µm)	Width *w* (µm)	Height *H* (Å)	T_{etch} (nm)	No. of channels (*N*)
Device #1	7	16	6.3	60	84
Device #2	4.7	18.5		75	106
Device #3	6	15		40	56
Device #4	7	16		70	98
Device #5	8	12		60	84
Device #6	6.7	24		150	210
Device #7	8.5	15.9		60	84
Device #8	8.5	14.5		100	140
Device #9	9	18		15	22

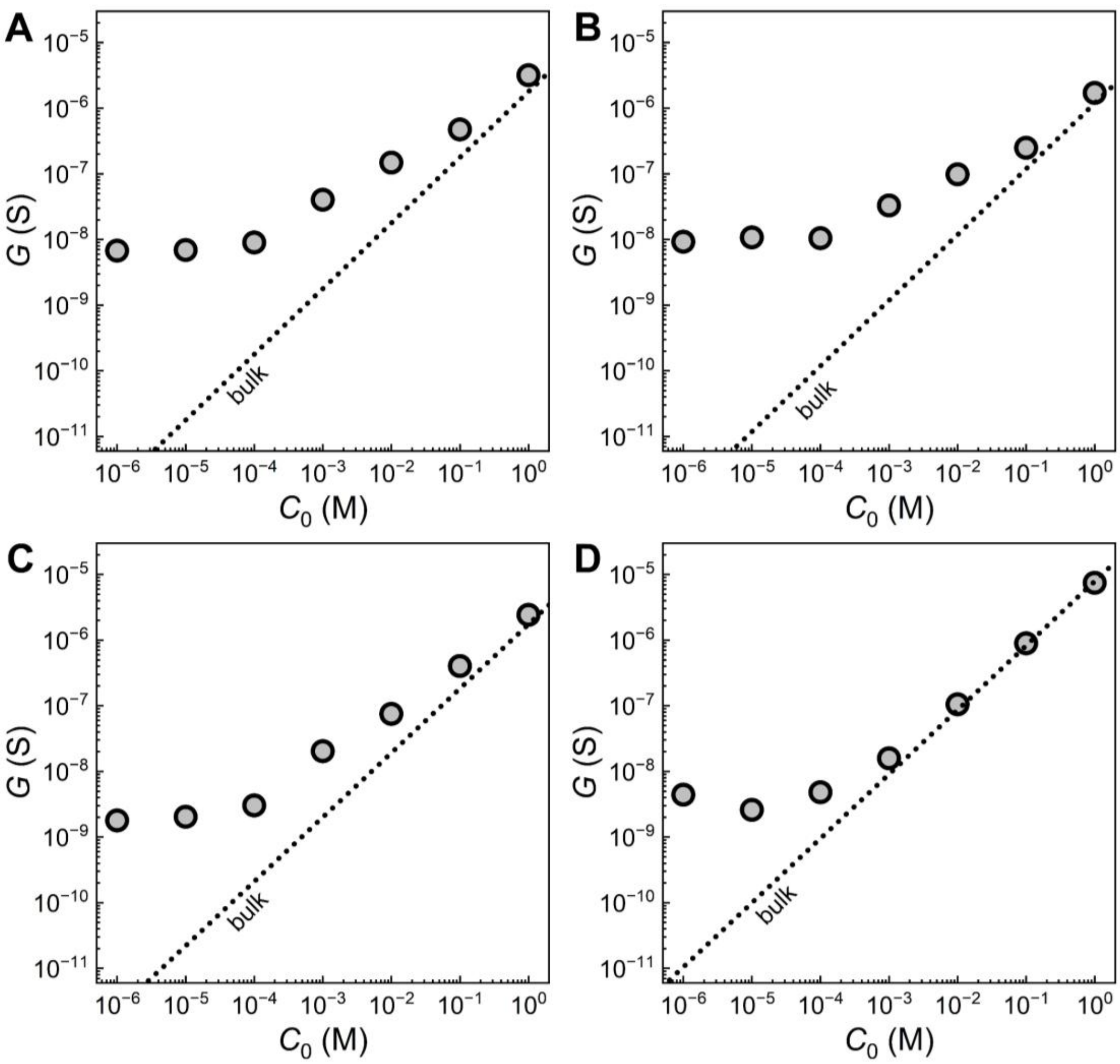

Figure S4. Comparison of conductance (*G*) *vs* KCl concentrations in the reservoirs (C_0) of different VM devices: (**A**) *device #4*, (**B**) *device #5*, (**C**) *device #1* and (**D**) *device #6.*

S6. Estimation of transference number

The transference number, t is calculated from the membrane potential (E_M) as:[5]

$$E_{\mathrm{M}} = (t_+ - t_-)\frac{RT}{nF}ln\left(\frac{\gamma_{\mathrm{feed}}C_{\mathrm{feed}}}{\gamma_{\mathrm{permeate}}C_{\mathrm{permeate}}}\right) \quad \text{S3}$$

Where, F is the Faraday constant; R is the universal gas constant; T is the temperature; n is the number of electrons involved in the reaction; $\gamma_{\mathrm{feed/permeate}}$ and $C_{\mathrm{feed/permeate}}$ are the mean activity coefficient of electrolytes and corresponding concentration in the feed/permeate reservoirs, respectively (generally, we maintain $C_{\mathrm{feed}} > C_{\mathrm{permeate}}$). The E_M is calculated from the total potential (E_T) and the electrode potential (also called redox potential, E_R) as:

$$E_M = E_T - E_R \quad \text{S4}$$

To estimate the E_T, drift-diffusion experiment is performed; where the feed and the permeate reservoirs are filled with 2 mL electrolytes of different concentrations ($C_{\mathrm{feed}} > C_{\mathrm{permeate}}$ in our case). The *IV* curves at this condition have non-zero intercept (Figure S5A) and the voltage with respect to current, $I = 0$ is the E_T. As we are using Ag/AgCl electrodes, the electrode contribution (E_R) needs to be subtracted from the E_T. The E_R of the electrodes for different salts are given by: [5]

$$E_{\mathrm{redox}} = \frac{RT}{nF}ln\left(\frac{\gamma_{\mathrm{feed}}C_{\mathrm{feed}}}{\gamma_{\mathrm{permeate}}C_{\mathrm{permeate}}}\right) \quad \text{S5}$$

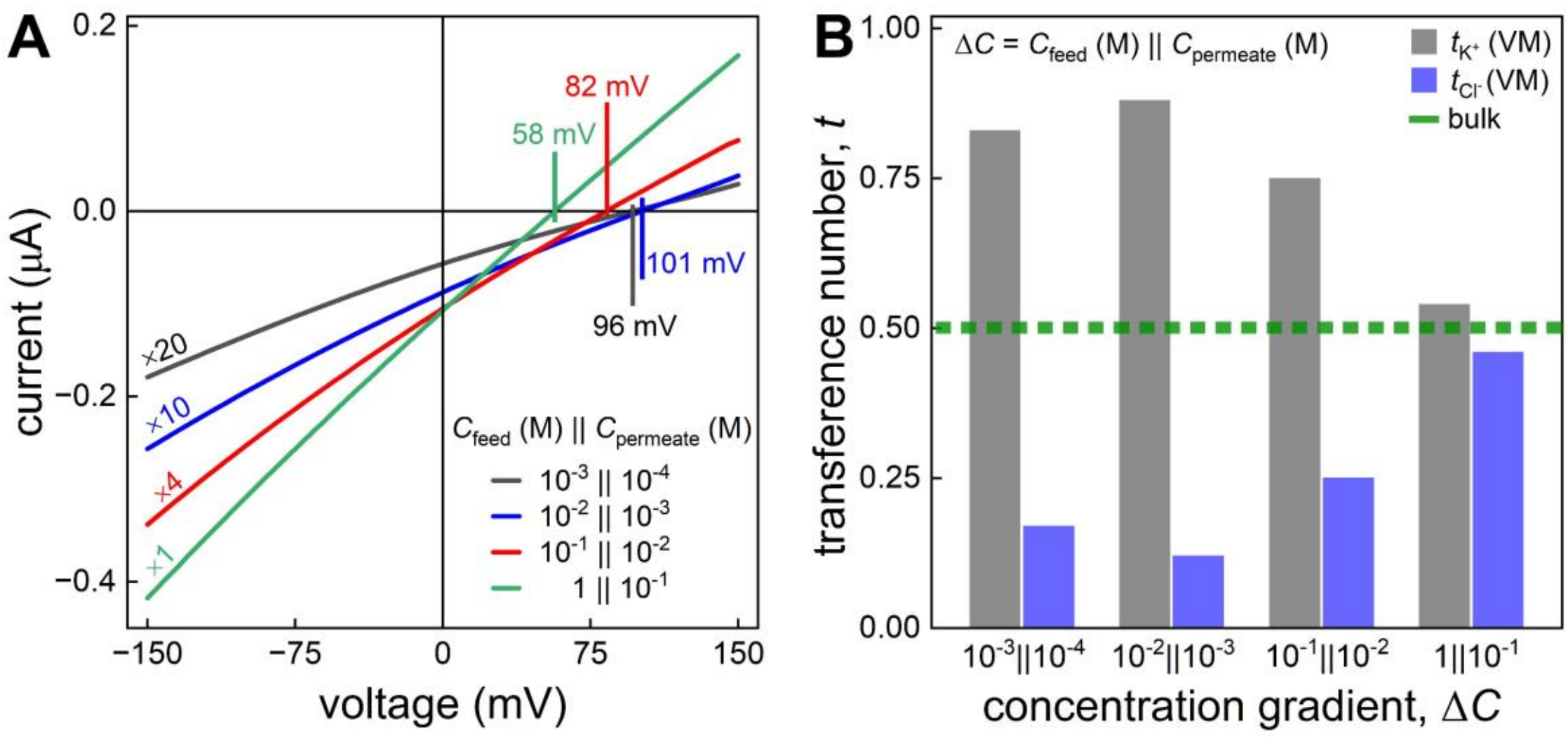

Figure S5. Transference number, *t*. Comparison of (**A**) *IV* curves and (**B**) transference number of VM channels devices under 10-fold concentration gradients for different C_{KCl}. The graphs in (**A**) have the redox contribution of the Ag/AgCl electrodes. *Device #2* is used in this figure. Reservoir 2 (represented in Figure 1A) has been used as the feed reservoir.

With a concentration difference across the reservoirs, *IV* curves with non-zero intercepts were observed (Figure S5A). This is an indication of selectivity of the channels to specific charged ions over others. From the V at $I = 0$ A, *i.e.,* total potential E_T, the transference number (t) has been estimated (after removing the electrode contributions, E_R), which specifies the ion selectivity and the quantity of currents carried by the cationic and anionic species (described in detail in the SI). The E_T under 10-fold concentration gradients with varying C_{KCl} are presented in Figure S5A for VM device. The obtained t_{K^+} values for the VM channels are higher than the bulk values across all the concentrations measured (Figure S5B). However, the magnitude of t_{K^+} varied with concentration, as higher concentrations result in lower t_{K^+} indicating the role of surface-charges in the selective ion transport.

S7. Streaming current

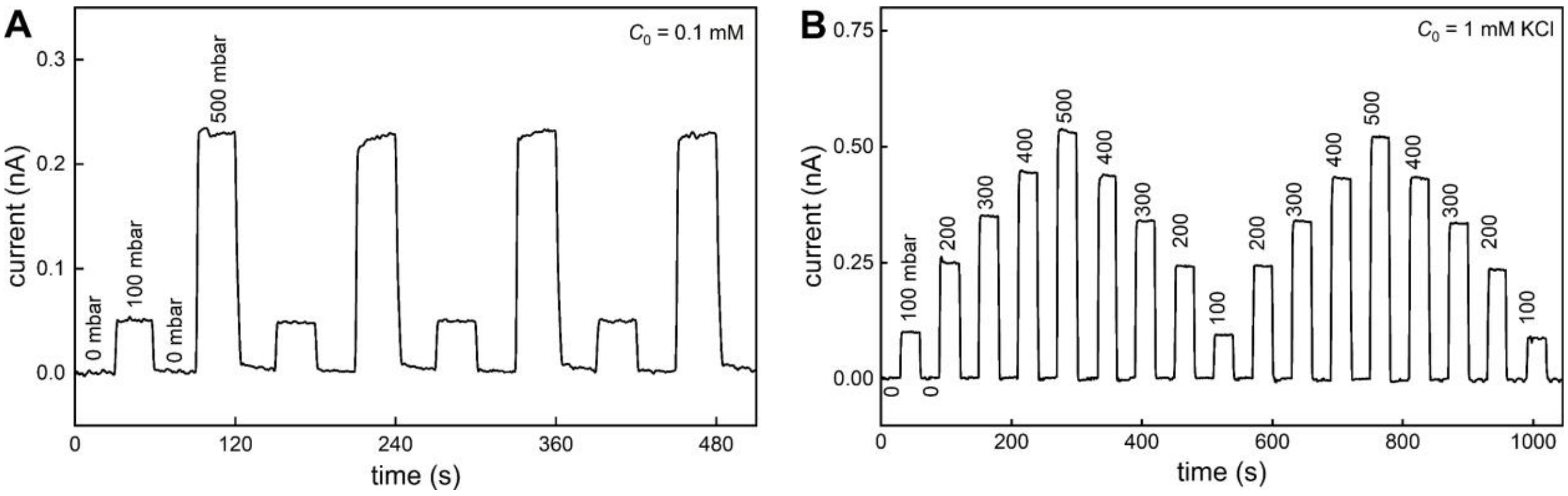

Figure S6. Streaming current stability. The variation of ionic current as a function of time at different Δp across the VM channels devices: (**A**) *device #4* with 0.1 mM KCl and (**B**) *device* #8 is with 1 mM KCl.

The streaming current of the VM channels devices are measured by applying pressure difference across the two sides of the channels. The pressure is applied from the direction of the working electrode. The stability of the streaming current (recorded as ionic current versus time) under different pressure differences (Δp) is shown in Figure S6. The applied pressure was varied sequentially, with each pressure maintained for 30 s. Two pressure pulse sequences were employed: (i) 0 → 100 → 0 → 500 mbar (Figure S6A), and (ii) 0 → Δp → 0 mbar, where Δp was increased from 100 to 500 mbar in increments of 100 mbar and subsequently decreased from 500 to 100 mbar using the same step size (Figure S6B).

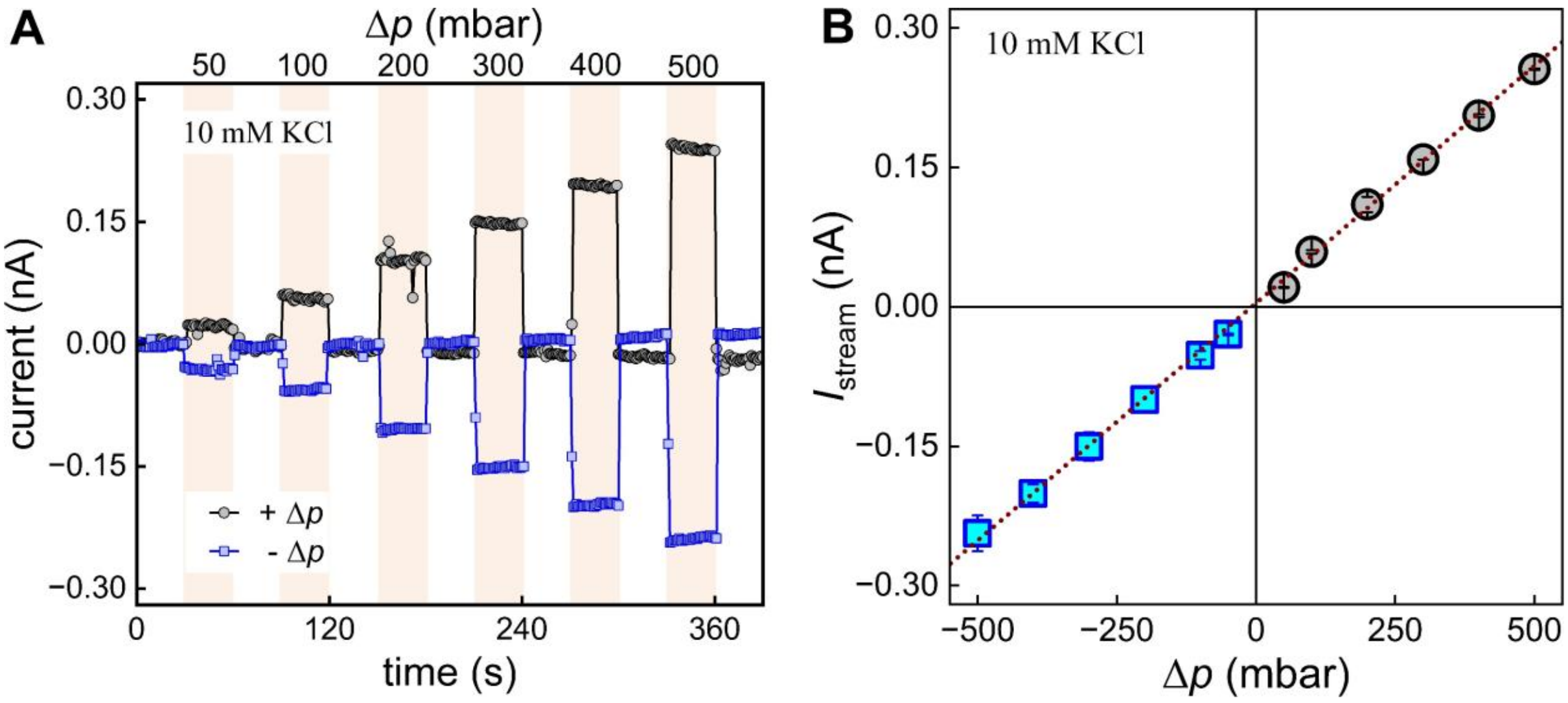

Figure S7. Effect of electrode position on I_{stream}. Comparison of changes in (A) ionic current and (B) I_{stream} at different Δp. The positive direction corresponds to placing the working electrode in the reservoir where pressure is applied, while the negative direction corresponds to placing the counter electrode in this reservoir. The electrolyte used in this experiment is 10 mM KCl (2 mL in each reservoir). *Device #2* is used in this figure.

The position of the electrodes during pressure-driven current measurements determines the polarity of the current. When the working electrode (WE) is placed in the reservoir where pressure is applied (schematic of the experimental setup is in Figure 1A), the ionic current flows in the positive direction (referred to as the positive direction). Conversely, placing the counter electrode in this position reverses the ionic current, resulting in a negative direction (referred to as the negative direction). However, the electrode position did not affect the magnitude of the I_{stream}. The changes in the ionic current and I_{stream} at different Δp are compared in Figures S7A and S7B, respectively.

S8. Electric field dependent streaming

The electric field dependent I_{stream} was measured by applying a fixed electric field followed by applying pressure difference across the two sides of the VM device.

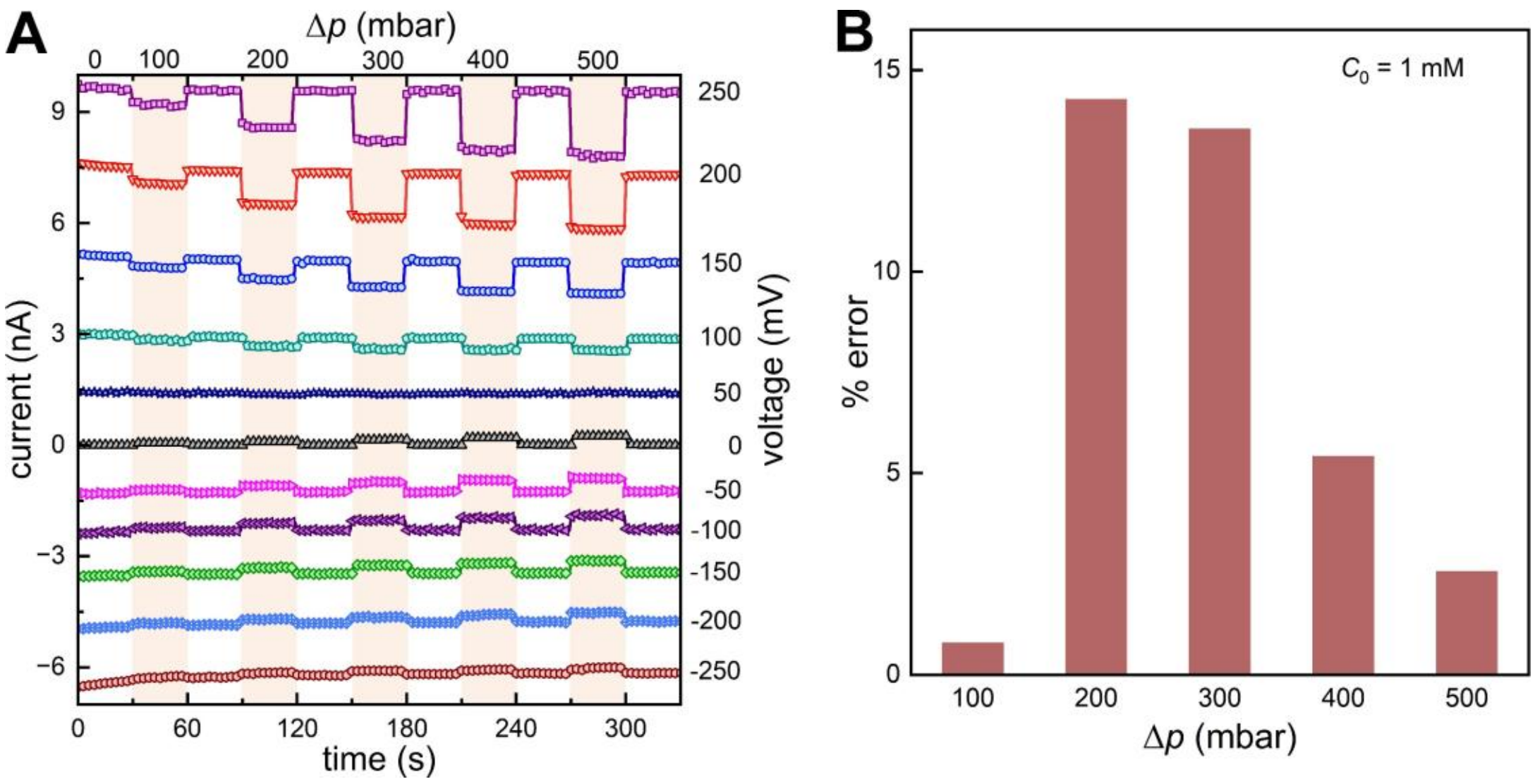

Figure S8. (**A**) Electric field-dependent ionic current *vs* time at different applied pressure with 1 mM KCl as electrolyte. (**B**) Validation of mathematical relation (equation 1) with experimental data as error % between the experiment and derived I_{max}. *Device #4* is used in this figure.

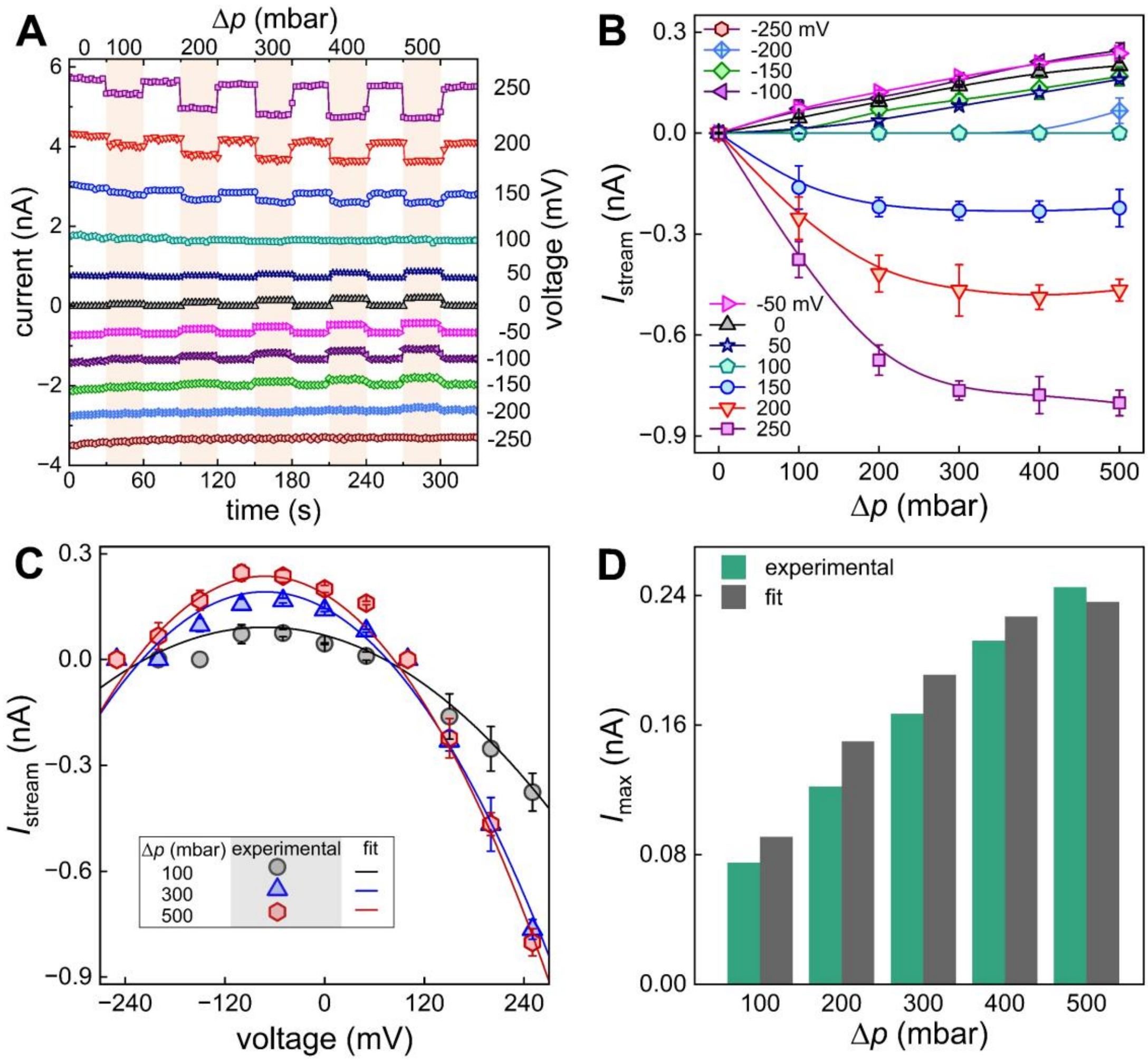

Figure S9. Electric field dependent I_{stream} with 0.1 mM KCl as electrolyte. Electric field-dependent (**A**) ionic current *vs* time and (**B**) I_{stream} at different applied pressures. (**C**) Comparison of I_{stream} at different electric fields with Δp = 100, 300 and 500 mbar. (**C**) Comparison of the experimental and derived I_{max}, obtained by fitting equation. *Device #4* is used in this figure. Error bars in (**C**) represent the uncertainty in the measured I_{stream} for a particular Δp.

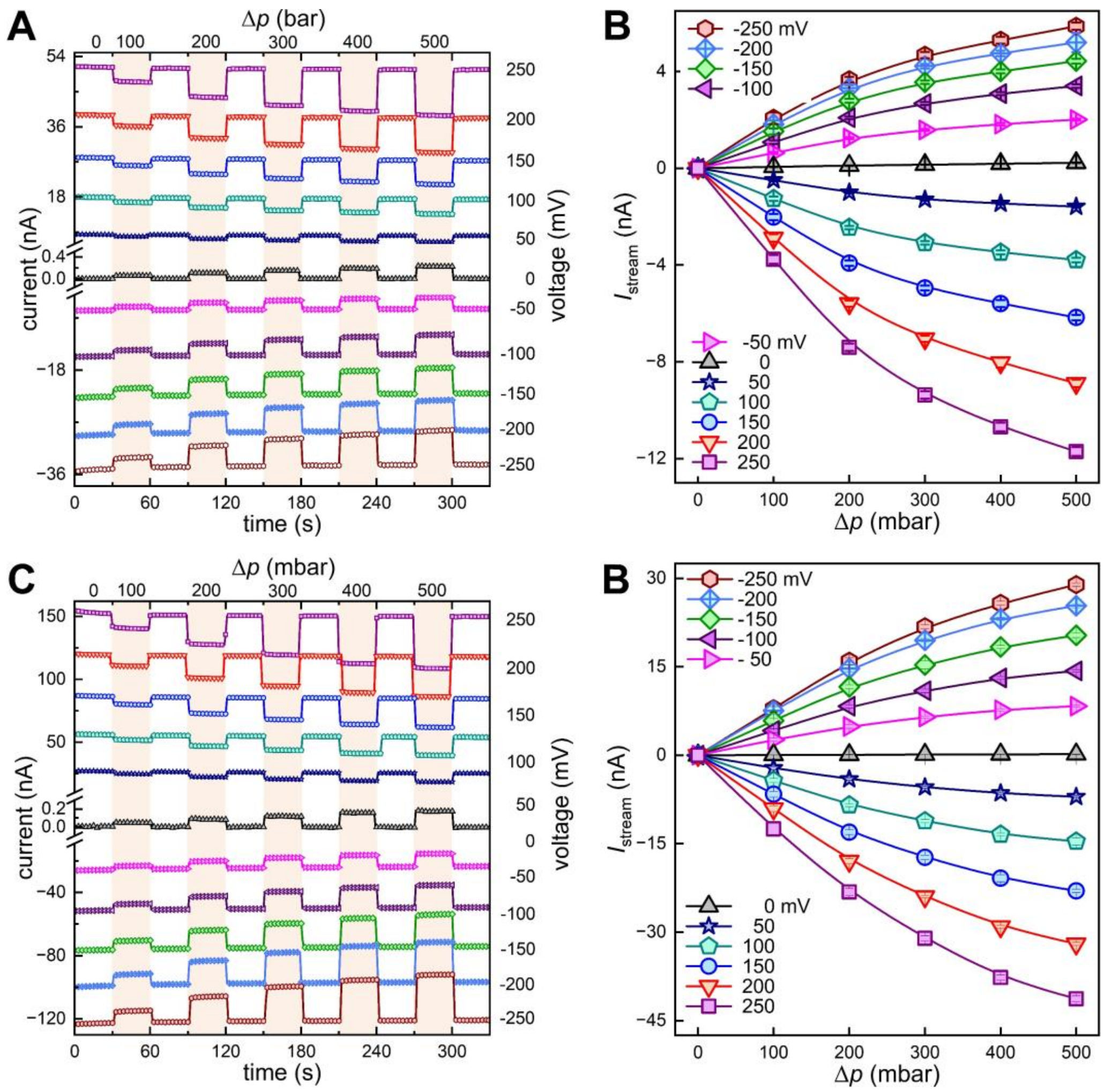

Figure S10. Electric field dependent I_{stream}. Electric field-dependent ionic current *vs* time with electrolytes, C_{KCl} (**A**) 10 mM and (**C**) 100 mM. I_{stream} at different applied pressures with electrolytes, C_{KCl} (**B**) 10 mM and (**D**) 100 mM. *Device #4* is used in this figure. Error bars in (**B**) and (**D**) represent the uncertainty in the measured I_{stream} for a particular Δp.

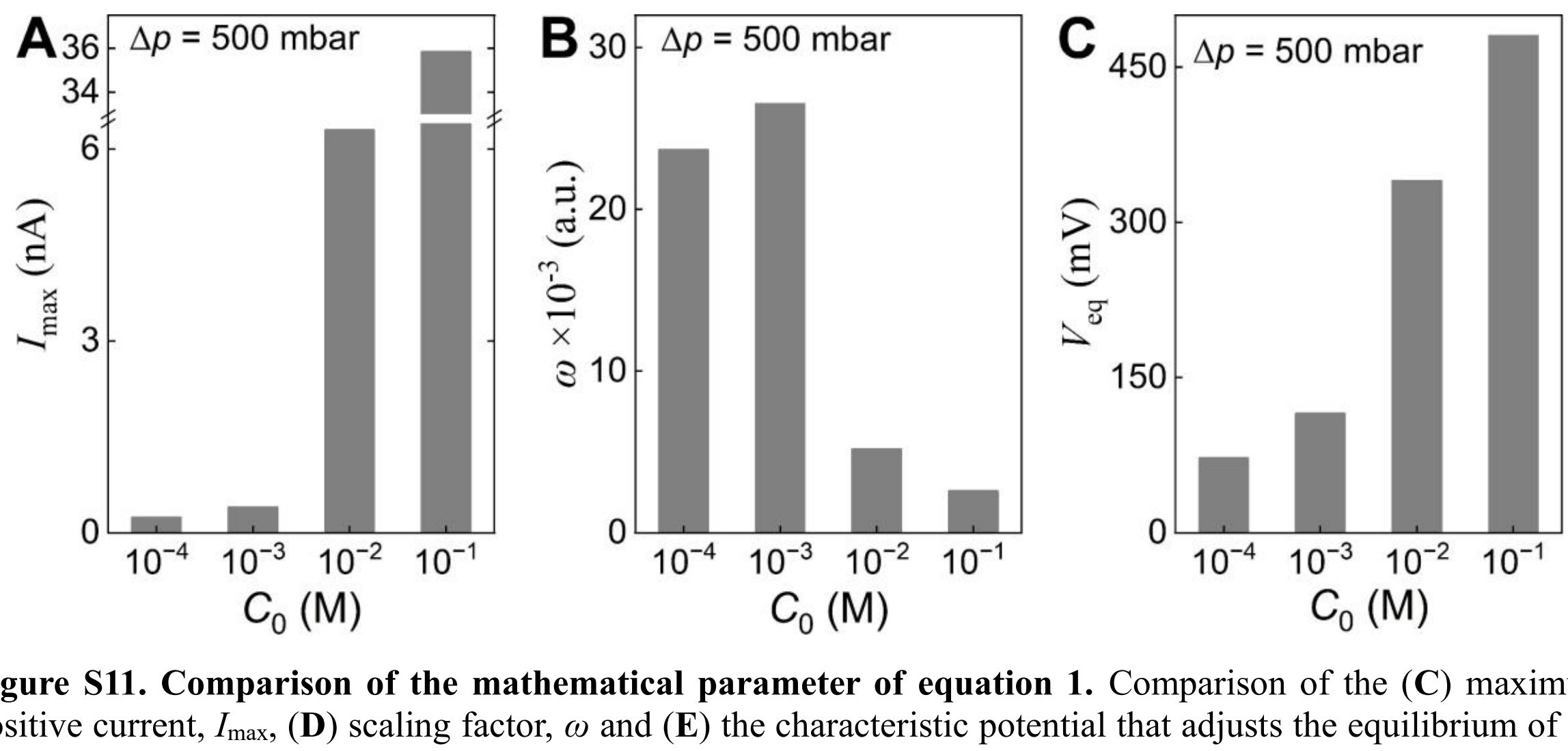

Figure S11. Comparison of the mathematical parameter of equation 1. Comparison of the (**C**) maximum positive current, I_{max}, (**D**) scaling factor, ω and (**E**) the characteristic potential that adjusts the equilibrium of the electric-field dependent ionic streaming, V_{eq}. These parameters are extracted by fitting the I_{stream} at Δp = 0.5 bar for different electric fields for the concentrations of the data shown in Figures S8, S9 and S10. *Device #4* is used in this figure.

S9. Computational model, governing equations and boundary conditions employed to model ionic transport across the channel

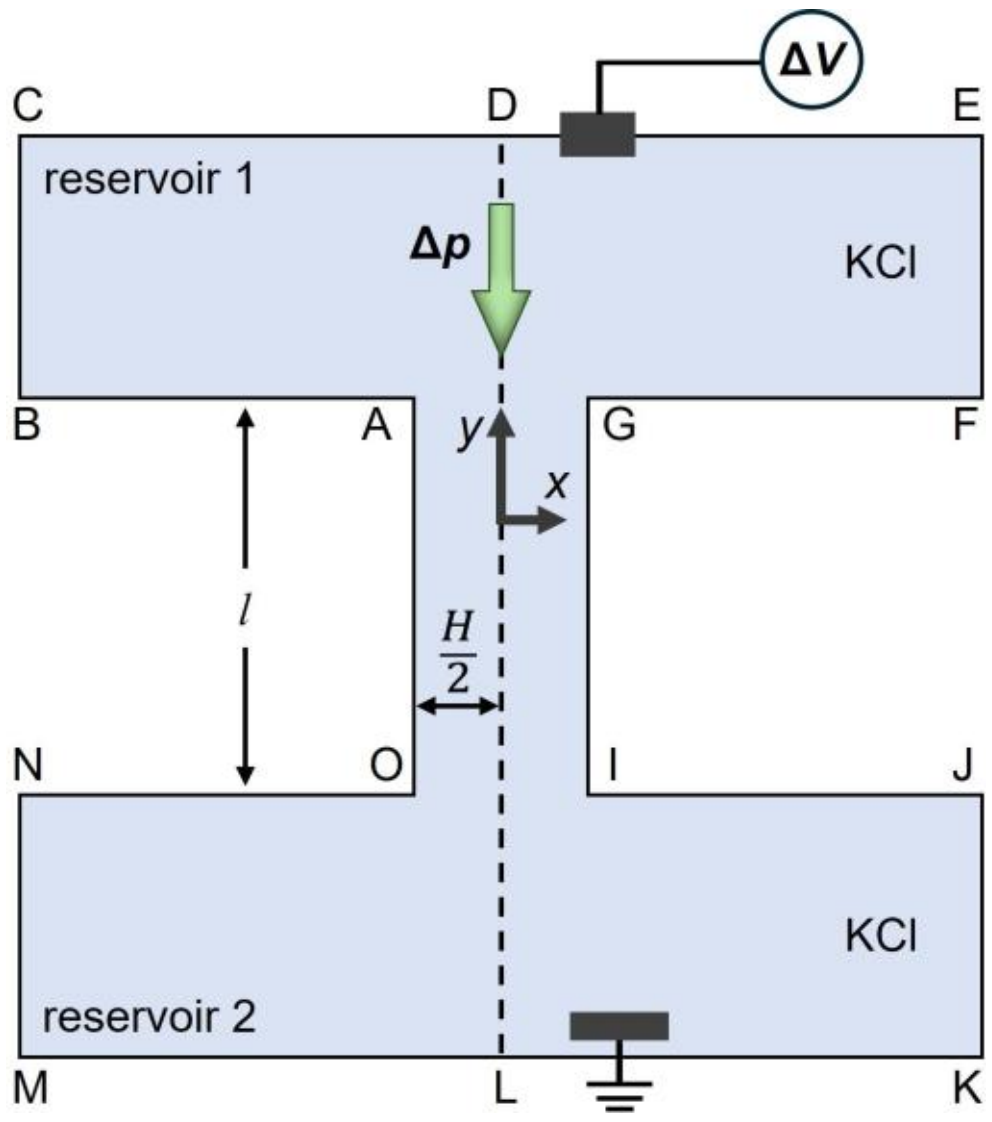

Figure S12. System schematic for the computational study.

S9.1. System Description. Figure S12 sketches the computational system of the nanochannel used for our numerical study. The channel centre lies on the origin of the cartesian coordinates (x, y) and the channel midplane coincides with the y-axis. The channel has the dimensions of 'H', 'l', and 'w' in the x, y, and z directions, which we refer to as the "length", "width", and "height" (or "thickness") of the channel, respectively. The channel separates two electrolytic reservoirs filled with a KCl solution of bulk salt concentration C_0, viscosity η of 1 mPa.s, density ρ of 1 g.cc^{-1}, dielectric constant ε_f of 80, diffusion coefficients of the ions in the solution as D_{K^+} = 1.95×10^{-9} m^2.s^{-1} and D_{Cl^-}= 2.03×10^{-9} m^2.s^{-1}. We assume a constant system temperature T of 298 K. The channel surfaces (AO and GI) carry a surface charge density of θ. A voltage ΔV and pressure differential Δp are applied along the negative-y axis. The ionic current (I) and streaming current (I_{stream}) are considered positive along the negative-y direction.

We discretize the 2D space enclosed by the segment ABCEFGIJKMNOA into triangular elements. We sufficiently refine the mesh in the channel and near its openings to precisely capture the high gradients of ionic concentration and electric potential in the region. The governing equations and their boundary conditions across the 2D computational space are described below.

S9.2. Electrostatics

The electric potential in the fluid ϕ is solved by the Poisson equation:

$$-\varepsilon_0\varepsilon_f\nabla^2\phi = e(C_+ + C_-) \qquad \text{S6}$$

Here, ε_0 is the electric permittivity in vacuum, ε_f is the relative permittivity of water, C_+ and C_- are the concentration of K^+ and Cl^- ions in the electrolytic fluid, respectively, and e is the charge of an electron. The applied voltage ΔV is imposed as

$$\phi_{EF} = \Delta V \ and\ \phi_{KM} = 0$$

The gradients of ϕ in the x-direction are significant only near the channel openings as ΔV is exerted in the negative y-direction. Thus, the component of electric field in the x-direction is taken to be zero on CB, NM, EF, and JK, because they are far away from the channel openings. On the channel surfaces, AN and GH, which carry a surface charge density of θ, we apply:

$$\boldsymbol{n}.(-\varepsilon_0\varepsilon_f\nabla\phi) = \theta \qquad \text{S7}$$

Here, **n** is the unit vector perpendicular to the surfaces pointing into the fluid. On the surfaces AB, MN, FG, and HI, which have a zero surface charge density, we apply $\mathbf{n}.(\nabla\phi) = 0$.

S9.3. Fluid Flow

Fluid transport is solved *via* the Navier-Stokes (NS) equations

$$\nabla.\boldsymbol{u} = 0$$

$$\rho[\mathbf{u}.\nabla\mathbf{u}] = -(\nabla p) + \eta(\nabla^2\mathbf{u}) - e(C_+ + C_-)\nabla\phi \qquad \text{S8}$$

Here, **u** is the fluid velocity, p is the pressure, and the '$-e(C_+ - C_-)\nabla\phi$' is the electrostatic body force acting on the electrolytic fluid. To exert a pressure drop Δp along the channel, we set

$$p_{EC} = p_{KM} = \frac{\Delta p}{2}$$

In addition, we set a zero shear stress in the x-direction at the inlet and outlet, *i.e.*, EC and KM. No slip and no penetration fluid flow boundary conditions are imposed on AB, AO, NO, FG, GI, and IJ. We apply the "no shear stress" and "zero velocity in the x-direction" boundary conditions on CB, NM, EF, and JK.

S9.4. Ion Transport

The flow of ions is governed by the Nernst-Planck (NP) equations,

$$\nabla \cdot \boldsymbol{J}_\pm = 0\nabla$$

$$\boldsymbol{J}_\pm = \mathbf{u}C_\pm - D_\pm\nabla C_\pm - \left[\pm\frac{D_\pm}{k_B T}eC_\pm\nabla\phi\right] \qquad \text{S9}$$

where k_B is the Boltzmann constant, $\boldsymbol{J}_+$ ($\boldsymbol{J}_-$) is the ionic flux of the K^+ (Cl^-) ions in the fluid, and the terms on the right-hand side of $\boldsymbol{J}_\pm$ are its advective, diffusive, and electromigration components, respectively. We apply a zero normal ionic flux on AB, AO, NO, FG, GI, IJ, CB, NM, EF, and JK. At the reservoirs, we set $(C_\pm)_{EC} = (C_\pm)_{KM} = C_0$.

S9.5. Generating results

The ionic current (I) passing through the slit is calculated as

$$I = \int w.e\left(\boldsymbol{J}_{+,y} - \boldsymbol{J}_{-,y}\right)dx \qquad \text{S10}$$

carried out far away from the channel openings and deep into either reservoir to attain precise results. Here, $\boldsymbol{J}_{+,y}$ ($\boldsymbol{J}_{-,y}$) is the y-component of the ionic flux of K^+ (Cl^-) ions, and 'w' is the channel width along the z direction. We ensure that the measured ionic current is accurate, grid-independent, and unaffected by the effective reservoir dimensions (by sufficiently elongating CE, EF, and JK).

S9.6. Reasons for the selection of our computational method and a nanoscale channel height in our computational system

While the continuum-based PNP-NS model has been used successfully to describe and analyze ion transport in several nanoconfined systems consisting of nanoscale pores and channels, its validity becomes questionable at Å-scale dimensions, since the continuum assumptions break down at such length-scales (below ~ 1.66 nm).[6] Consequently, a H of 6.3 Å cannot be reliably represented within the PNP-NS framework. However, this computational approach has been used previously to successfully replicate and analyze the electrokinetic ion transport characteristics in nanopores with diameters as small as 2 to 4 nm.[7] So, we adopt a channel height of 2 nm, as it represents the smallest practically feasible dimension at which the PNP–NS numerical simulations can be carried out. This H, furthermore, is small enough to induce electrical double layer (*EDL*) overlap,[3] which is an inevitability in the Å-scale slits and is known to influence ionic flow significantly.

Meanwhile, a channel height of 6.3 Å could have been considered if we used MD simulations as our method of computational analysis here. However, we did not employ MD simulations in this study for

two major reasons. Firstly, the experimental setup consists of long channels, for which MD simulations need inordinately high computational time. Secondly, previous studies in the domain of dual-stimuli-gated electrokinetic ion transport show that excessively high magnitudes of pressure drop need to be applied in MD simulations to replicate the ion transport phenomena exhibited in experimental setups at much lower magnitudes of pressure drop. For instance, while the symmetric linear growth of ionic current with applied pressure in 3.4 Å long graphene nanopores[8] is observed experimentally under pressure drops (Δp) of the order of bars, demonstrating similar effects in an MD simulation required Δp of the order of gigapascals. Meanwhile, our experiments consider much longer channels (with channel lengths of the order of micrometres). Hence, analyzing mechano-sensitive ion transport in such channels using MD simulations can require the imposition of Δp that is further 3 to 4 orders of magnitude higher, conditions under which the reliability of the results would be uncertain.

Based on these aspects, we decided that using a numerical simulation based on the continuum PNP-NS framework and employing it in a computational system/setup with a channel height of 2 nm is our best possible/viable option to numerically analyse the ion transport characteristics detected experimentally across the 6.3 Å-thick VM channels. While these numerical simulations adopting a 2 nm-thick channel cannot replicate the exact conditions of ionic flow that occur within the 6.3 Å-thick VM channels, they can still help us understand the effects of the simultaneous application of a voltage (ΔV) and a pressure drop (Δp) across the VM channels on their ion transport characteristics, which was one of our primary objectives to begin with. This is because we expect the effects of the simultaneously applied ΔV and Δp on the ionic flow across a 6.3 Å-thick channel to share significant similarities with, if not be the same as, those across a 2 nm-thick channel. With the numerical simulations being able to mimic the majority of the features of the experimental I_{stream}-V curves (at different Δp) and helping us analyze their origins, albeit in a 2 nm-thick channel, by visualizing how ΔV and Δp alter the quasi-static ionic distribution in the channel, they have helped us approach that objective by revealing the potential electrohydrodynamic couplings at the root of the multi-stimuli-gated ion transport behavior detected in the VM channels.

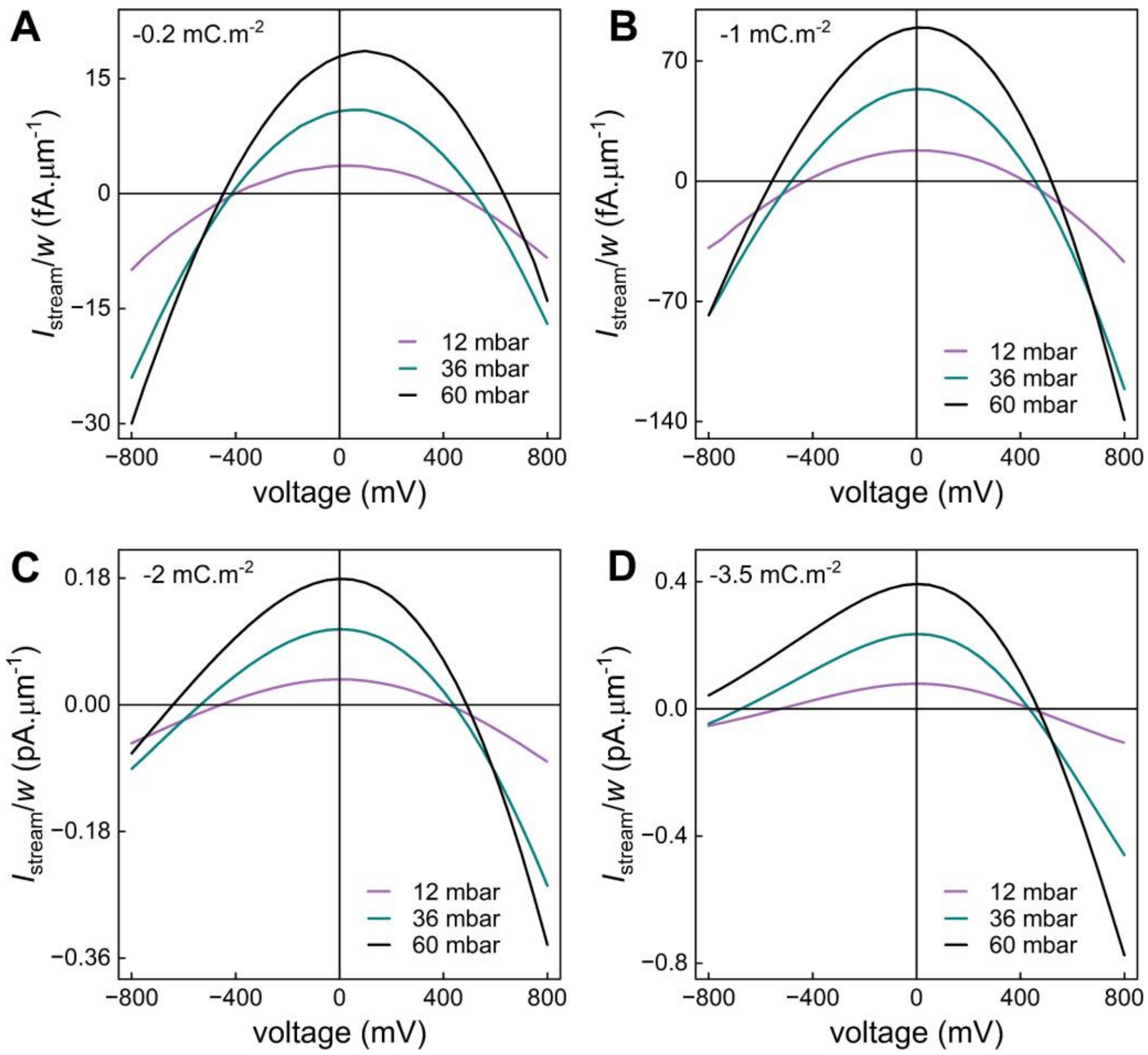

Figure S13. Dependency of surface charge density (θ). Variation of the streaming current per unit channel width (I_{stream}/w) with an applied voltage (ΔV) obtained numerically with θ = -0.2, -1, -2 and -3.5 m.Cm^{-2}, in (**A**), (**B**), (**C**), and (**D**), respectively, at Δp = 12, 36 and 60 mbar and at l = 0.26 µm, H = 2 nm and C_0 = 1 mM. 60 mbar graphs in (**B**), (**C**) and (**D**) are also represented in Figure 3D.

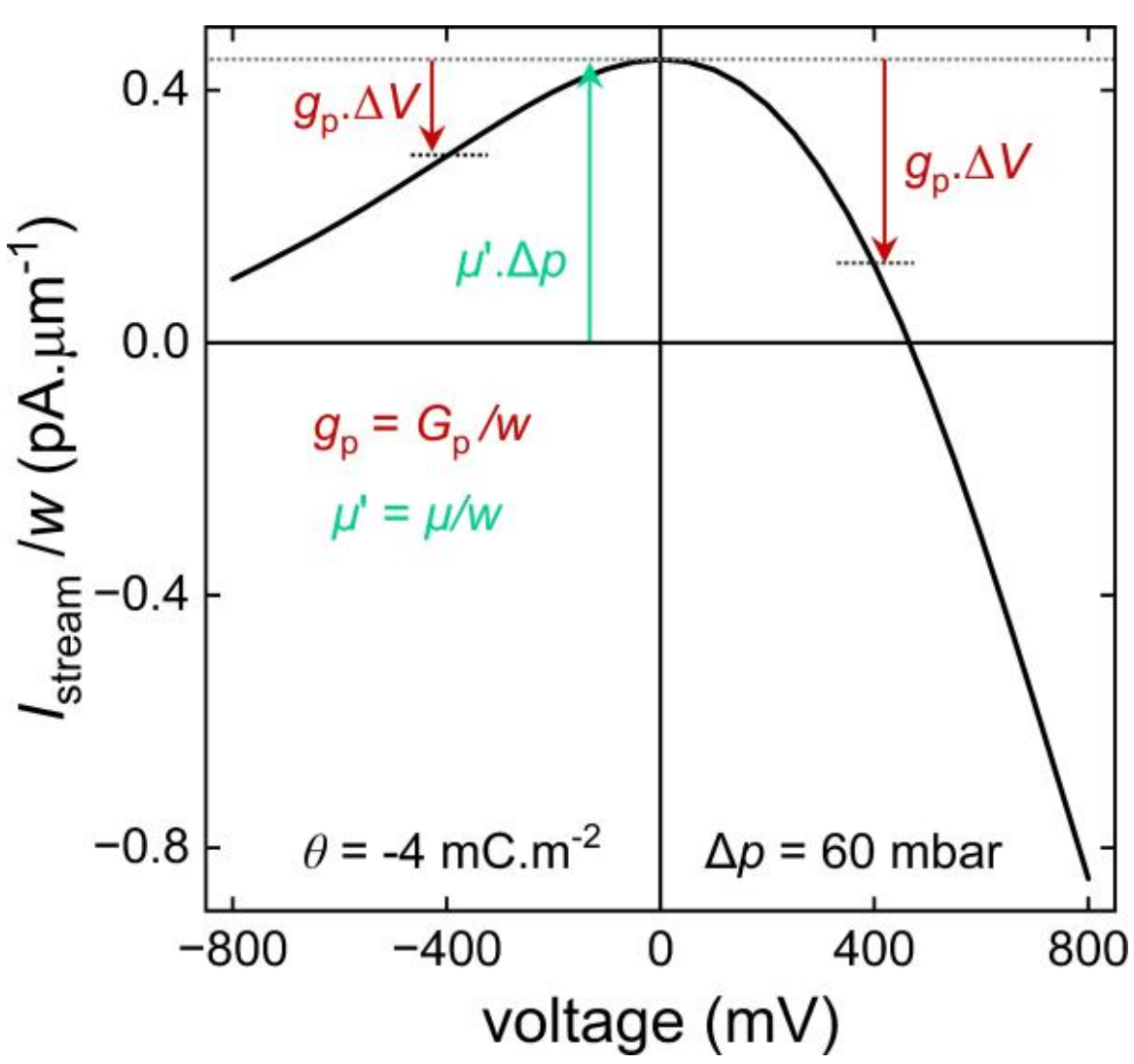

Figure S14. Illustration of the different components of I_{stream} in the numerically simulated (I_{stream}/w)-V curve from Figure 3D for θ = -4 mC.m^{-2} and Δp = 60 mbar at ΔV = ±400 mV.

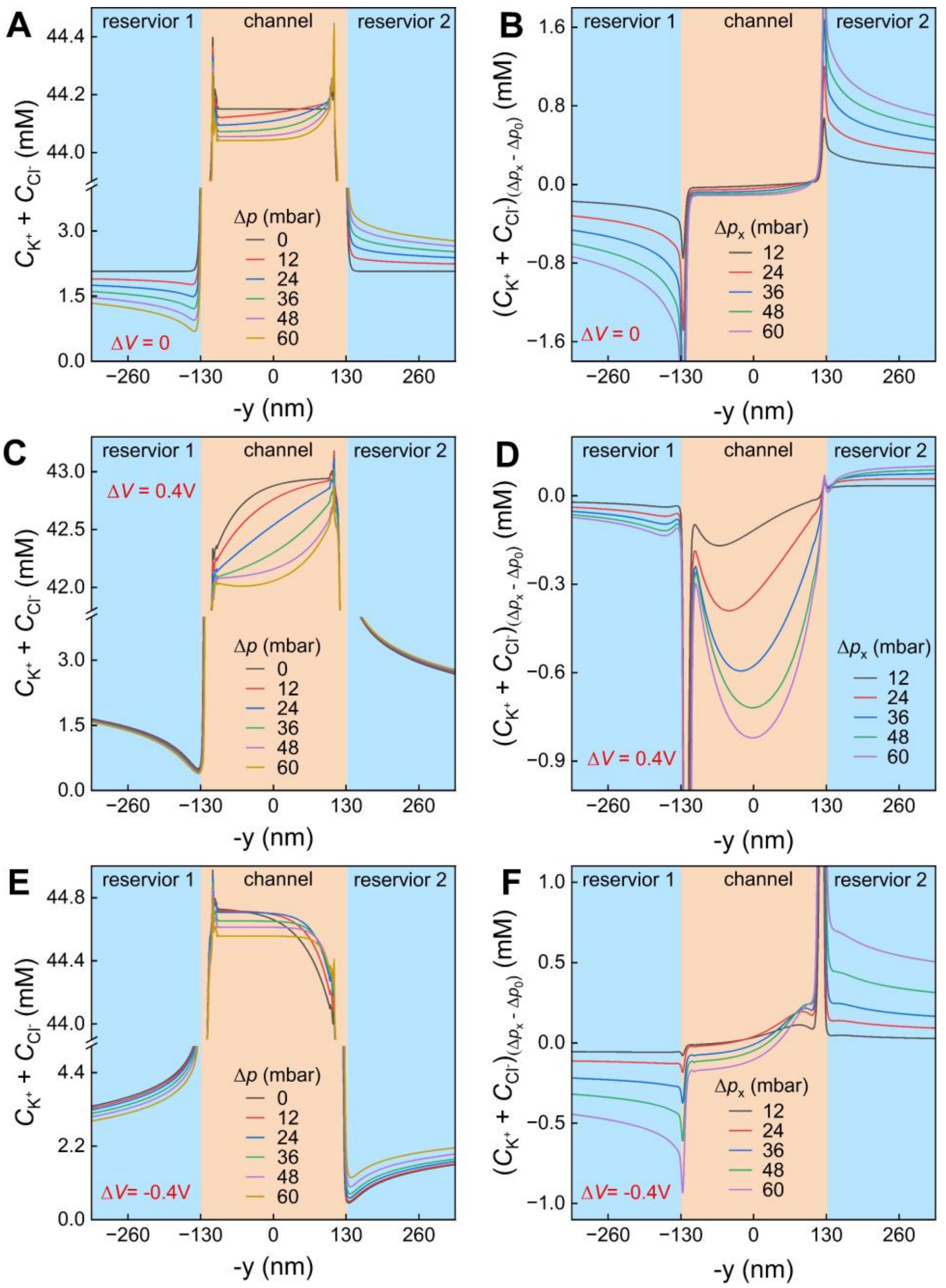

Figure S15. Total ionic concentration $(C_{K^+} + C_{Cl^-})$ along the mid-plane of the channel (i.e., x = 0, represented by segment DL in Figure S12) with θ = –4 mC.m[-2] at Δp = 0, 12, 24, 36, 48, and 60 mbar under ΔV of (**A**) 0 V, (**C**) +0.4 V and (**E**) –0.4 V. The difference in the total ionic concentration at different Δp from that a Δp = 0 {i.e. $(C_{K^+} + C_{Cl^-})_{(\Delta p_x - \Delta p_0)}$} at of (**B**) 0 V, (**D**) +0.4 V and (**F**) –0.4 V. The H = 2 nm, l = 0.26 µm and C_0 = 1 mM. The Δp = 0 and ΔV = 0 of (**A**), ΔV = -0.4 V with Δp = 0 and 60 mbar of (**C**) and ΔV = +0.4 V with Δp = 0 and 60 mbar of (**E**) are also represented in Figures 4A, 4B and 4C, respectively as bottom instes.

S9.7. Effect of *out-of-plane* dielectric constant

The *out-of-plane* dielectric constant ($\varepsilon_\perp$) of water is known to be significantly suppressed from its bulk value under angstrom-scale confinement, while the *in-plane* dielectric constant remains comparatively unchanged.[9] To examine whether this effect influences the ion transport characteristics detected in the VM channels, we perform additional simulations using $\varepsilon_\perp$ values of 2, 5, and 10 while maintaining H at 2 nm and the bulk *in-plane* dielectric constant (Figures S16A, S16B and S16C, respectively). Reducing $\varepsilon_\perp$ from 80 to 2 results in a decrease in the magnitude of the I_{stream} proportionately at the different values of ΔV and Δp considered (Figure S16D), which does not alter the shape of the I_{stream}-V curves. With decreasing $\varepsilon_\perp$, the magnitude of the surface-charge-generated electric field acting across the electrical double layer[10] of ions (in the x-direction), $|\theta|/\varepsilon_\perp$, increases. This intensifies the attraction

experienced by the counterions within the channel towards the channel surface, making it more difficult for the Δp-driven hydrodynamic flow to give rise to (i) the advection of the net excess counterions, and (ii) the deformation of the quasi-static distribution of ions across the channel (Figure S17), due to which both the phenomena get diminished. The weakening of the former reduces $|\mu|$ (and thus, $|\mu.\Delta p|$, *i.e.*, the magnitude of streaming current at $\Delta V = 0$), while the latter lowers $|G_\mathrm{p}|$ (and thus, $|G_\mathrm{p}.\Delta V|$, *i.e.*, the magnitude of the change in streaming current due to the application of ΔV). Please note that $G_\mathrm{p}.\Delta V$ can have two definitions: (i) the change in the voltage-driven ionic current ($G.\Delta V$) due to the exertion of Δp, as explained via equation (3) in the main manuscript, and (ii) the change in streaming current due to the exertion of ΔV, as explained *via* Figure S14. Since the magnitudes of the two components of I_stream, *i.e.*, $\mu.\Delta p$ and $G_\mathrm{p}.\Delta V$, are reduced by the same effect (*i.e.*, a drop in $\varepsilon_\perp$), their reductions are proportional. As a result, even though a reduction in $\varepsilon_\perp$ decreases the magnitude of I_stream, it does so proportionately at the different values of ΔV and Δp considered, due to which the characteristics of the I_stream-V response (at different Δp) remains unchanged. This indicates that the suppression of the *out-of-plane* dielectric constant of water under angstrom-scale confinements does not influence or determine the ion transport characteristics observed across the VM channels in our study.

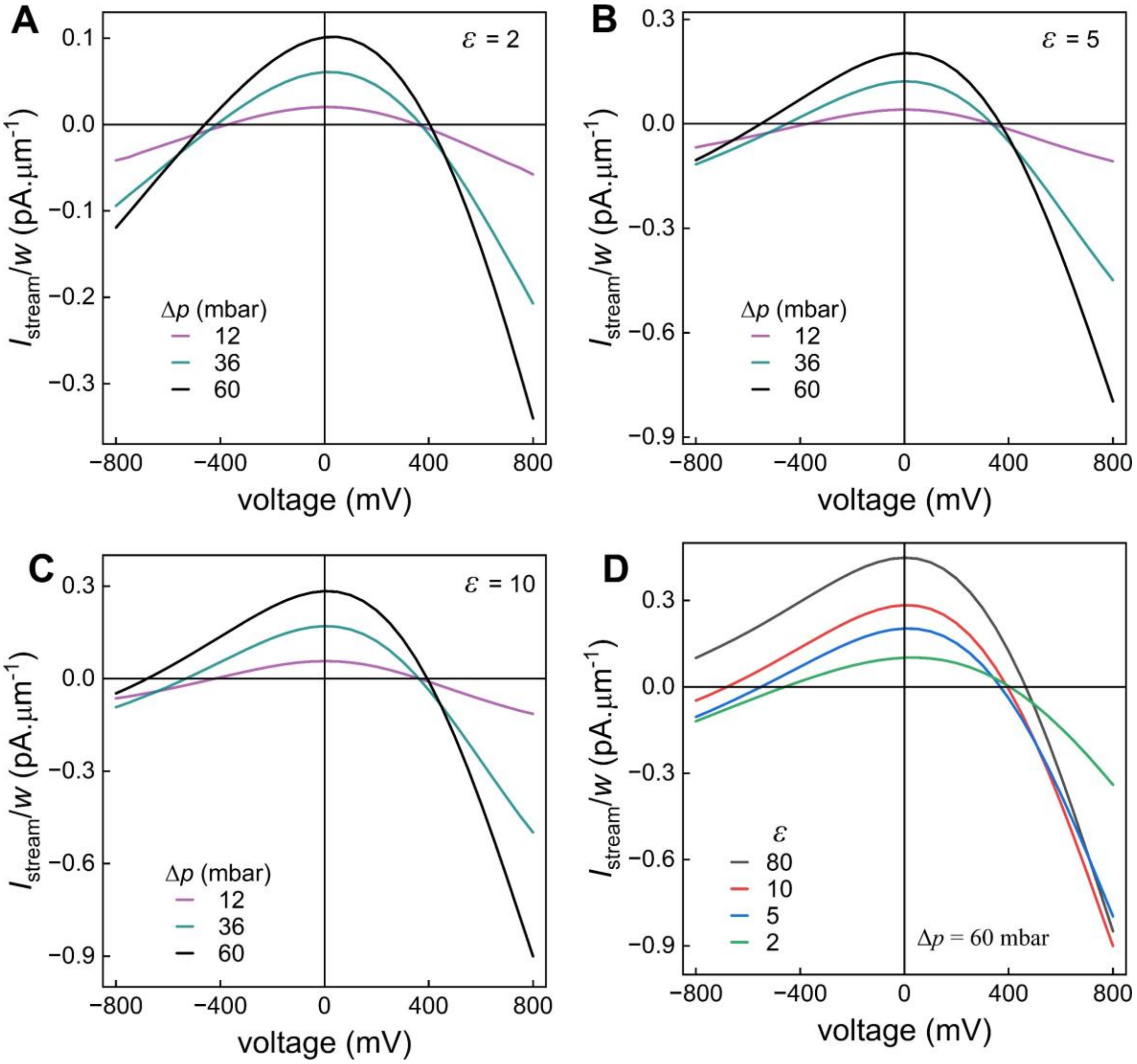

Figure S16. Dielectric constant dependent I_stream/w with ΔV. I_stream/w vs ΔV for dielectric constant of (**A**) 2, (**B**) 5 and (**C**) 10 at different Δp and (**D**) at Δp of 60 mbar for different dielectric constant. In this figure, θ = -4 mC.m^{-2}, C_0 = 1 mM KCl and channel dimensions of H = 2 nm, l = 0.26 µm.

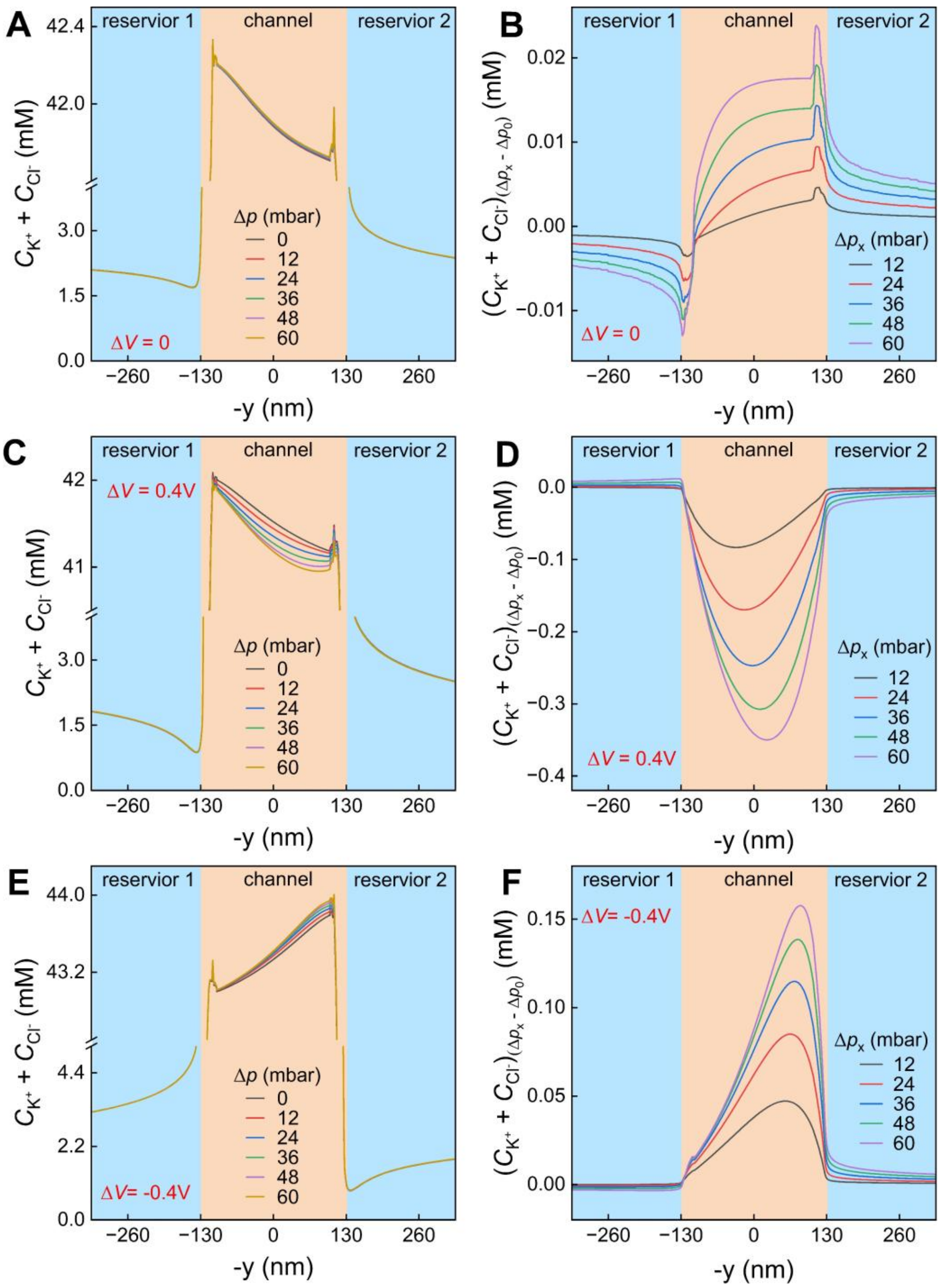

Figure S17. Effect of *out-of-plane* dielectric constant ($\varepsilon_\perp$ = 5) on total ionic concertation ($\boldsymbol{C_{K^+} + C_{Cl^-}}$) along the channel mid-plane (i.e., x = 0, represented by segment DL in Figure S12) for θ = –4 mC.m^{-2} at Δp = 0, 12, 24, 36, 48, and 60 mbar under ΔV of (**A**) 0 V, (**C**) +0.4 V and (**E**) –0.4 V. The difference in the total ionic concentration at different Δp from that a Δp = 0 {i.e. $(C_{K^+} + C_{Cl^-})_{(\Delta p_x - \Delta p_0)}$} at of (**B**) 0 V, (**D**) +0.4 V and (**F**) –0.4 V. Simulations were performed for H = 2 nm, l = 0.26 μm and C_0 = 1 mM.

S10. Building the modified surface charge regulation (*mSCR*) model

The conventional methods cannot describe the multi-stimuli responsive behaviours of ion channels, as they assume θ is a function of C_0 alone. To describe such ion transports, we develop a new model by including the dependency of the ΔV and Δp, i.e., $\theta = f(\Delta V, \Delta p, C_0)$. Firstly, the variation of θ with ΔV was determined at different values of Δp and C_0, reproducing characteristics of the experimentally obtained I_{stream}-V curves at different Δp (Figure 3C) and for varying C_0 (Figure 5D). The pH of the KCl electrolyte is ~6. Further, we calculate the average ionic concentrations (C_{avg}) across the channel, defined as the spatial average of cations and anions, $0.5(C_+ + C_-)$, under those conditions and correlate it with the corresponding θ to extract the function: $\theta = g(C_{avg})$. This process is illustrated in Figure S18 for the I_{stream}-V curves in Figures 3C and 5D. However, we were unable to mimic the curves at the 10 mM and 100 mM because of the requirement of extremely high θ, causing the numerical simulations fails to converge. Hence, we are limited to the values of θ and C_{avg} obtained for 0.1 mM and 1 mM to obtain the variation of the two quantities with C_0. These dependence of θ on C_{avg} are then correlated in Figure S19, where the two different branches display the positive and the negative values of θ (shown in inset of Figure S19) to mimic the I_{stream}-V curves in Figures 3C and 5D at the more positive and more negative values of ΔV, respectively.

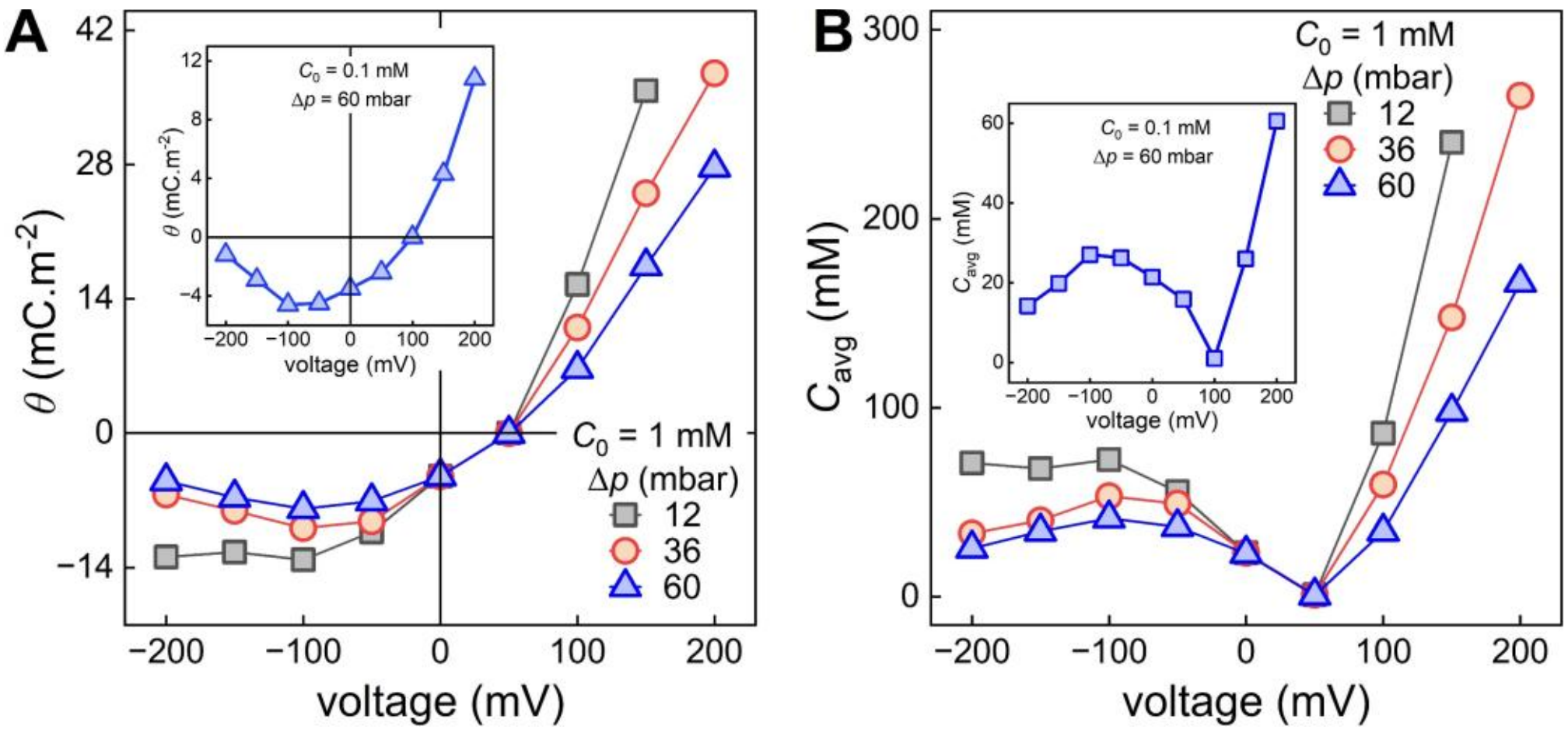

Figure S18. Numerical simulation of θ and C_{avg}. Variation of (**A**) θ and (**B**) C_{avg} with applied voltage ΔV at Δp = 12, 36, and 60 mbar and C_0 = 1 mM. The insets in (**A**) and (**B**) demonstrate the variation of θ and C_{avg} respectively, with ΔV at Δp = 60 mbar and C_0 = 0.1 mM. $C_{avg} = 0.5(C_+ + C_-)$ across the length (l) and height (H) of the slit (see Figure S12) and H = 2 nm and l = 0.26 µm.

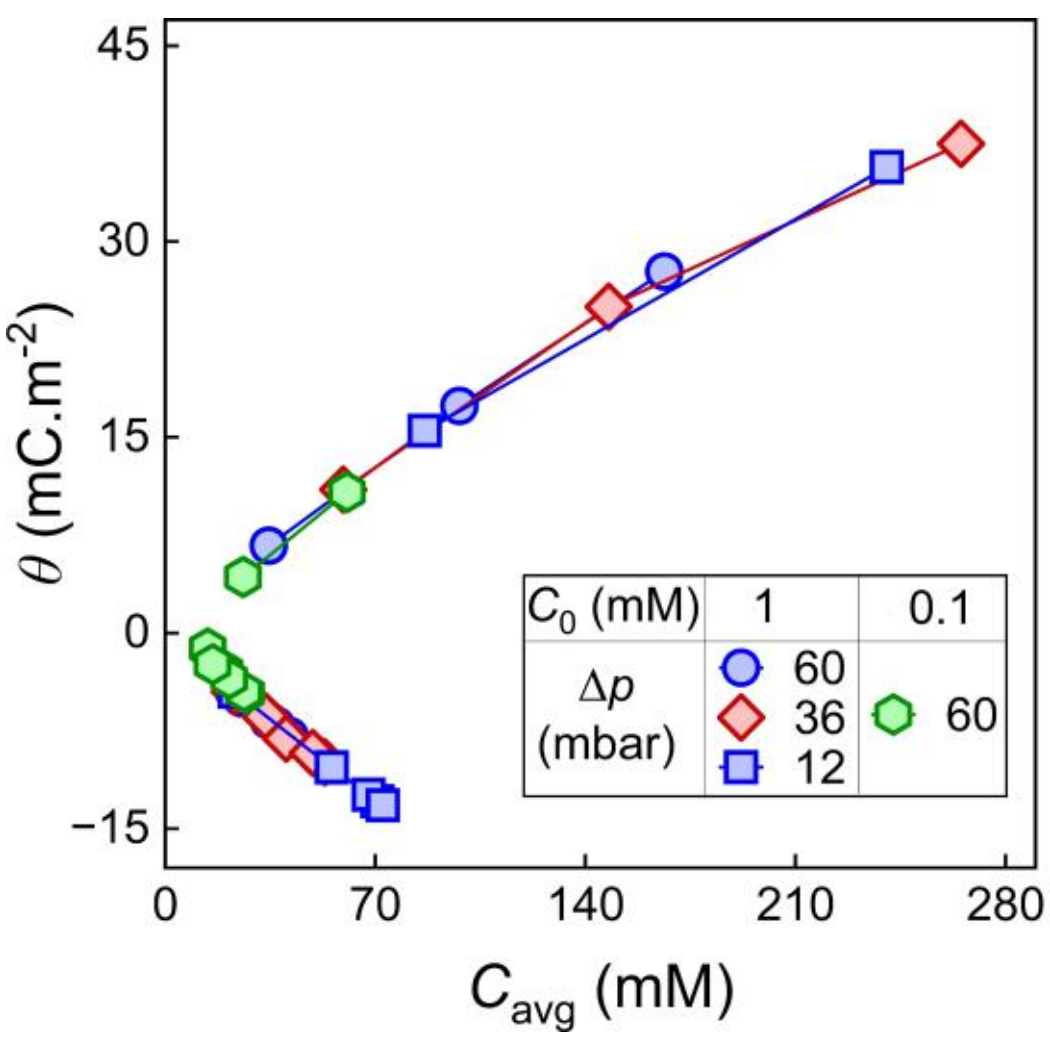

Figure S19. Correlation between θ vs C_{avg} obtained in Figure S18. This correlation is shown with explicit specification of the conditions under which θ and C_{avg} obtained and is then generalized in Figure 5C.

Next, the relation $C_{avg} = h(\Delta V, \Delta p, C_0)$ has been established by estimating the C_{avg} in the C_{avg}-ΔV curves from Figure S18B as absolute values of a fitted parabolic (quadratic) curve.

$$\text{i.e., } C_{avg} = |a(\Delta V)^2 + b\,(\Delta V) + c| \qquad \text{S11}$$

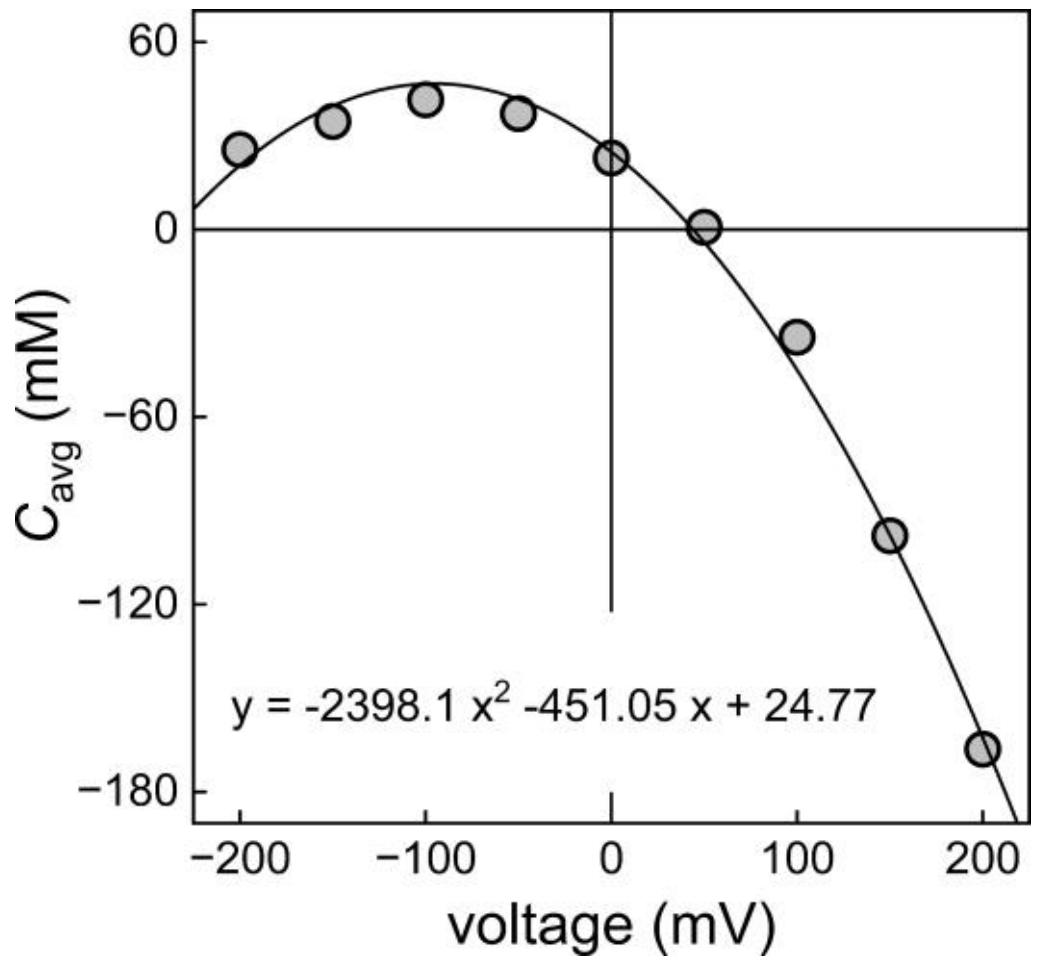

Figure S20. We depict our method of estimating the variation of the (spatially) averaged ionic concentration across the channel C_{avg} with the applied voltage ΔV in the C_{avg}-ΔV curves of Figure S18B using curve fitting. The values of C_{avg} in the C_{avg}-ΔV curves are estimated as the absolute values of a fitted parabolic curve that varies quadratically with ΔV. This example is for the C_{avg}-ΔV curve from Figure S18B at Δp = 60 mbar (C_0 = 1 mM). The circles depict the values taken from the curve in Figure S18B and the line represents the parabolic fitting curve.

The resulting approximations of the C_{avg}-ΔV curves in Figure S18B are written below (Table S2) and displayed in Figure S19.

***C*₀** (mM)	***Δp*** (mbar)	***a*** (mM.V^{-2})	***b*** (mM.V^{-1})	***c*** (mM)
1	60	-2398.1	-451.05	24.77
	36	-3682	-677.44	31.17
	12	-4335.77	-977.79	30.65
0.1	60	-1173.41	-166.31	23.89

Table S2. Estimation of *a*, *b*, and *c* for Figure S18B using equation S11.

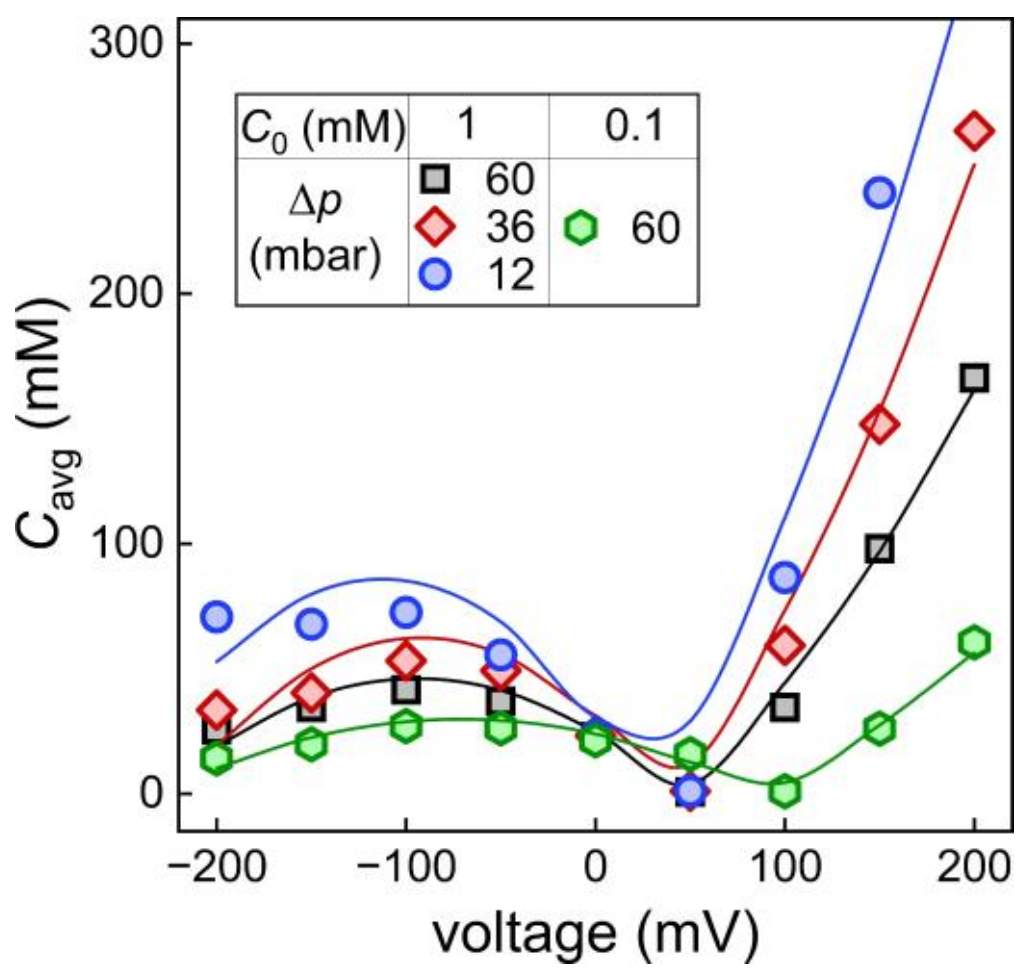

Figure S21. The C_{avg}-ΔV curves from Figure S18B are displayed with their corresponding fitting curves obtained by the equation S11.

The coefficients of these quadratic expressions (*i.e.*, a, b, and c) are then expressed as a function of Δp and C_0, as described below:

$$a = \frac{1}{-2398.1}\left[\left\{0.547\left(\frac{\Delta p}{\text{mbar}}\right)^2 + 0.98\left(\frac{\Delta p}{\text{mbar}}\right) - 4426.3\right\}\left\{-1360.7\left(\frac{C_0}{\text{mM}}\right) - 1037.3\right\}\right].\text{mM}.\text{V}^{-2} \quad \text{S12}$$

$$b = \frac{1}{-451.1}\left[\left\{-0.064\left(\frac{\Delta p}{\text{mbar}}\right)^2 + 15.59\left(\frac{\Delta p}{\text{mbar}}\right) - 1155.7\right\}\left\{-316.38\left(\frac{C_0}{\text{mM}}\right) - 134.7\right\}\right].\text{mM}.\text{V}^{-1} \quad \text{S13}$$

$$c = \frac{1}{24.8}\left[\left\{-0.006\left(\frac{\Delta p}{\text{mbar}}\right)^2 + 031\left(\frac{\Delta p}{\text{mbar}}\right) + 27.8\right\}\left\{0.97\left(\frac{C_0}{\text{mM}}\right) + 23.8\right\}\right].\text{mM} \quad \text{S14}$$

If we input Δp in mbar and C_0 in mM in the expressions above, we obtain a, b, and c in mM.V^{-2}, mM.V^{-1}, and mM, respectively. Substituting the expressions of a, b, and c (equations S12, S13, and S14 respectively) in equation S11 generates $C_{\text{avg}} = h(\Delta V, \Delta p, C_0)$, which when substituted in the expressions $\theta = g(C_{\text{avg}})$ generates the *mSCR* relation as:

$$\theta = \text{g}(h(\Delta V, \Delta p, C_0)) = f(\Delta V, \Delta p, C_0) \quad \text{S15}$$

In general, the expressions of a, b, and c can be written as:

$$a = (1/\Omega_1)[\alpha_1(\Delta p)^2 + \beta_1(\Delta p) + \gamma_1][\nu_1 C_0 + \eta_1] \quad \text{S16}$$
$$b = (1/\Omega_2)[\alpha_2(\Delta p)^2 + \beta_2(\Delta p) + \gamma_2][\nu_2 C_0 + \eta_2] \quad \text{S17}$$
$$c = (1/\Omega_3)[\alpha_3(\Delta p)^2 + \beta_3(\Delta p) + \gamma_3][\nu_3 C_0 + \eta_3] \quad \text{S18}$$

Substituting equations S16, S17 and S18 in S11, C_{avg} can be expressed as:

$$C_{avg} = |C_0[\alpha_{11}(\Delta p)^2(\Delta V)^2 + \beta_{11}(\Delta p)(\Delta V)^2 + \gamma_{11}(\Delta V)^2 + \alpha_{11}(\Delta p)^2(\Delta V) + \beta_{21}(\Delta p)(\Delta V) + \gamma_{21}(\Delta V) + \alpha_{31}(\Delta p)^2 + \beta_{31}(\Delta p) + \gamma_{31}] + [\alpha_{12}(\Delta p)^2(\Delta V)^2 + \beta_{12}(\Delta p)(\Delta V)^2 + \gamma_{12}(\Delta V)^2 + \alpha_{22}(\Delta p)^2(\Delta V) + \beta_{22}(\Delta p)(\Delta V) + \gamma_{22}(\Delta V) + \alpha_{32}(\Delta p)^2 + \beta_{32}(\Delta p) + \gamma_{32}]| \quad \text{S19}$$

or,

$$C_{avg} = |[\alpha_{11}C_0 + \alpha_{12}](\Delta p)(\Delta V)^2 + [\beta_{11}C_0 + \beta_{12}](\Delta p)(\Delta V)^2 + [\gamma_{11}C_0 + \gamma_{12}](\Delta V)^2 + [\alpha_{21}C_0 + \alpha_{22}](\Delta p)^2(\Delta V) + [\beta_{21}C_0 + \beta_{22}](\Delta p)(\Delta V) + [\gamma_{21}C_0 + \gamma_{22}](\Delta V) + [\alpha_{31}C_0 + \alpha_{32}](\Delta p)^2 + [\beta_{31}C_0 + \beta_{32}](\Delta p) + [\gamma_{31}C_0 + \gamma_{32}]| \quad \text{S20}$$

Further, equation S20 can be simplified as

$$C_{\text{avg}} = |\, [\alpha.C_0 + \beta] \times [(\gamma.\Delta p + \delta)^2 + \chi] \times [(\lambda.\Delta V + \nu)^2 + \tau] + \kappa \,| \quad \text{S21}$$

where α, β, γ, δ, χ, λ, ν, τ, and κ are constants based on α_1, β_1, γ_1, ν_1, η_1, Ω_1, α_2, β_2, γ_2, ν_2, η_2, Ω_2, α_3, β_3, γ_3, ν_3, η_3, and Ω_3 from the expressions of a, b, and c above.

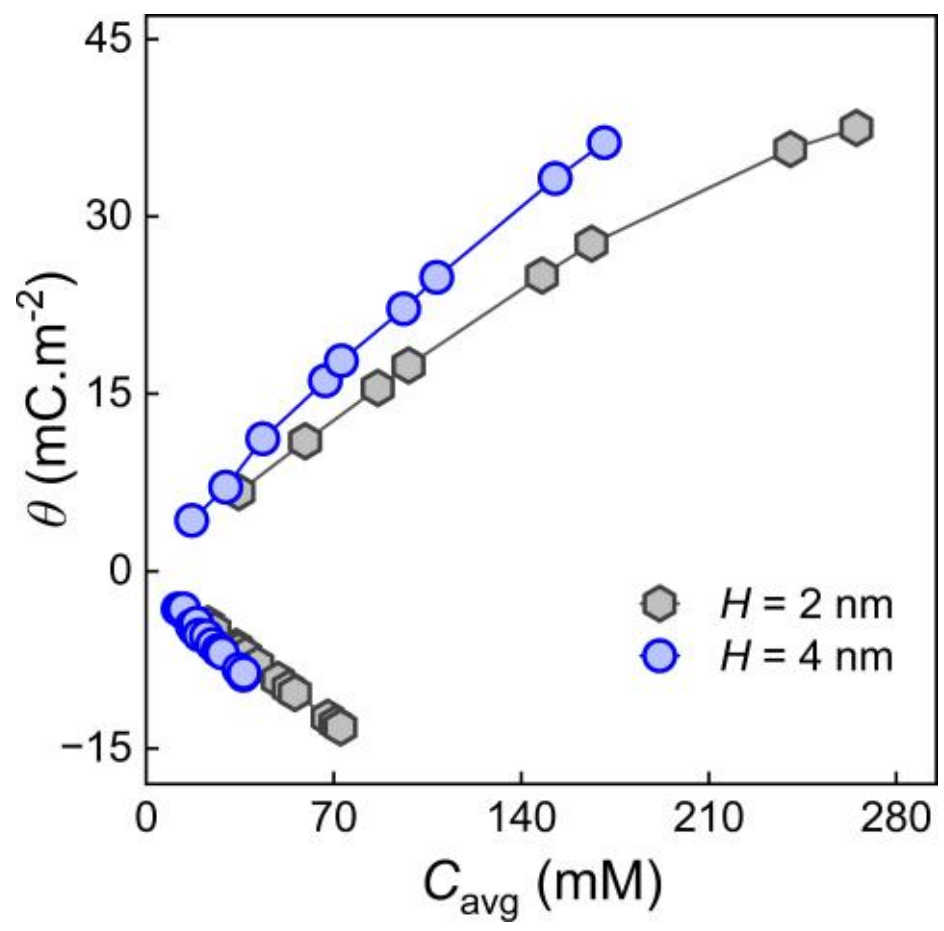

Figure S22. The operations carried out to obtain the results of Figure 5C (where H was taken as 2 nm) are repeated here using H = 4 nm in the numerical setup. The resultant θ-C_{avg} relation is shown here and compared with that obtained previously in Figure 5C of the main manuscript.

S11. Effect of pH into the *mSCR* model

Using the results shown in Figure 2D of the main manuscript, the influence of the electrolytic pH on the surface charge density (θ) was incorporated into the *mSCR* framework at specific values of Δp and C_0. If the *mSCR* equations, $\theta = A.C_{avg} + B$ and $C_{avg} = |a.(\Delta V)^2 + b.(\Delta V) + c|$, are calculated at a certain pH, which they were at pH = 6 in *section 10* of SI, the effect of a change in pH can be integrated into these equations by adjusting the value of c, as shown below for a given combination of Δp and C_0.

Figure 2D exhibits the variation of the ionic current with pH of KCl solution for varying Δp at $\Delta V = 0$. From that data, we derive I_{stream} at the same conditions using the technique demonstrated in Figure 2A.

The streaming current at $\Delta V = 0$, *i.e.*, $I_{stream}(\Delta V = 0, \Delta p) = \mu.\Delta p$, arises from the advection of the net excess counterions in the KCl solution (electrolyte) by the Δp-driven hydrodynamic flow.

∴ $I_{stream}(\Delta V = 0, \Delta p) \propto$ the quantity of net excess counterions in the KCl solution.

Meanwhile, the quantity of the net excess counterions in the electrolytic solution is proportional to the surface charge density (θ) of the VM channels.

∴ $I_{stream}(\Delta V = 0, \Delta p) \propto \theta$.

From Figure 2D, where C_0 = 1 mM, we estimate the I_{stream} at $\Delta V = 0$ and a particular Δp, say 500 mbar, for different values of pH, as shown in Table S3.

pH	I_{stream} (nA)
6	0.2838
3.5	0.2169
2.5	0.0696

Table S3. Variation of I_{stream} with *pH* at $\Delta V = 0$, Δp = 500 mbar, and C_0 = 1 mM (from Figure 2D).

However, since $I_{stream}(\Delta V = 0, \Delta p) \propto \theta$, and $\theta(\Delta V = 0, \Delta p = 500$ mbar, $pH = 6)$ was taken as -4 mC.m^{-2}, the variation of θ with pH at $\Delta V = 0$, Δp = 500 mbar, and C_0 = 1 mM, is obtained as shown in Table S4.

pH	θ (mC.m^{-2})
6	-4
3.5	-3.057
2.5	-0.98

Table S4. Variation of θ with pH at $\Delta V = 0$, Δp = 500 mbar, and C_0 = 1 mM.

A fitting curve for these points in the pH-θ space is: $\theta_f = -4.05 + 3.\exp[(-1.12(\text{pH} + 2.5)]$ mC.m^{-2}.

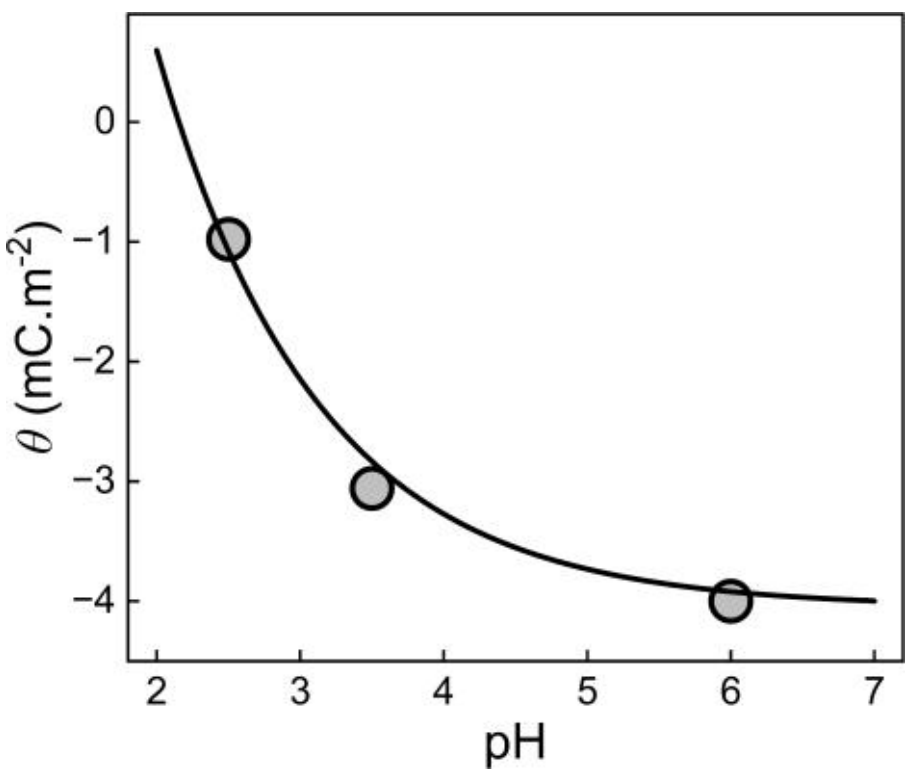

Figure S23. The variation of θ with pH, as obtained in Table S4, is displayed. The exponential fitting curve of the data is depicted by the solid line, with the corresponding fitting equation and fitting parameters shown in the plot.

In the *mSCR* model,

$$\theta = \text{A}.C_{\text{avg}} + \text{B}, \text{ where } C_{\text{avg}} = |a(\Delta V)^2 + b\,(\Delta V) + c|$$

At $\Delta V = 0$, for a fixed Δp and C_0, considering 500 mbar and 1 mM, respectively (note that Δp of 100, 300, and 500 mbar in the experimental setup were scaled to 12, 36, and 60 mbar in the numerical setup):

$C_{\text{avg}} = (\theta - \text{B})/\text{A}$ (A and B are selected for the negative-θ branch, as $\theta < 0$ at $\Delta V = 0$), and $C_{\text{avg}} = |c|$

$$\therefore \quad c = (\theta - \text{B})/\text{A} \qquad \text{S22}$$

Therefore, for a change in pH from the reference value (pH_R) of 6 (which we considered in building the *mSCR* in *section 10*), the corresponding changes in surface charge density (θ) and the parameter c are $\Delta\theta$ and Δc, respectively.

From equation 32, $\Delta\theta$ and Δc are related as:

$$\Delta c = \Delta\theta/\text{A} \qquad \text{S23}$$

Considering $\theta_\text{R} = \theta(\text{pH}_\text{R}) = -4$ mC.m^{-2} and $c_\text{R} = c(\text{pH}) = 24.77$ mM (see *section 10* of SI), as references, equation R2 can be expressed as:

$$[\theta(\text{pH}) - \theta(\text{pH}_\text{R})]/A = c_{\text{adj}} - c_R \quad or \quad [\theta(\text{pH}) - \theta_\text{R}]/A = c_{\text{adj}} - c_\text{R}$$

$$c_{\text{adj}} = c_R + ([\theta(\text{pH}) - \theta_\text{R}]/\text{A}). \qquad \text{S24}$$

Now, replacing θ(pH) with the fitting curve θ_f(pH) obtained in Figure S23, we get

$$c_{\text{adj}} = c_R + ([\theta_f\,(\text{pH}) - \theta_\text{R}]/A), \qquad \text{S25}$$

which can be solved to obtain c_{adj} (the "adjusted value of c" due to pH change).

Substituting the parameter c in the *mSCR* equation with the "pH-adjusted c" (or c_{adj}) yields the pH-adjusted *mSCR* equations can be expressed as:

$$\theta = (\text{A}.C_{\text{avg}} + \text{B}), \text{ where } C_{\text{avg}} = |a(\Delta V)^2 + b\,(\Delta V) + c_{\text{adj}}|. \qquad \text{S26}$$

This way, for a specific Δp and C_0, the effect of a change in the pH of the electrolytic solution can be taken into account in the *mSCR* model.

Here, c_{adj} is a function of Δp, C_0, and pH, whereas the original parameter c, calculated in *Section 10* of the SI at the reference pH of 6, depends only on Δp and C_0. Therefore,

$$c_{adj}(\Delta p, C_0, \mathrm{pH}) = c(\Delta p, C_0) + \Delta c(\Delta p, C_0, \mathrm{pH}) \quad \text{S27}$$

where, Δc is calculated to find c_{adj} at any pH using the above method. This helps us find the value of θ at any pH, but only for a specific combination of Δp and C_0 at a time, which remains a drawback of the above method, as we could not add pH-dependent terms explicitly into the *mSCR* equations that would be valid under all conditions.

S12. Analytical measurement of ionic and streaming current

In the expression of ionic current:

$$I = G.\Delta V + \mu.\Delta p \quad \text{S28}$$

G can be expressed as:

$$G = \left(\frac{1}{G_{\text{channel}}} + \frac{2}{G_{\text{access}}}\right)^{-1} \quad \text{S29}$$

where, G_{channel} and G_{access} are the channel and access conductance.

The G_{channel} is related to σ_{channel} (channel conductivity) as:

$$G_{\text{channel}} = \sigma_{channel}\frac{A}{l} \quad \text{S30}$$

The G_{access} is related to σ_{access} (access conductivity) as:

$$G_{\text{access}} = 4\sigma_{access}R \quad \text{S31}$$

where R is conceptual access radius which has similar order of dimension as $H/2$.

In our case, $l >> H$, hence $(G_{\text{channel}})^{-1} >> (G_{\text{access}})^{-1}$

Thus, $$G = G_{\text{channel}} = \sigma_{channel}\frac{A}{l} \quad \text{S32}$$

Ionic current of fluids flowing through a channel can be expressed as:

$$I = F(C_+ - C_-)\mu A \quad \text{S33}$$

Electroneutrality in the channel dictates:

$$(C_+ - C_-)lA = -\theta lw$$
$$\Rightarrow (C_+ - C_-)lwH = -\theta lw$$
$$\Rightarrow (C_+ - C_-) = -\frac{\theta}{H} \quad \text{S34}$$

Stream current can be expressed (κ = permeance):

$$\mu\Delta p = -\frac{F\theta\kappa\Delta p}{H} \quad \text{S35}$$

By considering $$\sigma_{channel} = \frac{2F^2D}{RT}C_{avg}, \quad \text{S36}$$

ionic current can be expressed as:

$$I = \frac{2F^2DC_{\text{avg}}\Delta V}{RT}\frac{A}{l} - \frac{F\theta\kappa\Delta p}{H} \quad \text{S37}$$

In our study, we observed that: $$\frac{2F^2DC_{\text{avg}}\Delta V}{RT}\frac{A}{l} \gg \frac{F\theta\kappa\Delta p}{H}$$

Thus, equation S37 can be expressed as:

$$I = \frac{2F^2DC_{\text{avg}}\Delta V}{RT}\frac{A}{l} \quad \text{S38}$$

To obtain the I_{stream}-V curves at different values of C_0 in Figure 5E we substitute $C_{avg} = h(\Delta V, \Delta p, C_0)$ in the below expression:

$$I_{\text{stream}}(\Delta V, \Delta p) = I(\Delta V, \Delta p) - I(\Delta V) \quad \text{S39}$$

S13. Electrolyte dependent I_{stream} of VM channels

We performed voltage dependent I_{stream} for NaCl, $CaCl_2$ and $MgCl_2$ in addition to KCl. The variation of ionic current with different Δp and ΔV for a VM channels device is shown in Figures S24A, S24B, S24C and S24D for KCl, NaCl, $CaCl_2$ and $MgCl_2$, respectively. For KCl and NaCl we took 1 mM and $CaCl_2$ and $MgCl_2$ we took 0.5 mM concentrations to maintain an equivalent ionic strength of 1 mN. The I_{stream}–V curves at Δp = 500 mbar for different salts are compared in Figure S25. At the same equivalent ionic strength (*i.e.* 1 mN), the magnitude of the I_{stream} at $\Delta V = 0$ is lower for the divalent electrolytes $CaCl_2$ and $MgCl_2$ than the monovalent electrolytes KCl and NaCl. This is due to the higher resistance pathways for the divalent cations during the ion transport, which can be evident from the G (Figure 26B) of the channels with the cations in the electrolyte solution maintaining the same ionic strength. To further confirm that the device is not fully blocked by the ions, we compare the pressure responsive ionic current before and after performing the I_{stream}-V. Similarities in the ionic current values (Figures 29C and 29D for $CaCl_2$ and $MgCl_2$, respectively) confirms the VM channel is not blocked.

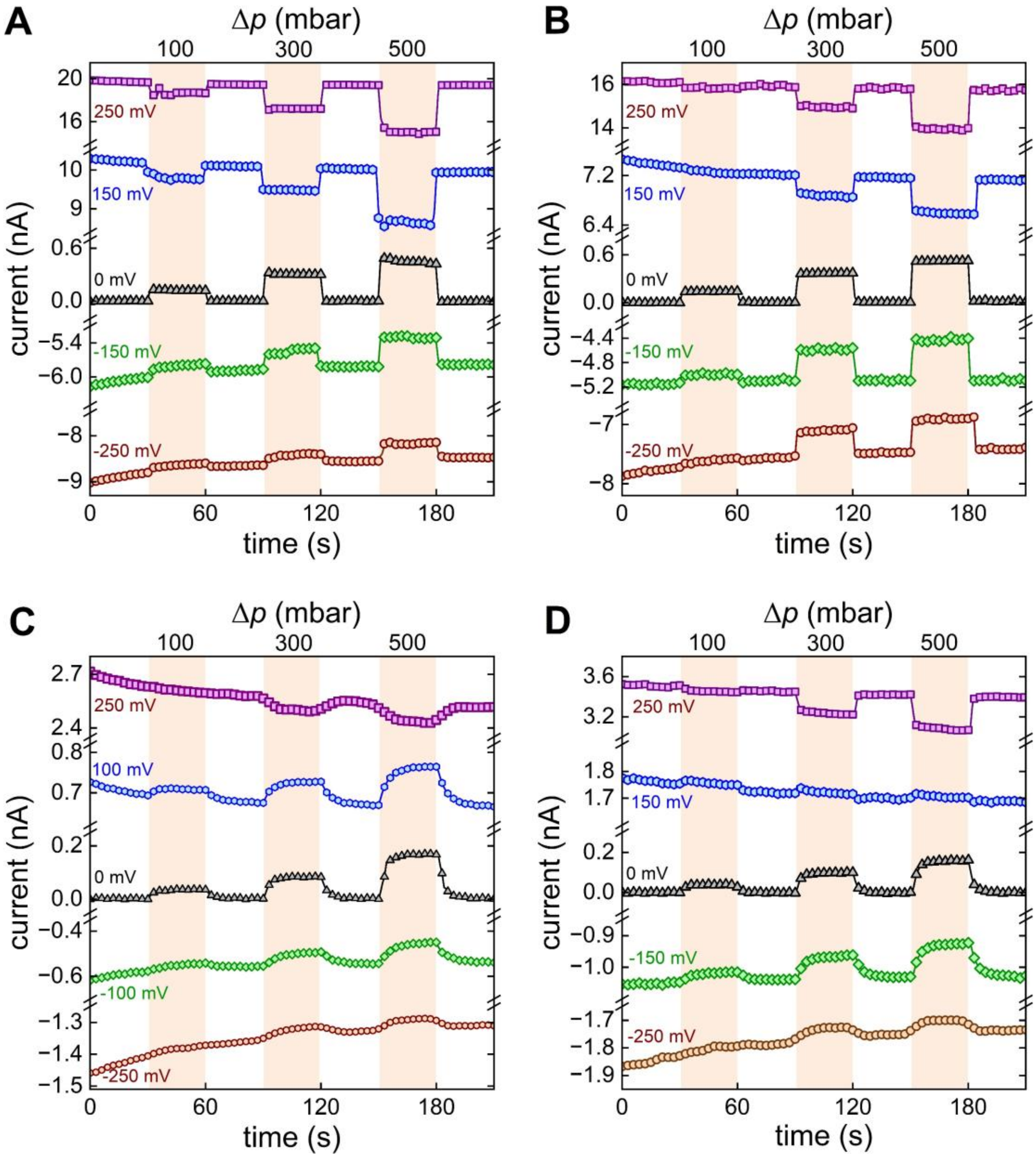

Figure S24. Cation-dependent pressure response to ion current in different electric field. Ionic current *vs* time at different Δp and ΔV for (**A**) KCl and (**B**) NaCl, (**C**) $CaCl_2$ and (**D**) $MgCl_2$. For KCl and NaCl, C_0 = 1 mM; and $CaCl_2$ and $MgCl_2$, C_0 = 0.5 mM aqueous electrolytes are used to maintain an equivalent ionic strength of 1 mN. *Device #8* is used in this figure.

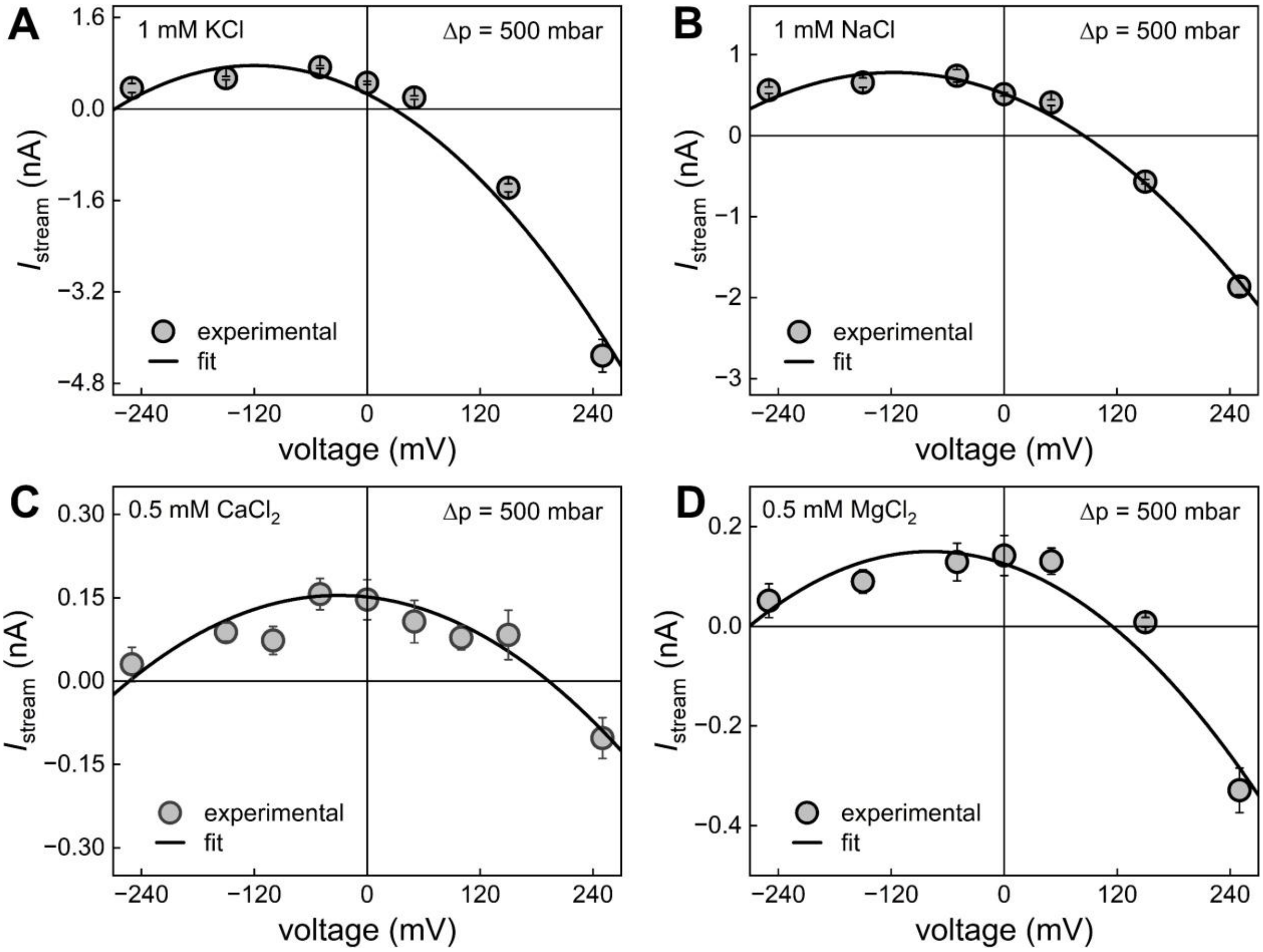

Figure S25. I_{stream}–V curves at Δp = 500 mbar for (**A**) KCl and (**B**) NaCl, (**C**) $CaCl_2$ and (**D**) $MgCl_2$. To maintain an equivalent ionic strength of 1 mN, KCl and NaCl, C_0 = 1 mM; and $CaCl_2$ and $MgCl_2$, C_0 = 0.5 mM aqueous electrolytes are used. *Device #8* is used in this figure. The black lines represent fits to the experimental data using Equation 1 of the manuscript. Error bars represent the uncertainty in the measured I_{stream} for a particular Δp.

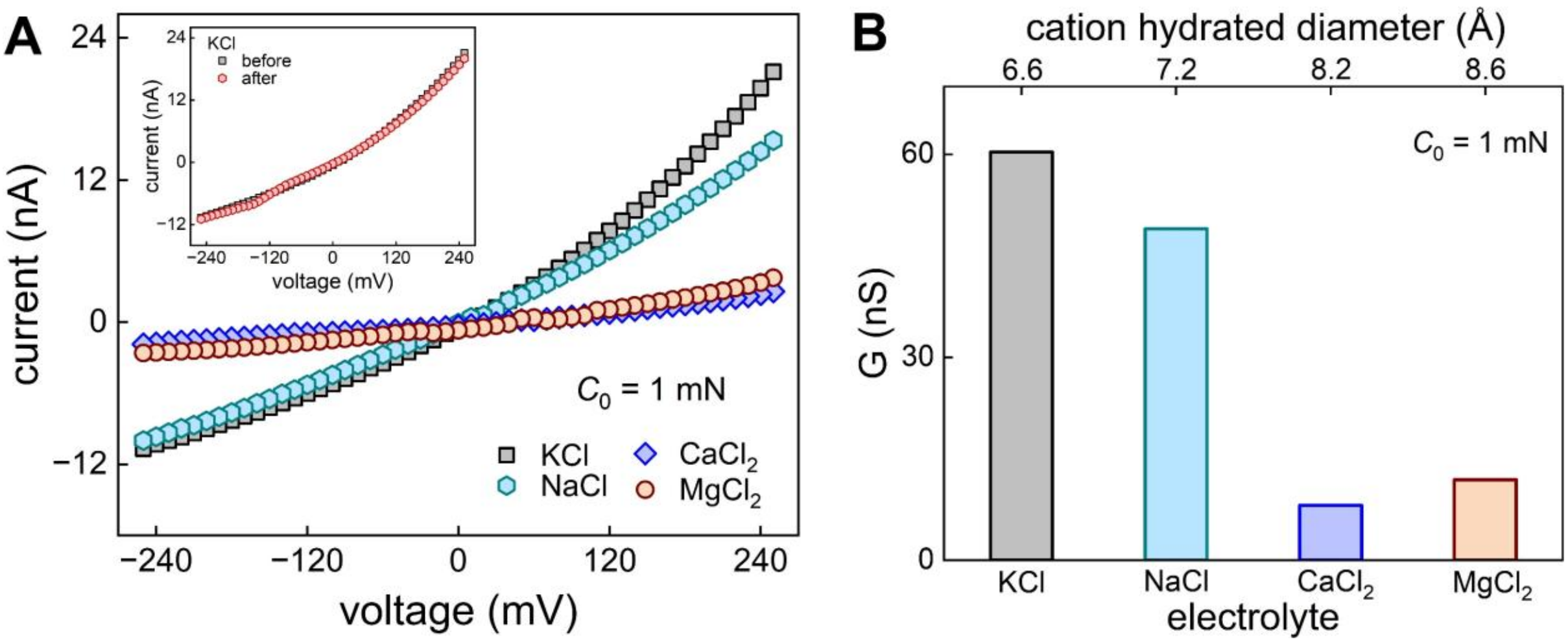

Figure S26. Salt-dependent ion transport through a VM channels device. Comparison of (**A**) *IV*s and (B) corresponding *G* of a VM device with 1 mN KCl, NaCl, $CaCl_2$ and $MgCl_2$. Inset A compares the IVs (with 1 mN KCl) before and after using NaCl followed by $CaCl_2$ and $MgCl_2$. *Device #8* is used in this figure.

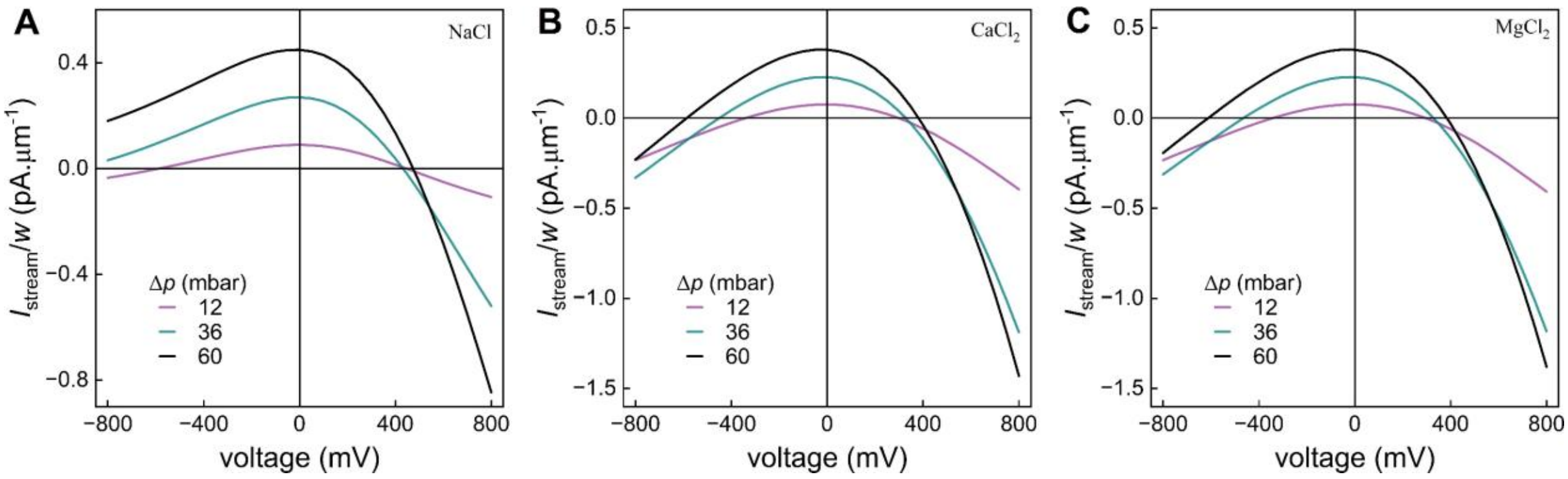

Figure S27. Numerically simulated streaming current per unit channel width (*I*stream/*w*) with ΔV at different $\Delta \boldsymbol{p}$ (**A**) NaCl, (**B**) $CaCl_2$ and (**C**) $MgCl_2$. The θ is considered as -4 mC.m^{-2} and C_0 = 1 mM.

S14. Cyclic and long-term stability measurements.

To evaluate the cyclic stability and durability of the devices, the ionic current response to the applied Δp was monitored before and after series of experiments in the absence of an external electric field using two devices: *device #4* and *#8*.

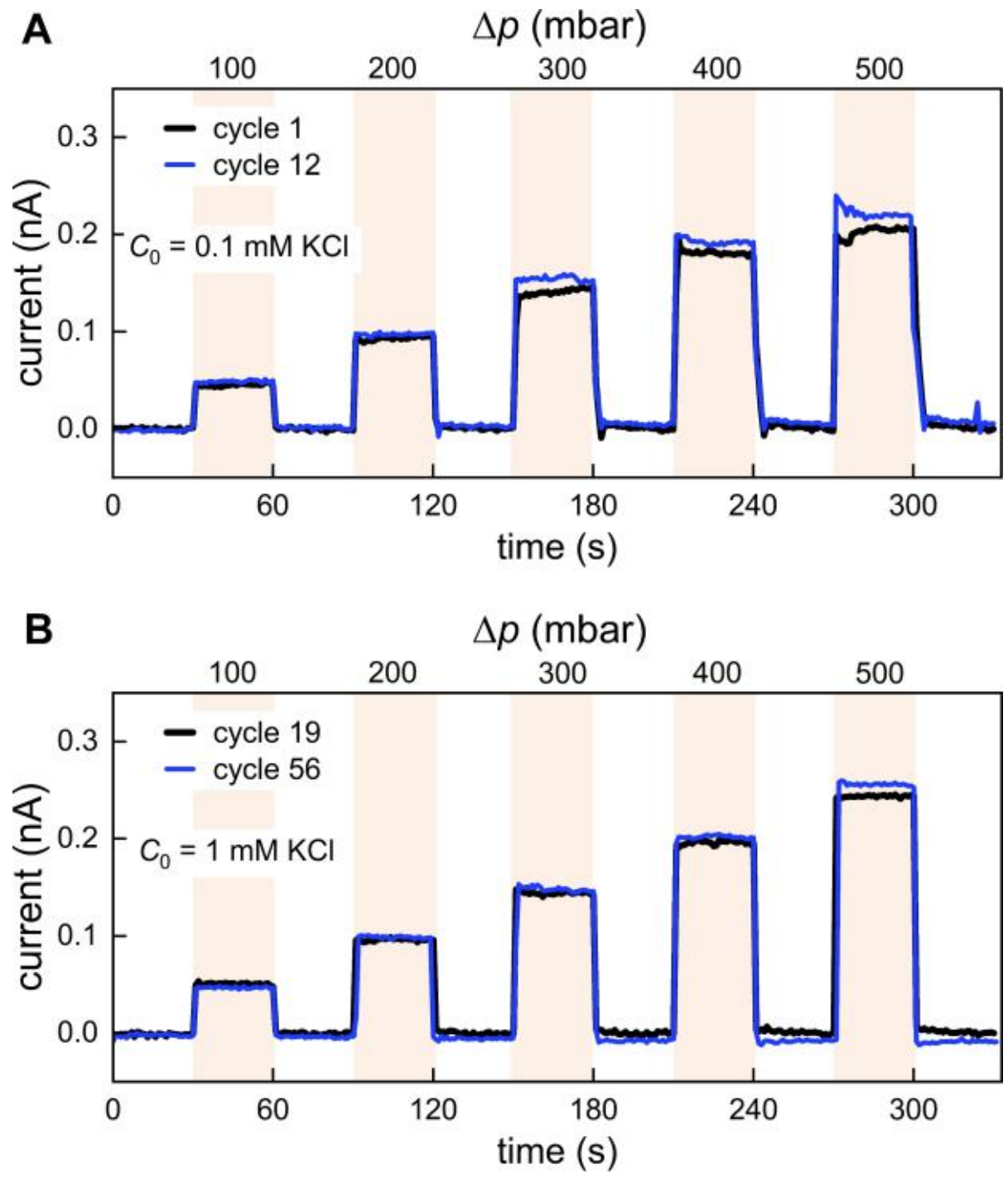

Figure S28. Stability of VM device after different KCl concentrations. Comparison of Δp-dependent ionic current measured in (**A**) 0.1 mM KCl (**B**) 1 mM KCl in the absence of an applied electric field at different cycling number. Device #4 is used in this figure.

The *device #4* was subjected to a series of I_{stream} measurements at different ΔV and C_0. For each electrolyte concentration (0.1, 1, 10, and 100 mM KCl), measurements were performed at ΔV = 0, 50, 100, 150, 200, and 250 mV at both positive and negative polarities. The stability assessment was initiated in 0.1 mM KCl by measuring the Δp-dependent I_{stream} at ΔV = 0, followed by I_{stream}–V measurements (Figure S28), and subsequently repeating the Δp-dependent I_{stream} at ΔV = 0. This procedure corresponded to a total of 12 measurement cycles. The similarity in the ionic current responses obtained in the 1st and 12th cycles (Figure S9A) confirms the stability of the device under repeated pressure and electric-field changes. The electrolyte concentration was then increased to 1 mM KCl (Figure S8A), and the same measurement sequence was repeated. Identical procedures were subsequently performed in 10 (Figure S10A) and 100 mM KCl (Figure S10C), resulting in ~55

measurement cycles in total. Upon completion of all measurements, the device was thoroughly rinsed with DI water and the Δp-dependent I_{stream} at $\Delta V = 0$ in 1 mM KCl. The similar pressure-response characteristics observed before and after the complete measurement sequence (Figure S28B) demonstrate the excellent cyclic and long-term stability of the device.

The cyclic and long-term stability of VM *device #8* was further evaluated in different electrolyte solutions (Figure S29). For each electrolyte, the Δp-dependent ionic current was first recorded at $\Delta V = 0$, followed by I_{stream}–V measurements, and finally remeasured at $\Delta V = 0$. The similar initial and final pressure-response curves demonstrate the cyclic stability of the device in all tested electrolytes, including 1 mM KCl and NaCl (Figures S29A and S29B) and 0.5 mM $CaCl_2$ and $MgCl_2$ (Figures S29C and S.29D). To further assess device robustness following exposure to different electrolyte, pressure gradients, and electric fields, the Δp-dependent ionic current was remeasured in 1 mM KCl at $\Delta V = 0$ after completion of all experiments. The similar final pressure-response curves with the initial one (Figure S29E) confirms that the device remained stable throughout the testing protocol.

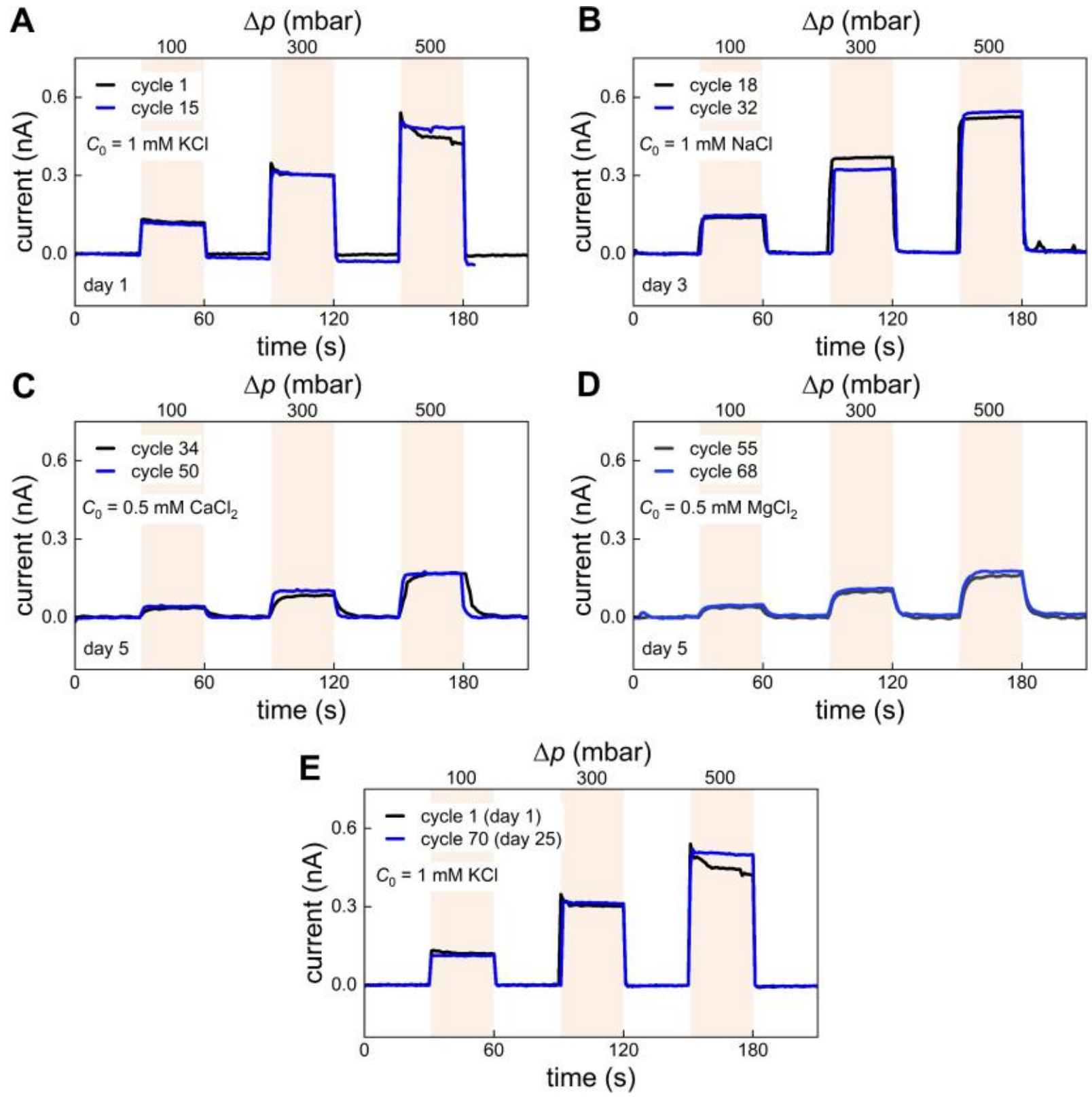

Figure S29. Stability of VM device at different electrolytes. Comparison of Δp-dependent ionic current measured with (**A**) 1 mM KCl, (**B**) 1 mM NaCl, (**C**) 0.5 mM $CaCl_2$ (**D**) 0.1 mM $MgCl_2$ and (**E**) 1 mM KCl at different cycling number (at $\Delta V = 0$). *Device #8* is used in this figure.

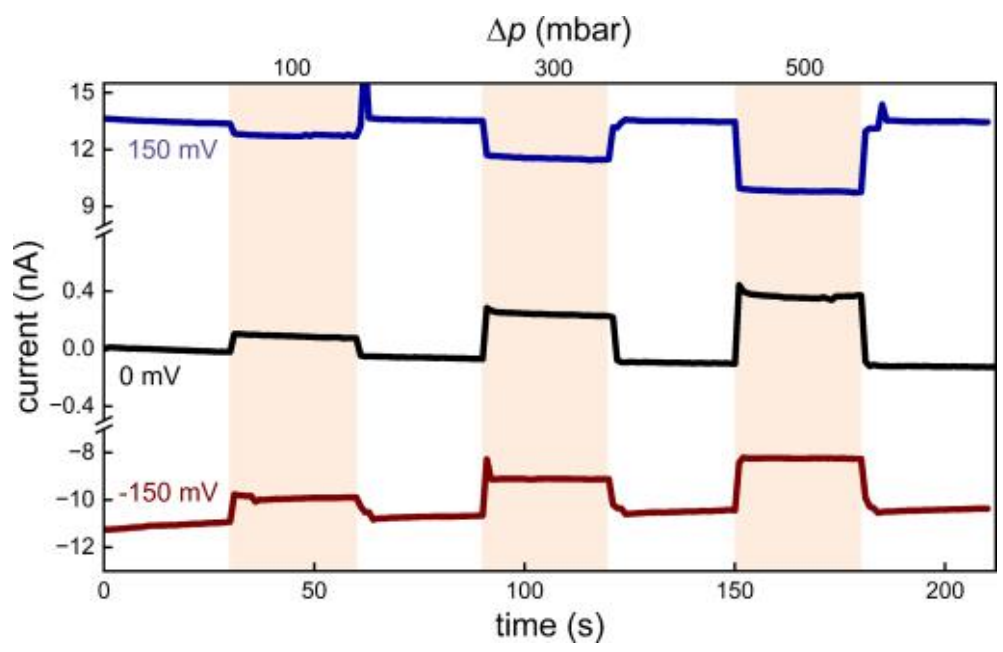

Figure S30. Pt electrode: Comparison of the Δp-dependent ionic current measured at $\Delta V = 0$, +150, and -150 mV using 1 mM KCl with Pt mesh electrodes.

S15. Repeatability of I_{stream}–V with different devices

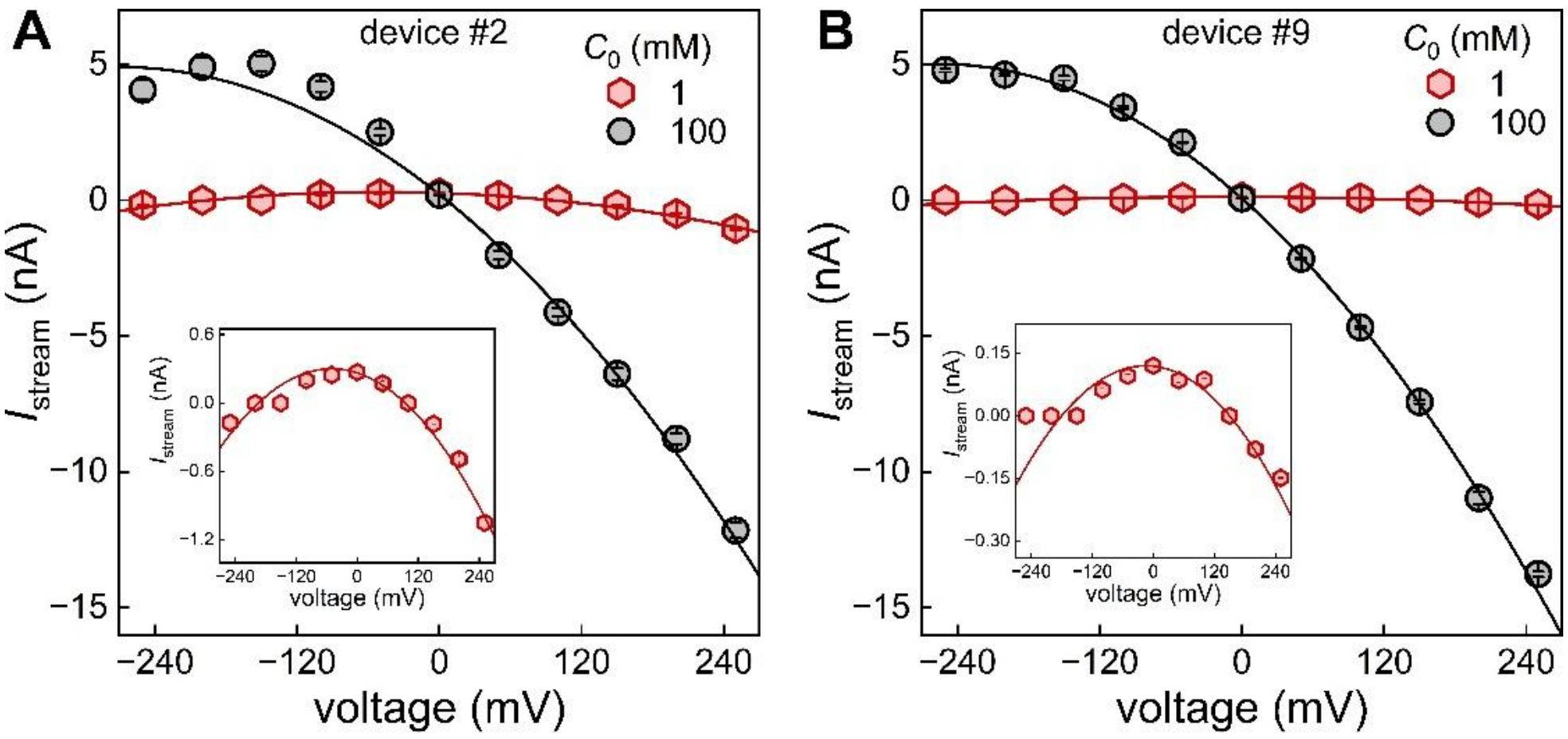

Figure S31. I_{stream}-V curves for (**A**) device #2 and (**B**) device #9 at Δp = 500 mbar for C_0 = 1 and 100 mM KCl. The solid lines represent fits to the experimental data using Equation 1 of the manuscript. Error bars represent the uncertainty in the measured I_{stream} for a particular Δp.

The ΔV-induced modulation of I_{stream} was observed in multiple VM devices. Figures S31A and S31B show the I_{stream}–V curves measured using *device #2* and *device #9*, respectively, at C_0 of 1 and 100 mM KCl at 500 mbar. Figure S25 shows the I_{stream}–V curves measured *device #8* with KCl, NaCl, $CaCl_2$ and $MgCl_2$ electrolytes, each at an equivalent concentration 1 mN.

References:

[1] Zou, Y.-C., Mogg, L., Clark, N., et al., "Ion exchange in atomically thin clays and micas," *Nature Materials* 20 (2021): 1677-1682, https://doi.org/10.1038/s41563-021-01072-6.

[2] a)Collins, D. R., Fitch, A. N., Catlow, C. R. A., "Dehydration of vermiculites and montmorillonites: a time-resolved powder neutron diffraction study," *Journal of Materials Chemistry* 2 (1992): 865-873 , https://doi.org/10.1039/JM9920200865; b) Ma, L., Su, X., Xi, Y., et al., "The structural change of vermiculite during dehydration processes: A real-time in-situ XRD method," *Applied Clay Science* 183 (2019): 105332, https://doi.org/https://doi.org/10.1016/j.clay.2019.105332.

[3] Razmjou, A., Eshaghi, G., Orooji, Y., et al., "Lithium ion-selective membrane with 2D subnanometer channels," *Water Research* 159 (2019): 313-323, https://doi.org/https://doi.org/10.1016/j.watres.2019.05.018.

[4] a) Frisenda, R., Navarro-Moratalla, E., Gant, P., et al., "Recent progress in the assembly of nanodevices and van der Waals heterostructures by deterministic placement of 2D materials," *Chemical Society Reviews* 47 (2018): 53-68, https://doi.org/10.1039/C7CS00556C; b) Wang, X., Kang, K., Chen, S., Du, R., Yang, E.-H., "Location-specific growth and transfer of arrayed MoS2 monolayers with controllable size," *2D Materials* 4 (2017): 025093, https://doi.org/10.1088/2053-1583/aa6e69; c) Bhardwaj, A., Surmani Martins, M. V., You, Y., et al., "Fabrication of angstrom-scale two-dimensional channels for mass transport," *Nature Protocols* 19 (2024): 240-280, https://doi.org/10.1038/s41596-023-00911-x.

[5] a) Lee, S. W., Kim, H. J., Kim, D.-K., "Power Generation from Concentration Gradient by Reverse Electrodialysis in Dense Silica Membranes for Microfluidic and Nanofluidic Systems," *Energies* 9 (2016): 49, https://www.mdpi.com/1996-1073/9/1/49.; b) Bhardwaj, A., Gogoi, R. K., Howard, W. J., et al., "Ultramicrotomy-Assisted Fabrication of Nanochannels for Efficient Ion Transport and Energy Generation," *Advanced Functional Materials* 34 (2024): 2401988, https://doi.org/https://doi.org/10.1002/adfm.202401988.

[6] Myers, T. G., MacDevette, M. M., Font, F., Cregan, V., "Continuum mathematics at the nanoscale," *Journal of Mathematics in Industry* 4 (2014): 11, https://doi.org/10.1186/2190-5983-4-11.

[7] a) Marcotte, A., Mouterde, T., Niguès, A., Siria, A., Bocquet, L., "Mechanically activated ionic transport across single-digit carbon nanotubes," *Nature Materials* 19 (2020): 1057-1061, https://doi.org/10.1038/s41563-020-0726-4; b) Ma, L., Li, Z., Yuan, Z., Huang, C., Siwy, Z. S., Qiu, Y., "Modulation of Ionic Current Rectification in Ultrashort Conical Nanopores," *Analytical Chemistry* 92, (2020) 16188-16196, https://doi.org/10.1021/acs.analchem.0c03989.

[8] Jiang, X., Zhao, C., Noh, Y., et al., "Nonlinear electrohydrodynamic ion transport in graphene nanopores," *Science Advances* 8, (2022): eabj2510, https://doi.org/doi:10.1126/sciadv.abj2510.

[9] a) Schlaich, A., Knapp, E. W., Netz, R. R., "Water Dielectric Effects in Planar Confinement," *Physical Review Letters* 117, (2016): 048001, https://doi.org/10.1103/PhysRevLett.117.048001; b) Fumagalli, L., Esfandiar, A., Fabregas, R., et al., "Anomalously low dielectric constant of confined water," *Science* 360, (2018): 1339-1342, https://doi.org/doi:10.1126/science.aat4191.

[10] Paul, A., Mukherjee, S., Dhar, J., Ghosal, S., Chakraborty, S., "The effect of the finite size of ions and Debye layer overspill on the screened Coulomb interactions between charged flat plates," *ELECTROPHORESIS* 41, (2020): 607-614, https://doi.org/https://doi.org/10.1002/elps.201900318.
